# PSAT1 integrates ferroptosis resistance and immune evasion to drive hepatocellular carcinoma progression

**DOI:** 10.1126/sciadv.aei7877

**Published:** 2026-10-28

**Authors:** Chi Zhang, Yue Yang, Wei Tian, Jia Zhang, Chao Ge, Lili Meng, Yuqi Liu, Zhujun Wei, Hua Tian

**Affiliations:** ^1^ State Key Laboratory of Systems Medicine for Cancer, Shanghai Cancer Institute, Renji Hospital, Shanghai Jiao Tong University School of Medicine, Shanghai 200032, China.; ^2^ Department of Pathology, Zhongshan Hospital, Fudan University, Shanghai 200032, China.

## Abstract

The serine/glycine biosynthesis pathway plays a critical role in tumor progression and has emerged as a promising therapeutic target in cancer. Phosphoserine aminotransferase 1 (PSAT1) is a key enzyme in this pathway and has been implicated in the progression of multiple malignancies. However, the functional significance and underlying mechanisms of PSAT1 in hepatocellular carcinoma (HCC) remain largely undefined. In this study, we identify PSAT1 as a critical determinant of ferroptosis resistance in HCC. Elevated PSAT1 expression was positively associated with ferroptosis resistance and poor clinical outcomes in patients with HCC. Both in vitro and in vivo functional assays demonstrated that PSAT1 promotes HCC cell proliferation and suppresses ferroptosis through an HSPA5-mediated adaptive endoplasmic reticulum response. Mechanistically, PSAT1 interacts with and stabilizes BCLAF1, thereby enhancing HSPA5 messenger RNA stability and translation in HCC cells. In addition, PSAT1 remodels the tumor immune microenvironment by promoting M2 tumor-associated macrophage polarization and regulatory T cell infiltration. PSAT1 up-regulates NRF2 expression, which directly binds to the CCL5 promoter to enhance CCL5 production and secretion by tumor cells, thereby fostering an immunosuppressive microenvironment. Therapeutically, pharmacological inhibition of PSAT1 with aminooxyacetic acid markedly potentiated the antitumor efficacy of anti–PD-1 immunotherapy. Our study reveals a previously unrecognized role of PSAT1 in coordinating ferroptosis resistance and immune escape in HCC and highlights therapeutic targeting of PSAT1 as a promising strategy for HCC treatment.

## INTRODUCTION

The prognosis of patients with hepatocellular carcinoma (HCC) remains poor (*1*), as most cases are diagnosed at advanced stages and current treatment strategies, including locoregional therapy, targeted agents, and immune checkpoint blockade, provide only limited survival benefit in the overall population (*2*). In particular, low and often unsustained responses to immunotherapy, together with frequent therapeutic resistance, remain major obstacles in HCC management (*3*).

Ferroptosis, an iron-dependent form of regulated cell death driven by lipid peroxidation (*4*), has emerged as a potential antitumor strategy, with growing evidence that ferroptosis induction can restrict tumor growth and may enhance antitumor immunity and sensitize tumors to systemic therapies, including immune checkpoint blockade (*5*). However, HCC cells can develop ferroptosis resistance, which limits the efficacy of ferroptosis-based approaches and may further facilitate immune evasion. Therefore, defining tumor-intrinsic programs that enforce ferroptosis resistance and clarifying how they influence tumor progression and the immune landscape may enable rational combination strategies to overcome therapeutic failure in HCC.

The hepatic tumor microenvironment is characterized by metabolic stress, hypoxia, oxidative damage, and proteotoxic burden, driven by high proliferative demands and oncogenic signaling (*6*). Under these conditions, cancer cells experience persistent endoplasmic reticulum (ER) stress and frequently hijack the unfolded protein response (UPR) as an adaptive program to sustain survival and malignant progression (*7*, *8*). Sustained UPR activation has been implicated in resistance to chemotherapy, targeted therapies, and immunotherapy (*7*, *9*). However, the upstream metabolic determinants that engage adaptive ER stress responses in HCC, and how these programs intersect with ferroptosis resistance and immune remodeling, remain poorly understood.

Motivated by the limited efficacy of immunotherapy in HCC and the emerging recognition of ferroptosis resistance as a fundamental tumor survival strategy, we sought to identify stress-adaptive regulators that enable cancer cells to withstand ferroptotic pressure while evading immune surveillance. Through an early-stage screening approach, we identified phosphoserine aminotransferase 1 (PSAT1) as a gene robustly induced under ferroptotic stress. Although PSAT1 is classically characterized as a metabolic enzyme in de novo serine biosynthesis and has been linked to tumor progression in other cancer types (*10*–*13*), its role in ferroptosis resistance and tumor immune regulation in HCC remains unknown. Here, we systematically investigate the role of PSAT1 in coordinating ferroptosis resistance, adaptive ER stress responses, and remodeling of the tumor immune microenvironment and explore the therapeutic potential of targeting this axis to sensitize HCC to immunotherapy.

## RESULTS

### Identification of PSAT1 as a ferroptosis resistance in HCC

To identify genes linked to ferroptosis resistance in HCC, we performed RNA sequencing (RNA-seq) on HCC cells undergoing erastin-induced ferroptosis and intersected differentially expressed genes with metabolism-related enzymes, identifying *PSAT1* as a top up-regulated candidate (Fig. 1A). Consistently, *PSAT1* expression correlated with resistance to ferroptosis inducers in the Cancer Therapeutics Response Portal (Fig. 1B) and was significantly higher in sorafenib nonresponders than responders in the GSE109211 cohort (Fig. 1C).

**Fig. 1.**
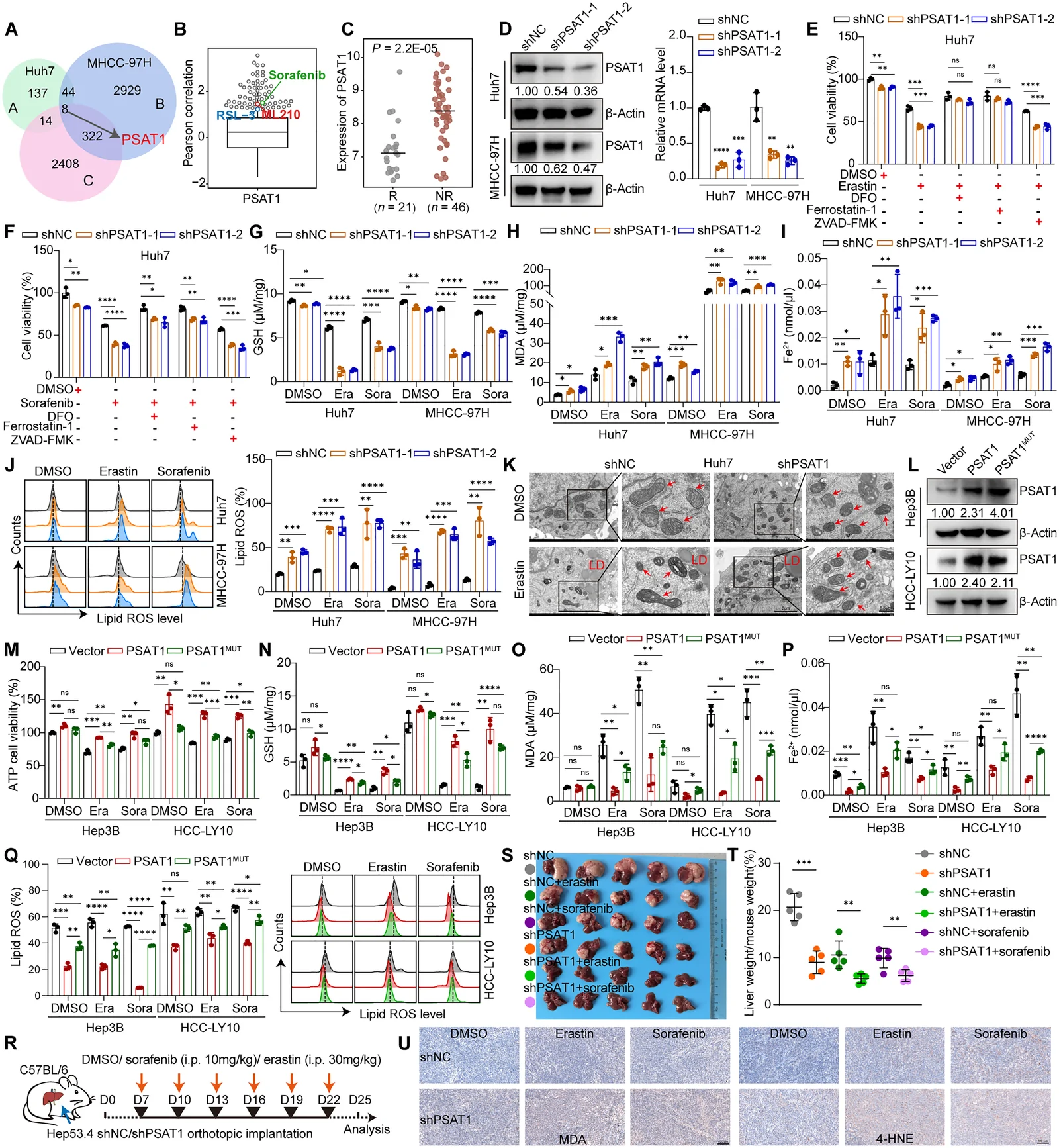
PSAT1 confers ferroptosis resistance in HCC. (**A**) Overlap of differentially expressed genes and metabolic enzymes identifies PSAT1. (**B**) Cancer Therapeutics Response Portal (CTRP) data: *PSAT1* correlates with resistance to RSL3, ML162, and sorafenib. (**C**) *PSAT1* levels in sorafenib responders (R) versus nonresponders (NR) (GSE109211). (**D**) PSAT1 expression in HCC lines [immunoblotting (IB)/quantitative polymerase chain reaction (qPCR)]. (**E** and **F**) Viability of Huh7 (shPSAT1) treated with erastin/sorafenib ± ferrostatin-1 (Fer-1), DFO, or Z-VAD. DMSO, dimethyl sulfoxide. (**G** to **J**) Intracellular GSH, MDA, Fe^2+^, and lipid ROS levels. (**K**) Transmission electron microscopy: mitochondrial damage in PSAT1-knockdown cells + erastin. (**L**) PSAT1 levels in Hep3B/HCC-LY10 expressing wild-type (WT) or mutant (D100A) PSAT1. (**M** to **Q**) WT versus mutant PSAT1 effects on viability, redox, and lipid peroxidation postinduction. (**R**) Orthotopic Hep53.4 HCC model and treatment schedule in C57BL/6 mice. i.p., intraperitoneally; D, day. (**S** and **T**) Representative liver tumors and liver weight/body weight ratios. (**U**) Representative 4-HNE/MDA staining of orthotopic tumors. Data are presented as the means ± SD. *P < 0.05; **P < 0.01; ***P < 0.001; ****P < 0.0001. ns, not significant.

Consistently, PSAT1 overexpression increased the IC_50_ (median inhibitory concentration) values of HCC cells for erastin, sorafenib, and lenvatinib (fig. S1, A to C). *PSAT1* expression was also elevated in lenvatinib-resistant HCC cells in independent datasets (fig. S1, D and E), while treatment with all three agents induced PSAT1 expression at both the mRNA and protein levels (fig. S1, F to H), suggesting stress-responsive up-regulation of PSAT1 during ferroptotic and therapeutic stress.

Gain- and loss-of-function studies in Huh7 and MHCC-97H demonstrated that PSAT1 knockdown reduced cell viability and intracellular glutathione (reduced form) (GSH) levels while increasing lipid peroxidation, iron accumulation, and malondialdehyde (MDA) production (Fig. 1, D to J, and fig. S1, I and J). PSAT1 knockdown markedly sensitized HCC cells to erastin- and sorafenib-induced cell death, effects reversed by ferrostatin-1 or deferoxamine (DFO), but not by the apoptosis inhibitor Z-VAD-FMK, indicating a ferroptosis-dependent mechanism (Fig. 1, E and F, and fig. S1, K and L). Transmission electron microscopy further revealed characteristic ferroptotic mitochondrial alterations upon PSAT1 knockdown, which were exacerbated by erastin treatment (Fig. 1K and fig. S1M). Conversely, ectopic PSAT1 expression attenuated ferroptosis induced by erastin or sorafenib, as evidenced by preserved viability, increased GSH levels, reduced lipid peroxidation, and decreased iron accumulation (Fig. 1, L to Q).

To determine the contribution of PSAT1 enzymatic activity, we reconstituted PSAT1-depleted cells with wild-type PSAT1 or the catalytically inactive PSAT1 mutant [Asp^100^→Ala (D100A)] (*14*). Although PSAT1-D100A partially mitigated ferroptotic damage, its protective effect was substantially weaker than that of wild-type PSAT1 (Fig. 1, L to Q). Consistent with this difference, metabolomic profiling showed that PSAT1 knockdown broadly disrupted amino acid, nitrogen, polyamine, redox, and central carbon metabolism, changes that were largely restored by wild-type PSAT1 but only partially rescued by PSAT1-D100A (fig. S2, A and B). Intracellular α-ketoglutarate (α-KG) levels showed a similar pattern (fig. S2C). These findings indicate that PSAT1 enzymatic activity is important for maintaining metabolic homeostasis but does not fully account for its ferroptosis-protective effect.

Given the partial protective effect of the catalytically inactive PSAT1-D100A mutant, we next examined whether PSAT1 regulates GPX4 through mechanisms beyond its canonical enzymatic activity. PSAT1 knockdown markedly reduced GPX4 protein abundance, whereas PSAT1 overexpression increased GPX4 protein levels without significantly altering *GPX4* mRNA expression, indicating predominantly posttranscriptional regulation (fig. S2, D to F). Moreover, wild-type PSAT1 restored GPX4 protein expression more effectively than the PSAT1-D100A mutant (fig. S2G), suggesting that both catalytic and noncatalytic functions of PSAT1 may contribute to GPX4 regulation.

Last, in an orthotopic C57BL/6 HCC model, PSAT1 knockdown significantly reduced tumor burden and further enhanced the antitumor effects of erastin and sorafenib (Fig. 1, R to T). Consistently, MDA and 4-HNE (4-hydroxynonenal) staining was increased following PSAT1 depletion, particularly in combination with ferroptosis-inducing treatment, supporting enhanced lipid peroxidation in vivo (Fig. 1U). Similar tumor-suppressive effects were also observed in the subcutaneous nude mouse model (fig. S2, H to L).

### PSAT1 facilitates the malignant progression of HCC

Given that ferroptosis resistance is increasingly recognized as a driver of tumor progression (*5*), we examined whether PSAT1-mediated ferroptosis resistance contributes to malignant progression of HCC. In vitro, PSAT1 knockdown in Huh7 and MHCC-97H cells significantly impaired cell proliferation and clonogenic growth (Fig. 2A). These inhibitory effects were partially rescued by the ferroptosis inhibitors ferrostatin-1 and DFO, accompanied by a marked reduction in the elevated lipid reactive oxygen species (ROS) induced by PSAT1 depletion (fig. S3, A to F). Conversely, ectopic PSAT1 expression in Hep3B and HCC-LY10 cells markedly enhanced proliferative and clonogenic capacity (Fig. 2B). In vivo, PSAT1 overexpression robustly promoted tumor growth, as evidenced by increased tumor volume and weight, accelerated growth kinetics, and elevated Ki67 staining (Fig. 2, C to F, and fig. S3, G and H).

**Fig. 2.**
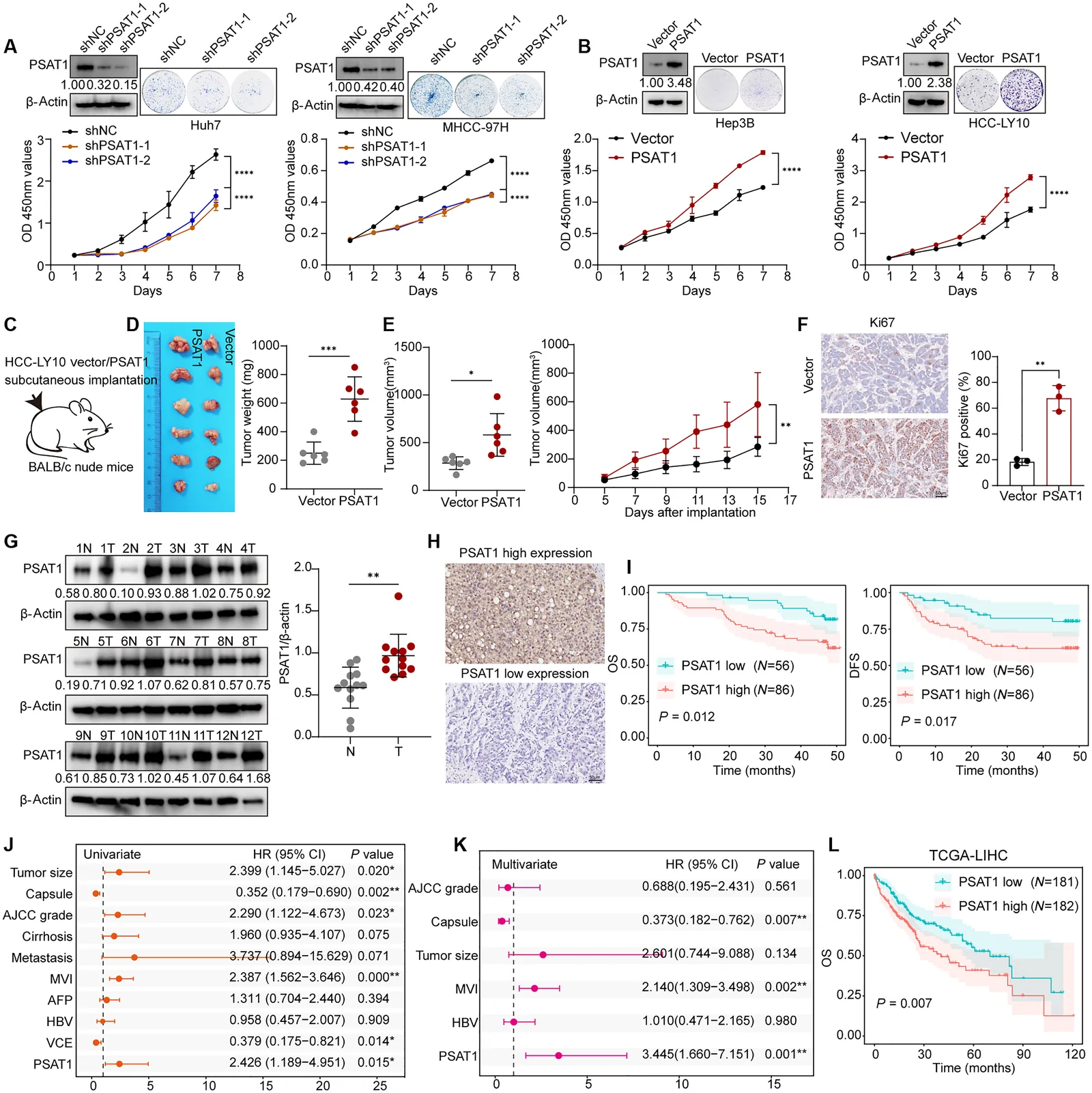
PSAT1 promotes HCC cell proliferation and is associated with poor prognosis in patients with HCC. (**A** and **B**) Proliferation and clonogenicity of HCC cells with PSAT1 knockdown or overexpression. OD, optical density. (**C** to **E**) Subcutaneous xenograft model (HCC-LY10): representative tumors, growth curves, and final weights/volumes. (**F**) Ki67 IHC staining and quantification in xenografts. (**G**) PSAT1 expression in paired human HCC and adjacent nontumorous liver tissues. (**H**) Representative IHC of low/high PSAT1 in HCC samples. (**I** to **K**) Kaplan-Meier OS/DFS curves and Cox regression analyses for PSAT1 prognostic value. HR, hazard ratio; CI, confidence interval. (**L**) TCGA-LIHC OS stratified by PSAT1. Data are presented as the means ± SD. *P < 0.05; **P < 0.01; ***P < 0.001; ****P < 0.0001.

Clinically, PSAT1 expression was significantly higher in HCC tissues than in adjacent nontumorous liver tissues (Fig. 2G). High PSAT1 expression was associated with significantly poorer overall survival (OS) and disease-free survival (DFS) and was identified as an independent predictor of adverse prognosis by multivariate Cox regression analysis (Fig. 2, H to K). These associations were independently validated in The Cancer Genome Atlas Liver Hepatocellular Carcinoma (TCGA-LIHC) cohort, in which elevated PSAT1 expression similarly correlated with reduced OS (Fig. 2L).

To define the cellular distribution of PSAT1, we reanalyzed the single-cell RNA-seq dataset GSE292298 and annotated malignant and nonmalignant cell populations. *PSAT1* expression was predominantly enriched in malignant hepatocytes, as defined by inferred copy number variations, with minimal expression in nonmalignant cell types (fig. S4, A to D). Together, these findings indicate that PSAT1 promotes HCC progression, at least in part, by protecting tumor cells from ferroptotic stress.

### Aminotransferase inhibition with AOA enhances ferroptosis-based therapeutic responses in HCC

Aminooxyacetic acid (AOA), a broad-spectrum aminotransferase inhibitor that can inhibit PSAT1 enzymatic activity (*10*, *12*), was next evaluated in HCC cells. AOA treatment reduced PSAT1 mRNA and protein expression and significantly suppressed HCC cell proliferation in vitro (fig. S5, A to C). Given that AOA primarily inhibits aminotransferase activity, the observed reduction in PSAT1 expression is likely an indirect consequence of metabolic perturbation rather than a direct pharmacological effect on PSAT1 expression. Cotreatment with AOA markedly potentiated sorafenib- or erastin-induced cell death, an effect largely reversed by ferrostatin-1 or DFO, indicating ferroptosis-dependent cytotoxicity (fig. S5D). Synergy analysis using the zero interaction potency model confirmed strong synergistic interactions between AOA and either sorafenib or erastin (fig. S5, E and F). This antitumor synergy was further validated in patient-derived HCC xenograft models, where combination treatment produced significantly greater tumor growth inhibition than either monotherapy alone (fig. S5, G to J). Combination-treated tumors exhibited increased 4-HNE staining and reduced Ki67 expression, reflecting enhanced lipid peroxidation and suppressed tumor proliferation (fig. S5K). Collectively, these findings indicate that AOA-mediated aminotransferase inhibition enhances the response of HCC to ferroptosis-inducing agents, complementing our genetic evidence supporting a role for PSAT1 in ferroptosis resistance.

### PSAT1 promotes ferroptosis resistance by sustaining an HSPA5-dependent adaptive ER stress response

To elucidate how PSAT1 confers ferroptosis resistance, RNA-seq was performed in PSAT1-knockdown HCC cells under basal conditions and following erastin or sorafenib treatment. Gene Ontology (GO) analysis revealed that loss of *PSAT1* was predominantly associated with disruption of adaptive ER stress response–related pathways, including protein folding, UPR, and ER chaperone functions, both at the baseline and under ferroptotic stress (Fig. 3A and fig. S6, A and B).

**Fig. 3.**
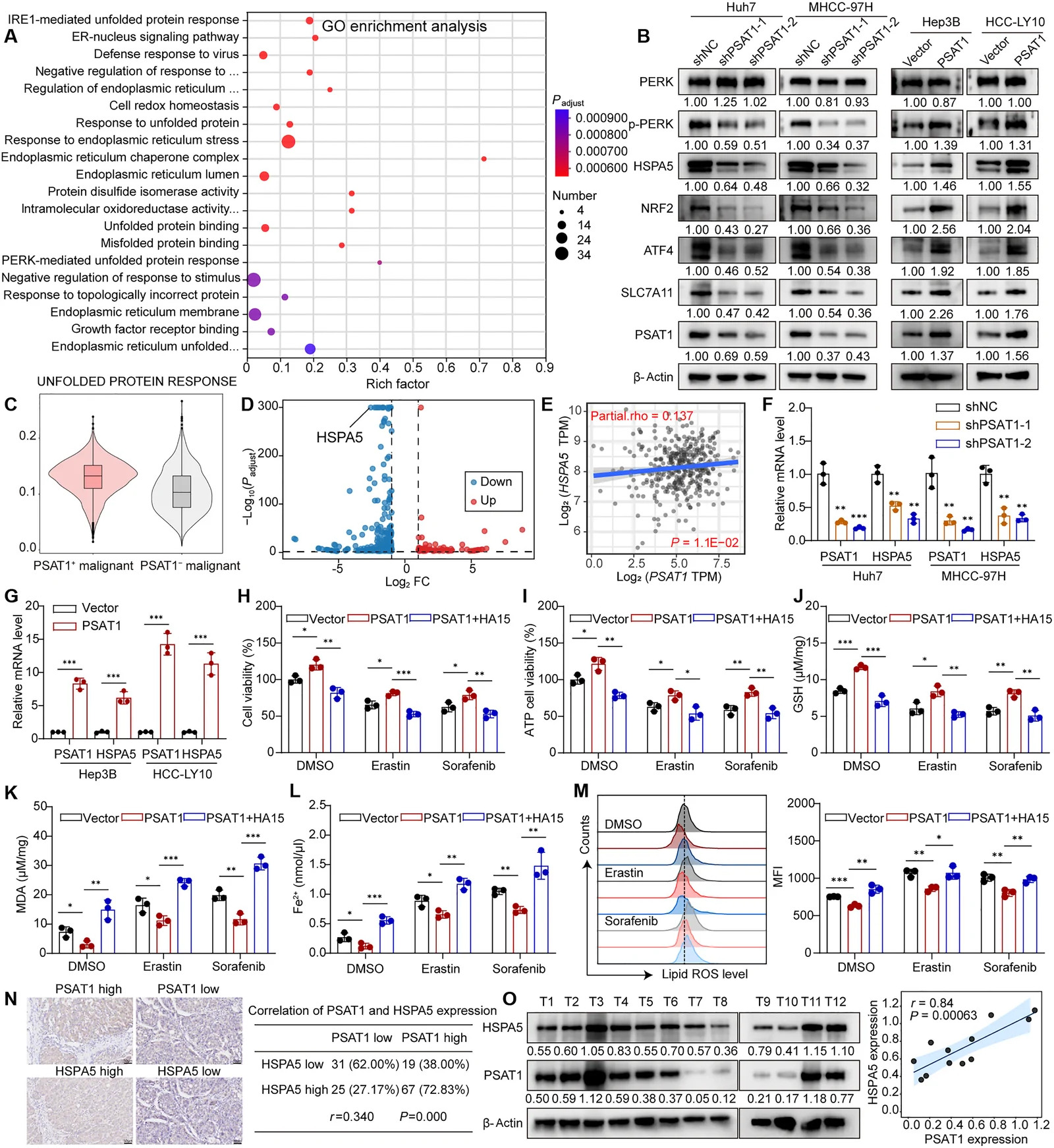
PSAT1 confers ferroptosis resistance via HSPA5-dependent ER stress adaptation. (**A**) GO enrichment of *PSAT1*-positive genes. (**B**) Immunoblots of ER stress and ferroptosis markers. (**C**) Single-cell RNA-seq UPR activity in *PSAT1*-high versus *PSAT1*-low cells. (**D**) Volcano plot of differentially expressed genes (shNC versus shPSAT1). (**E**) *PSAT1*-*HSPA5* correlation in TCGA-HCC. FC, fold change. (**F** and **G**) PSAT1 regulation of *HSPA5* mRNA. (**H** to **M**) Viability, redox, iron, and lipid peroxidation upon PSAT1 overexpression and HSPA5 inhibition (HA15) during ferroptosis. MFI, mean fluorescence intensity. (**N** and **O**) PSAT1-HSPA5 correlation in human HCC tissues (IHC and immunoblotting). Data are presented as the means ± SD. *P < 0.05; **P < 0.01; ***P < 0.001.

Consistently, PSAT1 knockdown reduced the expression of multiple adaptive ER stress–responsive genes, including *DDIT3*, *PPP1R15A*, *HYOU1*, *HERPUD1*, *SEL1L*, and *HSPA5* (fig. S6, C and D). At the protein level, PSAT1 depletion attenuated PERK phosphorylation and reduced NRF2, ATF4, HSPA5, and SLC7A11 expression, whereas PSAT1 overexpression produced the opposite effects (Fig. 3B), indicating that PSAT1 sustains a PERK-NRF2-ATF4 signaling axis to maintain ER stress adaptation and antioxidant capacity.

To determine whether this functional state is reflected at the single-cell level, malignant hepatocytes were stratified into *PSAT1*-high and *PSAT1*-low subsets. Although these populations showed overlapping UMAP (uniform manifold approximation and projection) distributions (fig. S6, E and F), *PSAT1*-high cells exhibited markedly elevated UPR activity, as assessed by AUCell analysis (Fig. 3C), supporting enhanced ER stress adaptation in *PSAT1*-expressing tumor cells.

Among adaptive ER stress components, HSPA5 (GRP78/BiP) acts as a central ER chaperone coordinating prosurvival stress signaling and ferroptosis regulation, as previously reported (*15*, *16*). Consistent with this notion, RNA-seq analysis identified *HSPA5* as one of the most significantly down-regulated genes upon PSAT1 knockdown (Fig. 3D), and TCGA-LIHC data further revealed a positive correlation between *PSAT1* and *HSPA5* expression (Fig. 3E). Consistently, *PSAT1* silencing reduced, whereas *PSAT1* overexpression increased, *HSPA5* expression in HCC cells (Fig. 3, F and G), indicating that HSPA5 expression is positively regulated by PSAT1 in HCC cells.

To determine whether HSPA5 is functionally required for PSAT1-mediated ferroptosis resistance, PSAT1-overexpressing HCC cells were treated with the HSPA5 inhibitor HA15. PSAT1 overexpression markedly suppressed erastin- or sorafenib-induced ferroptosis, as reflected by increased cell viability and ATP (adenosine 5′-triphosphate) and GSH levels together with reduced MDA, Fe^2+^, and lipid ROS accumulation; these protective effects were largely abrogated by HA15 treatment (Fig. 3, H to M). Consistently, analysis of human HCC tissues revealed a significant positive correlation between PSAT1 and HSPA5 expression, as demonstrated by tissue microarray immunohistochemistry (IHC) and immunoblotting of tumor samples (Fig. 3, N and O). Collectively, these findings identify HSPA5 as a critical downstream effector through which PSAT1 sustains an adaptive ER stress response and antioxidant capacity, thereby protecting HCC cells from ferroptosis.

### PSAT1 promotes HSPA5 mRNA stability and translation through interacting with BCLAF1 and enhancing its protein stability

As described above, PSAT1 silencing reduced *HSPA5* mRNA abundance (Fig. 3F). When de novo transcription was blocked by actinomycin D, PSAT1 knockdown significantly accelerated *HSPA5* mRNA decay (Fig. 4A), whereas luciferase reporter assays showed no effect on *HSPA5* transcriptional activity (fig. S7, A and B), indicating posttranscriptional regulation.

**Fig. 4.**
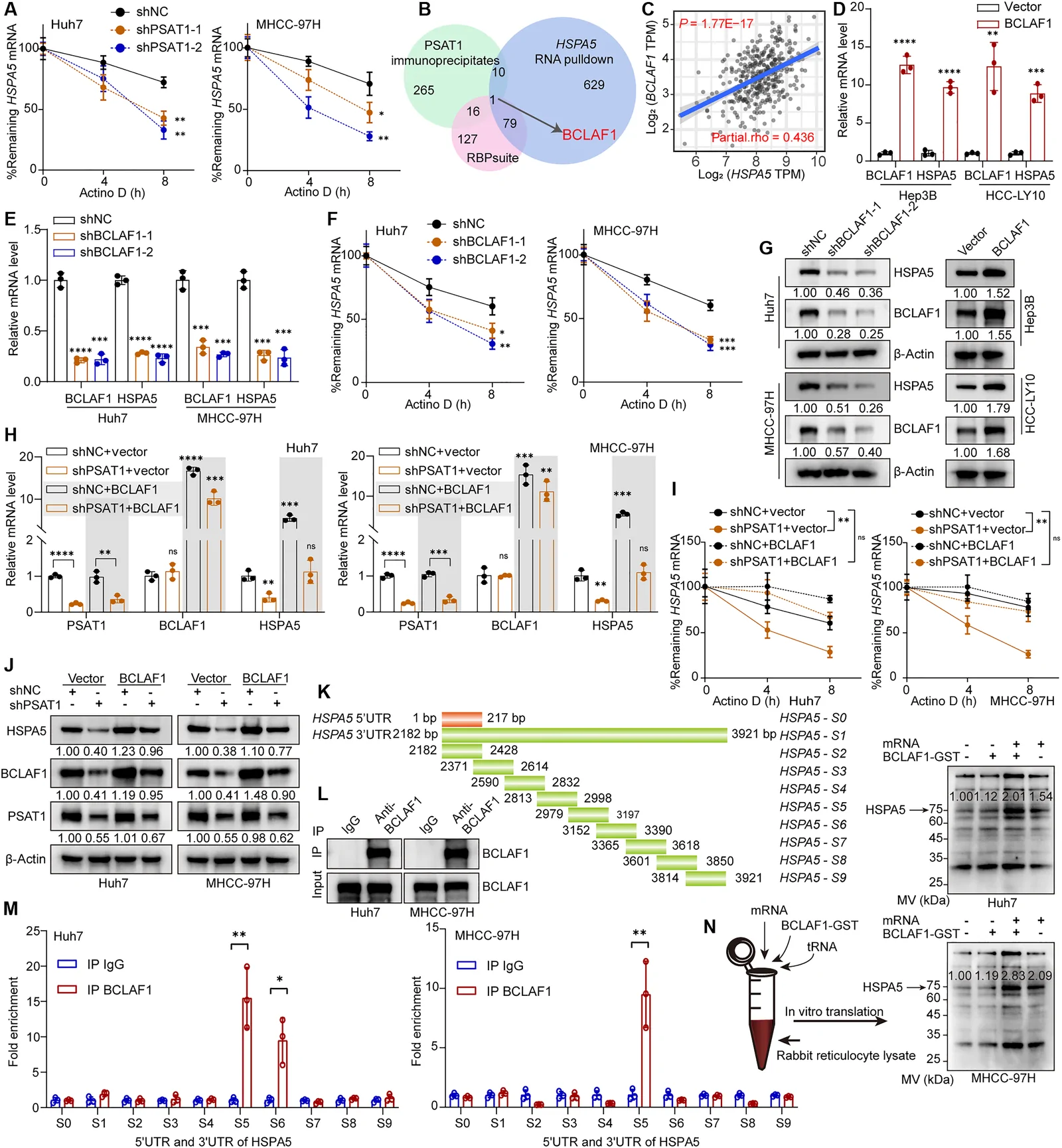
PSAT1 stabilizes and translates *HSPA5* mRNA via BCLAF1. (**A**) *HSPA5* mRNA stability (actinomycin D chase) in PSAT1-knockdown cells. h, hours. (**B**) Strategy to identify *HSPA5* mRNA binding proteins. (**C**) *BCLAF1*-*HSPA5* correlation in TCGA-HCC. (**D** to **F**) HSPA5 levels/stability upon BCLAF1 modulation. (**G**) BCLAF1 and HSPA5 protein levels after BCLAF1 manipulation. (**H** to **J**) HSPA5 expression, stability, and protein abundance with PSAT1/BCLAF1 modulation. (**K** to **M**) RIP assays confirming BCLAF1 binding to *HSPA5* mRNA. (**N**) In vitro translation of HSPA5 with recombinant BCLAF1. Data are presented as the means ± SD. *P < 0.05; **P < 0.01; ***P < 0.001; ****P < 0.0001.

To determine whether the metabolic function of PSAT1 contributes to HSPA5 regulation, we supplemented PSAT1-knockdown cells with serine or the cell-permeable α-KG derivative dimethyl-α-KG (DM-α-KG), two metabolites linked to PSAT1-dependent serine biosynthesis and aminotransferase activity. Both treatments partially restored HSPA5 mRNA and protein levels without restoring PSAT1 expression (fig. S7, C to E). Moreover, serine and DM-α-KG partially rescued the accelerated decay of *HSPA5* mRNA caused by PSAT1 depletion (fig. S7, F and G). These findings indicate that PSAT1-dependent metabolic alterations contribute, at least in part, to the maintenance of *HSPA5* mRNA stability.

However, the partial rescue by metabolic supplementation suggested that additional mechanisms may contribute to PSAT1-dependent HSPA5 regulation. As PSAT1 is predominantly localized in the cytoplasm and has not been reported to directly participate in mRNA regulatory processes, we therefore investigated whether RNA binding proteins might mediate its posttranscriptional regulation of HSPA5. An RNA pull-down assay using HSPA5 transcripts followed by mass spectrometry identified multiple candidate ribose-binding proteins (RBPs). Intersection of proteins identified in PSAT1 immunoprecipitates, HSPA5 RNA pull-down complexes, and RBP predictions from RBPsuite (*17*) narrowed the candidates to BCLAF1 (Fig. 4B). Consistently, TCGA-LIHC analysis revealed a positive correlation between BCLAF1 and HSPA5 expression (Fig. 4C).

Gain- and loss-of-function analyses demonstrated that BCLAF1 positively regulated *HSPA5* mRNA abundance, stability, and protein expression (Fig. 4, D to G). Ectopic expression of BCLAF1 in PSAT1-knockdown cells rescued the reduction in *HSPA5* mRNA levels, mRNA stability, and protein expression caused by PSAT1 depletion (Fig. 4, H to J), indicating that BCLAF1 mediates PSAT1-dependent regulation of HSPA5.

Given that RBPs regulate mRNA stability and translation through binding to untranslated regions (*18*), we mapped the BCLAF1-binding region within *HSPA5* mRNA by RNA immunoprecipitation (RIP) analysis using a series of 5′ untranslated region (5′UTR) and 3′UTR fragments (S0 to S9). BCLAF1 showed preferential enrichment at the S5 region (nucleotides 2979 to 3197) of the *HSPA5* 3′UTR, with additional enrichment of S6 observed in Huh7 cells (Fig. 4, K to M). To further assess its functional effect on translation, we performed a cell-free in vitro translation assay using *HSPA5* mRNA and recombinant BCLAF1-GST. Addition of BCLAF1-GST further increased the HSPA5 translation signal compared with *HSPA5* mRNA alone, supporting a direct role of BCLAF1 in promoting HSPA5 protein synthesis (Fig. 4N).

Given that BCLAF1 was identified by intersecting PSAT1 immunoprecipitates with *HSPA5* RNA pull-down–associated RBPs (Fig. 4B), we first examined whether PSAT1 physically interacts with BCLAF1. Coimmunoprecipitation (co-IP) and glutathione *S*-transferase (GST) pull-down assays demonstrated a direct interaction between PSAT1 and BCLAF1, which was attenuated by treatment with the PSAT1 inhibitor AOA (Fig. 5, A and B, and fig. S7H). Consistent with a functional interaction, PSAT1 knockdown reduced BCLAF1 protein levels without affecting its mRNA expression, whereas PSAT1 overexpression increased BCLAF1 protein abundance, indicating that PSAT1 regulates BCLAF1 primarily at the posttranscriptional level (Fig. 5, C and D). CHX (cycloheximide) chase assays revealed accelerated BCLAF1 degradation upon PSAT1 depletion, which was reversed by the proteasome inhibitor MG132 but not by lysosomal inhibition with chloroquine (Fig. 5, E to G). Consistently, PSAT1 knockdown markedly increased BCLAF1 ubiquitination (Fig. 5H), indicating proteasome-dependent degradation.

**Fig. 5.**
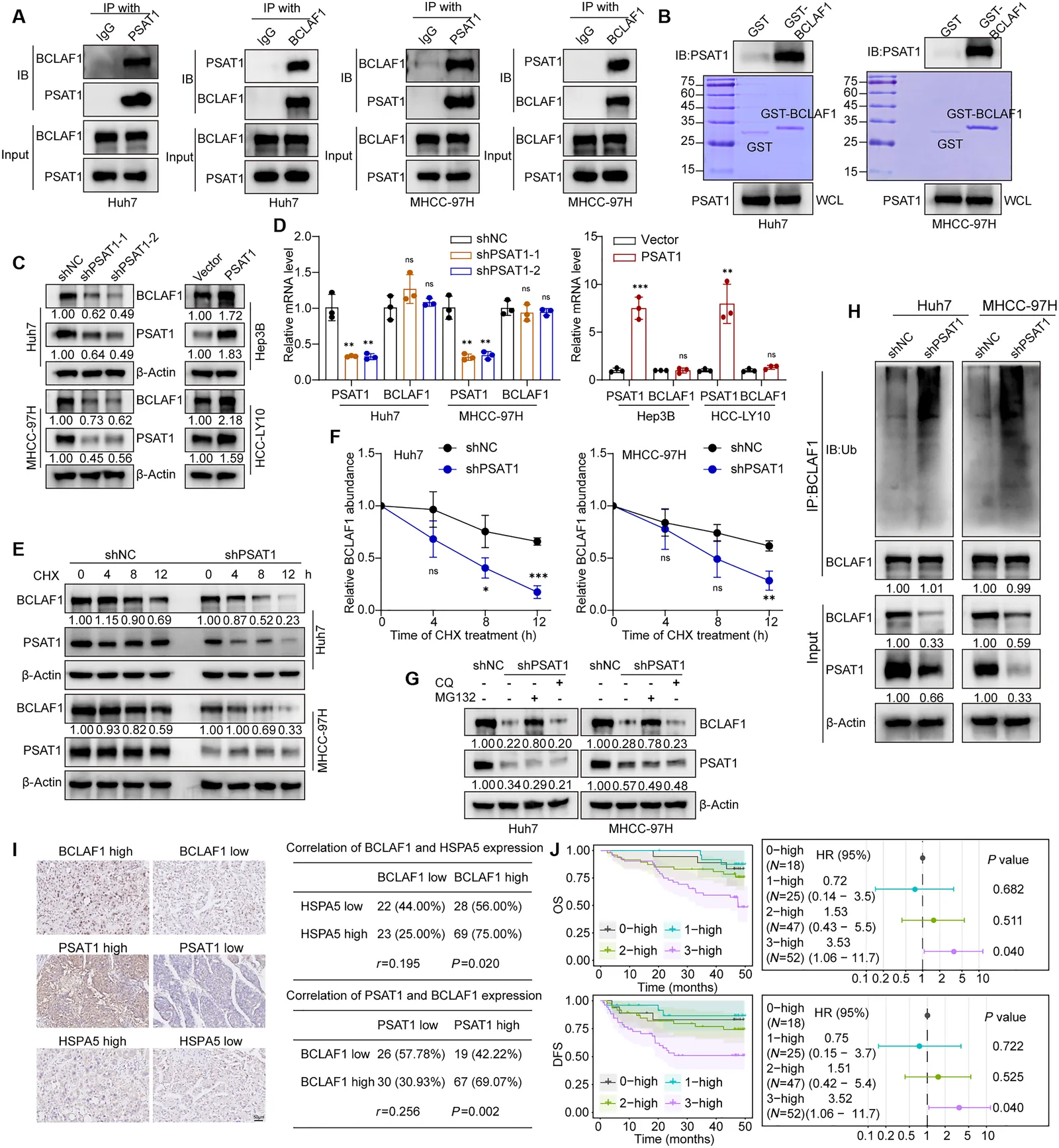
PSAT1 stabilizes BCLAF1 by blocking ubiquitin-proteasome degradation. (**A** and **B**) Co-IP and GST pull-down: direct PSAT1–BCLAF1 interaction in HCC cells. (**C** and **D**) BCLAF1 mRNA/protein levels upon PSAT1 modulation. (**E** and **F**) CHX chase: accelerated BCLAF1 decay after PSAT1 knockdown. (**G**) Proteasome (not lysosome) inhibition rescues BCLAF1 in PSAT1-depleted cells. (**H**) Enhanced BCLAF1 ubiquitination upon PSAT1 knockdown. (**I**) IHC: PSAT1, BCLAF1, and HSPA5 coexpression and correlation in human HCC. (**J**) Kaplan-Meier OS/DFS stratified by combined PSAT1/BCLAF1/HSPA5 expression. Data are presented as the means ± SD. *P < 0.05; **P < 0.01; ***P < 0.001.

Because PSAT1 itself lacks E3 ubiquitin ligase activity, we next sought to identify the E3 ligase responsible for BCLAF1 degradation. Because no candidate E3 ubiquitin ligase was identified in our initial PSAT1 immunoprecipitation-mass spectrometry (IP-MS) dataset, we performed an additional independent PSAT1 IP-MS analysis to broaden the search for potential E3 ligases associated with this complex. Intersection of proteins identified by PSAT1 IP-MS predicted BCLAF1-interacting proteins, and an E3 ligase database (https://e3-ligome-91adc4.gitlab.io/) identified PHRF1 as the only candidate (fig. S7I). Co-IP confirmed the interaction between PHRF1 and BCLAF1, which was enhanced following PSAT1 depletion (fig. S7J). Functionally, PHRF1 knockdown restored BCLAF1 protein abundance in PSAT1-deficient cells and rescued the accelerated degradation of BCLAF1 observed after PSAT1 depletion (fig. S7, K and L). Moreover, PHRF1 silencing markedly reduced the increased BCLAF1 ubiquitination induced by PSAT1 knockdown (fig. S7M). These findings support PHRF1 as a candidate E3 ubiquitin ligase involved in BCLAF1 ubiquitination and degradation following PSAT1 depletion.

Given the established role of HSPA5 in maintaining GPX4 protein stability (*16*), we next examined whether HSPA5 contributes to PSAT1-dependent GPX4 regulation. PSAT1 overexpression increased both HSPA5 and GPX4 protein levels, whereas HSPA5 knockdown markedly attenuated the PSAT1-induced increase in GPX4 in HCC cells (fig. S7N). These findings suggest that HSPA5 may contribute to the regulation of GPX4 protein abundance by PSAT1.

Last, immunohistochemical analysis revealed positive correlations among PSAT1, BCLAF1, and HSPA5 expression in human HCC tissues. Kaplan-Meier analyses further showed that patients with high expression of all three proteins exhibited significantly poorer OS and DFS (Fig. 5, I and J).

### PSAT1 modulating HCC tumorigenesis correlates with tumor-associated macrophage polarization and T_reg_ cell infiltration

Ferroptosis and the tumor microenvironment are closely interconnected (*5*). To explore whether PSAT1 influences immune remodeling, we analyzed intercellular communication using single-cell and bulk transcriptomic datasets. Cell-cell communication analysis revealed that *PSAT1*^+^ malignant hepatocytes exhibited strengthened interactions with myeloid/monocytic cells, neutrophils, and stromal compartments compared with *PSAT1*^−^ malignant cells, suggesting a PSAT1-associated remodeling of the tumor microenvironment (fig. S8, A to C). Across multiple independent HCC cohorts, *PSAT1* expression positively correlated with macrophage infiltration signatures, particularly M2-like macrophages, and negatively correlated with cytotoxic T cell signatures (fig. S8, D to K).

To experimentally validate these findings, we established an immunocompetent orthotopic HCC model using Hepa1-6 cells. PSAT1 overexpression significantly promoted tumor growth and increased Ki67 expression (Fig. 6, A and B, and fig. S9A). Flow cytometry and multiplex immunofluorescence demonstrated increased CD206^+^ macrophages and regulatory T cells (T_reg_ cells), together with reduced CD8^+^ T cell infiltration in PSAT1-overexpressing tumors (Fig. 6, C to F, and fig. S9B). No significant changes were observed in B cells, dendritic cells, myeloid-derived suppressor cells, or natural killer cells (fig. S9C). Similar immune remodeling was observed in a transposon-based AKT/NRAS–driven liver cancer model (Fig. 6, G to L, and fig. S9, D to F), whereas PSAT1 knockdown in a syngeneic Hep53.4 model produced the opposite phenotype, characterized by reduced CD206^+^ macrophages and T_reg_ cells and increased CD8^+^ T cell infiltration (fig. S9, G to N).

**Fig. 6.**
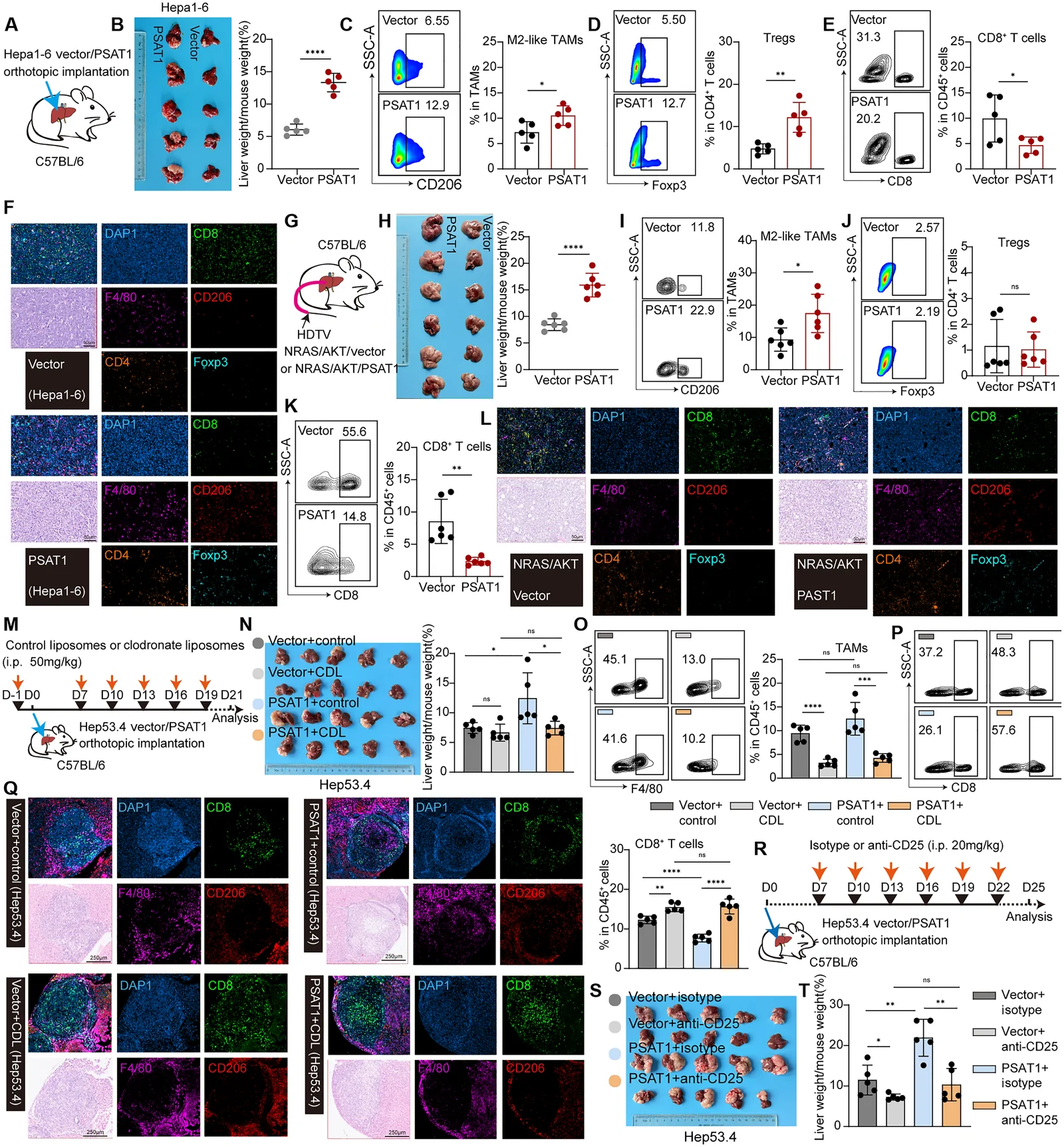
PSAT1 drives HCC tumorigenesis, TAM polarization, and T_reg_ cell infiltration. (**A** and **B**) Orthotopic Hepa1-6 model: PSAT1 overexpression increases tumor burden. (**C** to **E**) Flow cytometry: elevated M2-like TAMs and T_reg_ cells (Tregs) and reduced CD8^+^ T cells in PSAT1-high tumors. (**F**) Multiplex immunofluorescence (IF) confirming immune remodeling. (**G** and **H**) Hydrodynamic tail vein (HDTV) model: PSAT1 overexpression enhances tumor burden. (**I** to **K**) Increased TAMs/T_reg_ cells and decreased CD8^+^ T cells in PSAT1-high HDTV tumors. (**L**) Representative multiplex IF images. (**M** and **N**) Orthotopic Hep53.4 model with clodronate-induced macrophage depletion. (**O** and **P**) Macrophage depletion reduces TAMs and restores CD8^+^ T cells in PSAT1-high tumors. (**Q**) Representative IF images. (**R** to **T**) Orthotopic Hep53.4 model with anti-CD25–mediated T_reg_ cell depletion. Data are presented as the means ± SD. *P < 0.05; **P < 0.01; ***P < 0.001; ****P < 0.0001.

Given that the Hep53.4 model more faithfully recapitulates the immunosuppressive tumor microenvironment of human HCC (*19*), macrophage depletion and anti-CD25 experiments were performed in this orthotopic syngeneic model using PSAT1-overexpressing or control Hep53.4 cells in C57BL/6 mice. Clodronate liposome–mediated macrophage depletion significantly attenuated PSAT1-induced tumor growth and restored CD8^+^ T cell infiltration, as confirmed by flow cytometry and multiplex immunofluorescence (Fig. 6, M to Q, and fig. S9, O and P). Similarly, in vivo T_reg_ cell depletion with an anti-CD25 antibody reduced PSAT1-driven tumor growth and increased intratumoral CD8^+^ T cells (Fig. 6, R to T, and fig. S10, A to D). Collectively, these findings demonstrate that PSAT1 promotes HCC progression by fostering an immunosuppressive microenvironment dependent on macrophage and T_reg_ cell accumulation.

### PSAT1 promotes macrophage M2 polarization and migration through the CCL5-CCR5-STAT6 axis

To investigate how PSAT1 shapes the tumor immune microenvironment, we established in vitro tumor-macrophage coculture systems. THP1-derived macrophages cocultured with PSAT1-knockdown HCC cells exhibited reduced expression of M2-associated markers (*IL10*, *CD163*, *CD206*, and *ARG1*), whereas PSAT1 overexpression induced the opposite effect, accompanied by corresponding changes in CD206 protein expression (Fig. 7, A to D). Chemotactic assays further showed that PSAT1 knockdown impaired, while PSAT1 overexpression enhanced, macrophage recruitment (Fig. 7E and fig. S11, A and B). Similar results were obtained in bone marrow–derived macrophages, indicating a conserved role of PSAT1 in macrophage polarization (fig. S11, C to I). These findings indicate that PSAT1 promotes macrophage recruitment and M2 polarization.

**Fig. 7.**
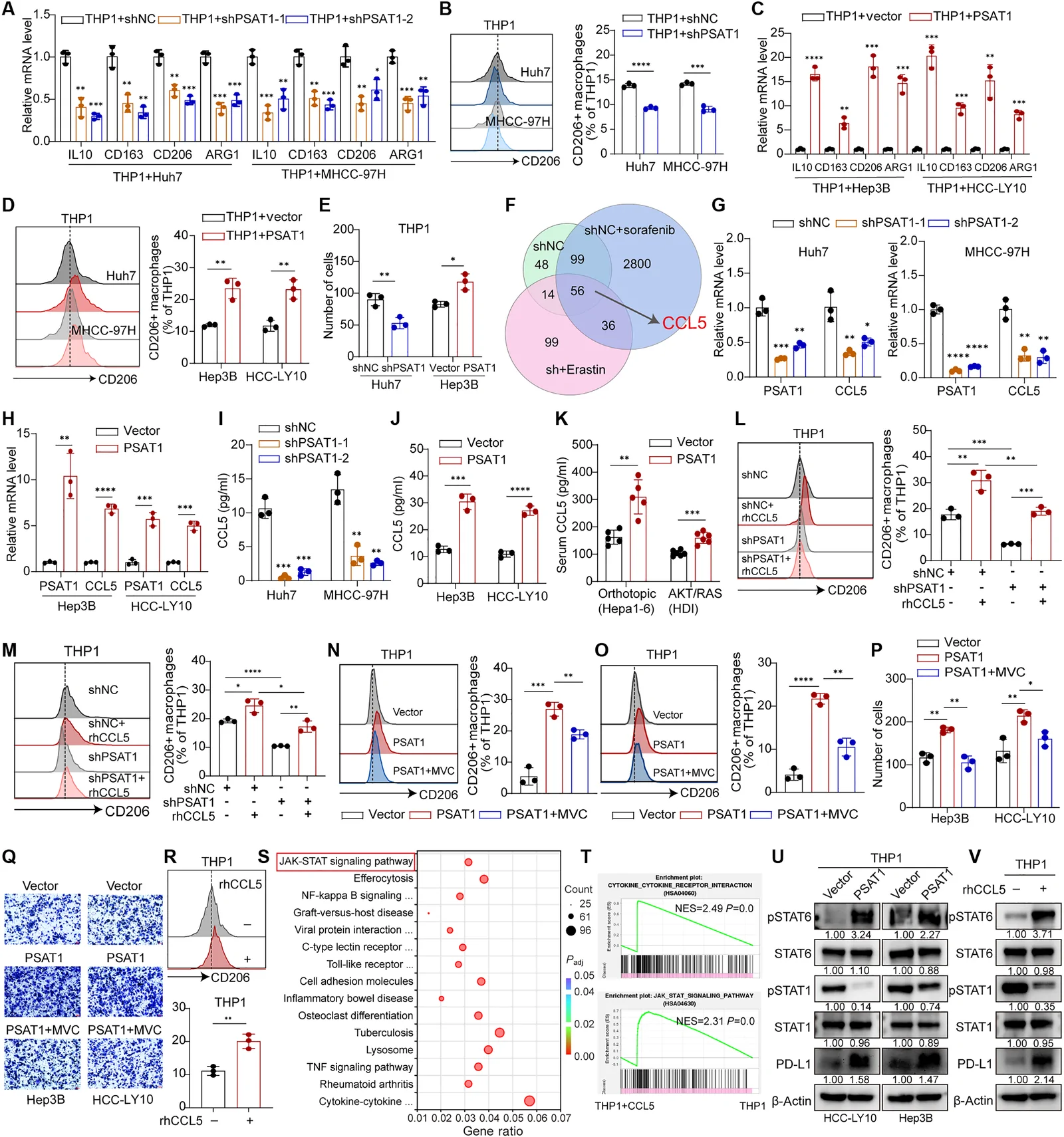
PSAT1 drives M2 polarization and migration via the CCL5-CCR5-STAT6 axis. (**A** to **D**) Coculture: PSAT1 knockdown suppresses, while overexpression enhances, M2 genes and CD206^+^ polarization in THP1 macrophages. (**E**) Transwell assays: PSAT1 modulates macrophage migration. (**F**) Transcriptomics identifies CCL5 as a PSAT1 target. (**G** to **J**) PSAT1 regulates CCL5 mRNA and secretion in HCC cells. (**K**) Serum CCL5 levels in PSAT1-overexpressing mice. (**L** and **M**) Recombinant CCL5 rescues M2 polarization impaired by PSAT1 knockdown. (**N** to **Q**) CCR5 inhibitor (MVC) blocks PSAT1-driven polarization and migration. (**R** to **T**) CCL5 effects on macrophage polarization and JAK-STAT signaling. (**U** and **V**) Immunoblots: STAT6 activation, STAT1 suppression, and PD-L1 up-regulation upon PSAT1/CCL5 stimulation. Data are presented as the means ± SD. *P < 0.05; **P < 0.01; ***P < 0.001; ****P < 0.0001.

RNA-seq analysis following PSAT1 knockdown identified *CCL5* as the only chemokine annotated under MSigDB GO:MF activity gene sets (Fig. 7F and fig. S12A). CCL5 is a prominent cytokine expressed in human various types of cancer cells and was found to be highly correlated with tumor-associated macrophages (TAMs) (*20*, *21*). Furthermore, CCL5/CCR5 signaling has been implicated in recruiting T_reg_ cells and myeloid-derived suppressor cells, further enhancing tumor immunosuppression and influencing macrophage polarization (*22*). PSAT1 depletion reduced, whereas PSAT1 overexpression increased, CCL5 mRNA expression and protein secretion in HCC cells (Fig. 7, G to J). Consistently, PSAT1 overexpression elevated serum CCL5 levels in orthotopic and hydrodynamic HCC models (Fig. 7K). Exogenous CCL5 supplementation rescued the reduction of CD206^+^ macrophages induced by PSAT1 knockdown in coculture systems (Fig. 7, L and M), supporting a functional role for CCL5 in PSAT1-mediated macrophage polarization. Together, these findings support CCL5-CCR5 signaling as a critical mechanism linking PSAT1 to both macrophage recruitment and M2 polarization.

Given that CCR5 is the primary functional receptor for CCL5 and is implicated in TAM-driven tumor progression (*20*, *21*), macrophages were cocultured with PSAT1-overexpressing HCC cells in the presence of the CCR5 antagonist maraviroc (MVC). MVC markedly reduced CD206^+^ macrophage polarization and impaired macrophage recruitment (Fig. 7, N to Q), supporting a requirement for CCL5-CCR5 signaling in PSAT1-driven macrophage reprogramming.

To define downstream signaling, RNA-seq analysis of macrophages treated with recombinant CCL5 revealed enrichment of cytokine-cytokine receptor interaction and JAK (Janus kinase)-STAT (signal transducer and activator of transcription) pathways (Fig. 7, R to T). Mechanistically, coculture with PSAT1-overexpressing HCC cells or CCL5 stimulation selectively increased STAT6 phosphorylation while suppressing STAT1 activation and was accompanied by up-regulation of PD-L1 expression (Fig. 7, U and V). Collectively, these findings demonstrate that PSAT1 promotes macrophage recruitment and M2 polarization through activation of a CCL5-CCR5-STAT6–dominant immunosuppressive program.

### PSAT1-derived CCL5 promotes T_reg_ cell recruitment, survival, and immunosuppressive function

Given the increased T_reg_ cell accumulation associated with PSAT1 expression in HCC, we next investigated whether tumor-derived CCL5 directly regulates T_reg_ cell behavior. T_reg_ cells isolated from the spleens of C57BL/6 mice were seeded in the upper chambers of Transwell inserts, while Hep53.4 cells with PSAT1 overexpression or depletion were placed in the lower chambers. PSAT1-overexpressing Hep53.4 cells significantly enhanced T_reg_ cell migration, whereas this effect was attenuated by the CCR5 antagonist MVC (fig. S12B). Conversely, PSAT1 depletion reduced T_reg_ cell migration, which was partially rescued by exogenous CCL5 supplementation (fig. S12C). Recombinant CCL5 further promoted T_reg_ cell migration in a dose-dependent manner (fig. S12D), supporting a direct chemotactic effect of CCL5 on Treg cells.

We next examined whether CCL5 also influences T_reg_ cell survival. CCL5 treatment significantly reduced T_reg_ cell apoptosis in a dose-dependent manner (fig. S12E), suggesting that enhanced T_reg_ cell survival may additionally contribute to their accumulation.

To determine whether CCL5 affects T_reg_ cell–suppressive function, CCL5-pretreated T_reg_ cells were cocultured with CD8^+^ T cells. Compared with untreated T_reg_ cells, CCL5-treated T_reg_ cells induced greater CD8^+^ T cell apoptosis and more strongly suppressed granzyme B and interferon-γ expression, with stronger effects observed at the higher CCL5 concentration (fig. S12, F to H). Collectively, these findings suggest that PSAT1-induced CCL5 contributes to T_reg_ cell accumulation by promoting both recruitment and survival while simultaneously enhancing T_reg_ cell–mediated suppression of CD8^+^ T cell function.

### PSAT1 promotes CCL5 expression through NRF2

Given that PSAT1 activates the NRF2-ATF4 stress response axis in HCC cells, we examined whether NRF2 or ATF4 regulates CCL5 transcription. Activation of NRF2 by sulforaphane (SFN) significantly increased antioxidant response element–driven luciferase activity, whereas ATF4 overexpression had no effect (fig. S13, A and B). Consistently, NRF2 knockdown reduced *CCL5* mRNA expression, while SFN treatment increased *CCL5* mRNA levels and enhanced CCL5 secretion in HCC cell supernatants (fig. S13, C to F).

JASPAR motif analysis identified four putative antioxidant response elements within the CCL5 promoter [−982 to +3 bp (base pairs); fig. S13G]. Luciferase reporter assays using serial promoter truncations showed that NRF2 activation enhanced *CCL5* promoter activity, with the −248/+4 fragment retaining robust responsiveness, indicating that this proximal region is sufficient for NRF2-dependent transcription (fig. S13, H to K). Mutation of the NRF2 binding site within this region abolished SFN-induced promoter activation (fig. S13L). Consistently, chromatin immunoprecipitation (ChIP) confirmed direct binding of NRF2 to the −248/+4 region of the *CCL5* promoter (fig. S13M). Therefore, these results demonstrate that PSAT1 induces CCL5 expression through direct NRF2-dependent transcriptional activation.

### AOA enhances the efficacy of anti–PD-1 therapy through a ferroptosis-associated mechanism in HCC

To assess the clinical relevance of PSAT1 in immunotherapy response, we analyzed anti–PD-1–treated cohorts and found that *PSAT1* expression was significantly higher in nonresponding patients with HCC (GSE202069) and was also increased following anti–PD-1 therapy in an independent melanoma cohort (GSE145996), indicating an association between PSAT1 up-regulation and resistance to PD-1 blockade (Fig. 8A and fig. S14A).

**Fig. 8.**
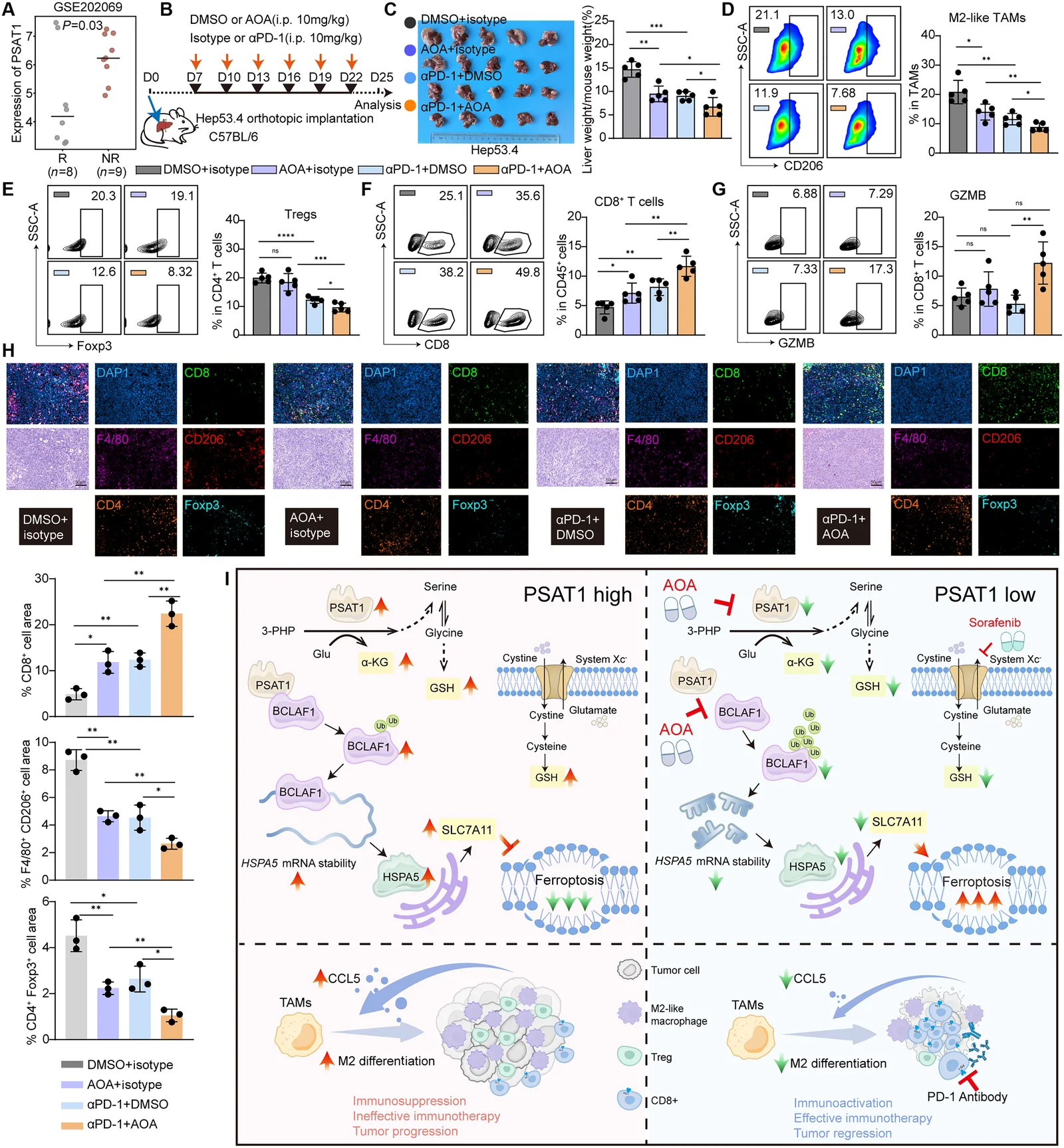
AOA synergizes with anti–PD-1 therapy in HCC. (**A**) *PSAT1* levels in anti–PD-1 responders versus non-responders (GSE202069). (**B**) Orthotopic Hep53.4 model design: vehicle/AOA ± anti–PD-1. (**C**) Representative livers and liver/body weight ratios. (**D** and **E**) Flow cytometry: reduced TAMs and T_reg_ cells with combination therapy. (**F** and **G**) Enhanced CD8^+^ T cell infiltration and cytotoxicity (granzyme B) upon combined treatment. (**H**) Multiplex IF confirming tumor immune remodeling. (**I**) Schematic illustration showing how PSAT1 drives ferroptosis resistance and immune evasion. The schematic was created by the authors using Microsoft PowerPoint (Microsoft Corporation). Data are presented as the means ± SD. *P < 0.05; **P < 0.01; ***P < 0.001; ****P < 0.0001.

We next evaluated whether pharmacological inhibition of PSAT1 enhances anti–PD-1 therapy in vivo. In an orthotopic Hep53.4 HCC model, combined treatment with AOA and anti–PD-1 significantly reduced tumor burden compared with either monotherapy, as indicated by decreased liver weight–to–body weight ratios, without affecting body weight (Fig. 8, B and C, and fig. S14B). Immunohistochemical and flow cytometric analyses showed that combination treatment markedly suppressed tumor proliferation and reshaped the tumor immune microenvironment, characterized by reduced Ki67 positivity, decreased infiltration of M2-like TAMs and T_reg_ cells, and increased accumulation and cytotoxic activity of CD8^+^ T cells (Fig. 8, D to G, and fig. S14, C and D). These immune changes were further confirmed by multiplex immunofluorescence analysis (Fig. 8H).

To determine whether these antitumor and immune-remodeling effects were associated with ferroptosis, we further introduced the ferroptosis inhibitor liproxstatin-1 into the AOA and anti–PD-1 treatment regimen (fig. S14E). Liproxstatin-1 partially reversed the reduction in tumor burden induced by the combination treatment (fig. S14, F and G). Liproxstatin-1 also partially restored M2-like macrophage and T_reg_ cell abundance and reduced the increase in CD8^+^ T cell infiltration observed after AOA and anti–PD-1 treatment (fig. S14, H to M). Multiplex immunofluorescence analysis showed concordant changes in F4/80^+^CD206^+^ macrophages, CD4^+^Foxp3^+^ T_reg_ cells, and CD8^+^ T cells (fig. S14, N to R). Collectively, these findings indicate that AOA enhances the efficacy of anti–PD-1 therapy and remodels the immunosuppressive tumor microenvironment in HCC, while the partial reversal by liproxstatin-1 suggests that ferroptosis contributes to, but may not fully account for, these therapeutic and immune effects.

## DISCUSSION

Despite advances in chemotherapy and immunotherapy, the clinical efficacy of current treatment strategies for HCC remains limited, underscoring the need to identify tumor-intrinsic mechanisms that drive therapeutic resistance (*23*, *24*). Increasing evidence suggests that ferroptosis resistance and immune evasion represent interconnected adaptive programs that enable tumor persistence under therapeutic pressure (*25*, *26*). In this study, we identify PSAT1 as a central regulator that concurrently enforces ferroptosis resistance and promotes immune escape in HCC, thereby linking stress adaptation to immunosuppressive remodeling of the tumor immune microenvironment.

Although PSAT1 is classically defined as a metabolic enzyme in de novo serine biosynthesis (*27*, *28*), our findings indicate that its ferroptosis-protective activity involves both enzymatic and nonenzymatic mechanisms. Reconstitution of PSAT1-depleted cells with the catalytically inactive PSAT1-D100A mutant only partially restored the ferroptosis-related phenotypes, suggesting that PSAT1 enzymatic activity contributes substantially to ferroptosis resistance but is not solely responsible for this effect. We identify a PSAT1-BCLAF1-HSPA5 regulatory axis that operates at the level of RNA stability and translation, providing a mechanistic link between PSAT1 and ferroptosis resistance. Through direct interaction with the RNA binding protein BCLAF1, PSAT1 stabilizes *HSPA5* mRNA and sustains ER proteostasis and antioxidant capacity, enabling tumor cells to tolerate ferroptotic stress. Thus, the PSAT1-BCLAF1-HSPA5 axis represents an additional noncanonical mechanism through which PSAT1 promotes ferroptosis resistance independently, at least in part, of its canonical metabolic activity. These findings highlight posttranscriptional regulation of stress chaperones as an underappreciated mechanism of ferroptosis resistance.

A recent study by Zheng *et al.* (*13*) demonstrated that phosphorylated PSAT1 can directly bind to and stabilize GPX4 independently of its canonical transaminase activity, revealing a noncanonical mechanism of ferroptosis suppression. In our HCC models, GPX4 was not detected in the PSAT1 IP-MS dataset; however, the absence of GPX4 from an unbiased proteomic analysis does not exclude a transient, context-dependent, or stress-induced interaction. Consistent with an enzymatic activity–independent component, the catalytically inactive PSAT1-D100A mutant partially restored GPX4 protein abundance in PSAT1-depleted cells. Our data further suggest an additional mechanism involving HSPA5, as HSPA5 depletion attenuated PSAT1-induced GPX4 accumulation. Given that HSPA5 has been reported to stabilize GPX4 and protect against ferroptosis (*16*), these findings suggest that PSAT1 may regulate GPX4 through multiple, potentially complementary mechanisms, including both direct protein stabilization and an indirect PSAT1-BCLAF1-HSPA5 pathway. Together with the findings of Zheng *et al.* (*13*), our results support a model in which PSAT1-mediated ferroptosis resistance may arise from multiple complementary mechanisms, including canonical metabolic activity, nonenzymatic protein-regulatory functions, and HSPA5-dependent GPX4 stabilization. The relative contribution of these mechanisms may vary according to tumor type and cellular stress context.

RNA binding proteins are emerging as critical regulators of cancer cell plasticity and therapy resistance (*29*, *30*). BCLAF1 has previously been implicated in oncogenic signaling and immune evasion in HCC (*31*, *32*). Our study expands the functional repertoire of BCLAF1 by identifying HSPA5 as a stress-responsive downstream target and positioning BCLAF1 as a key molecular executor through which PSAT1 translates stress adaptation into ferroptosis tolerance.

Beyond tumor cell–intrinsic mechanisms, PSAT1 also plays a pivotal role in shaping the immune landscape of HCC. The hepatic tumor microenvironment is inherently tolerogenic and enriched in immunosuppressive cell populations, particularly TAMs (*33*). We demonstrate that PSAT1 enhances tumor-derived CCL5 secretion, thereby promoting macrophage recruitment and M2-like polarization, increasing T_reg_ cell accumulation, and reducing CD8^+^ T cell infiltration. These findings are consistent with extensive prior studies implicating the CCL5/CCR5 axis in tumor progression across multiple cancers (*34*, *35*), including HCC (*36*). Mechanistically, PSAT1-driven CCL5 activates the CCR5-STAT6 axis in macrophages, reinforcing an immunosuppressive tumor immune microenvironment, consistent with established roles of this pathway in cancer progression. Our findings suggest that the effects of PSAT1 on ferroptosis and immune remodeling are mechanistically distinguishable but functionally interconnected. The PSAT1-CCL5 axis provides a direct mechanism through which tumor cells regulate macrophages and T_reg_ cells and therefore does not necessarily require ferroptosis as an intermediate step. Conversely, ferroptotic susceptibility can profoundly influence antitumor immunity and therapeutic responsiveness. Thus, PSAT1 appears to coordinate two parallel adaptive programs: a tumor cell–intrinsic program that protects malignant cells from ferroptosis and a tumor cell–extrinsic program that promotes an immunosuppressive microenvironment. The convergence of these programs may enable HCC cells to simultaneously withstand oxidative stress and evade immune-mediated elimination.

Given the mechanistic coupling between ferroptosis resistance and immune evasion, rational combination strategies targeting both processes may offer therapeutic benefit (*37*–*39*). Treatment with AOA enhanced ferroptotic susceptibility and, when combined with PD-1 blockade, markedly improved antitumor activity in vivo. Inhibition of ferroptosis with liproxstatin-1 partially reversed the antitumor effect of the AOA and anti–PD-1 combination, indicating that ferroptosis contributes to, but does not fully account for, the therapeutic synergy. These findings suggest that PSAT1-associated metabolic stress and immune checkpoint blockade cooperate through both ferroptosis-dependent and immune-mediated mechanisms.

AOA, however, is a broad-spectrum pyridoxal phosphate–dependent aminotransferase inhibitor and should not be considered a PSAT1-specific inhibitor (*40*, *41*). Although we observed reduced PSAT1 mRNA and protein levels following AOA treatment, suppression of PSAT1 expression is not an expected direct consequence of catalytic inhibition and may instead reflect secondary metabolic or transcriptional feedback responses induced by aminotransferase blockade (*40*). Therefore, the pharmacological effects of AOA cannot be attributed exclusively to PSAT1 inhibition. Rather, our genetic gain- and loss-of-function experiments provide the primary evidence establishing the specific role of PSAT1, whereas the AOA experiments provide a proof of concept that perturbing PSAT1-associated aminotransferase metabolism can enhance ferroptosis and improve the response to immune checkpoint blockade. Development of more selective PSAT1-targeting strategies will be required to determine the translational potential of this vulnerability.

In summary, we identify PSAT1 as a central coordinator of adaptive stress tolerance, ferroptosis resistance, and immune evasion in HCC. PSAT1 protects tumor cells from ferroptosis through both metabolic and noncanonical regulatory mechanisms while independently promoting CCL5-mediated immunosuppressive remodeling of the tumor microenvironment. These two functions ultimately converge to facilitate tumor survival and resistance to immunotherapy. Targeting the PSAT1-associated metabolic and stress-response network may therefore represent a promising strategy to simultaneously enhance ferroptotic vulnerability and antitumor immunity in HCC (Fig. 8I).

## MATERIALS AND METHODS

### Cell lines and cell culture

Huh7 cells were obtained from Riken Cell Bank (Tsukuba, Japan). Human embryonic kidney (HEK) 293T and Hepa1-6 cell lines were purchased from the American Type Culture Collection (Manassas, VA). The mouse cell lines Hep53.4 originally purchased from CLS-Cell Lines Service (Eppelheim, Germany) were provided by C. Wang (Shanghai Cancer Institute). The MHCC-LM3, Hep3B, and MHCC-97H cell lines were obtained from the Liver Cancer Institute, Zhongshan Hospital of Fudan University (Shanghai, China). The HCC-LY10 cell line was established in our laboratory (*42*). Cells were cultured in Dulbecco’s modified Eagle’s medium (Gibco) supplemented with 1% penicillin/streptomycin and 10% fetal bovine serum (Gibco) at 37°C under 5% CO_2_. All of the cell lines were authenticated and characterized by the suppliers. Cells were used within 6 months of resuscitation. These cell lines were mycoplasma-free and routinely authenticated by quality examinations of morphology and growth profile.

### Lentivirus production and cell transduction

The PSAT1 lentiviral overexpression plasmid and short hairpin RNA (shRNA) plasmid were supplied by the CCSB-Broad Lentiviral Expression Library and Human TRC shRNA Library. The NRF2 knockdown plasmid was maintained in our laboratory (*43*). The mouse PSAT1 lentiviral overexpression plasmid was constructed in our laboratory. BCLAF1 plasmids were purchased from Miaoling Plasmid Library (Wuhan, China). Sequences of primers and target sequences are listed in table S1. Viral packaging was performed in HEK-293T cells after cotransfection of the PSAT1 overexpression or shRNA plasmid with the packaging plasmid psPAX2 and the envelope plasmid pMD2.G (Addgene) using Lipofectamine 2000 (Invitrogen). The viruses were harvested 72 hours after transfection, and the viral titers were determined. HCC cells were transduced with 1 × 10^6^ recombinant lentivirus–transducing units in the presence of polybrene (6 μg/ml; Sigma-Aldrich, US).

### Mouse models

For the hydrodynamic transfection HCC model, plasmid DNA suspended in 2 ml of saline (0.9% NaCl) was injected via tail vein into C57BL/6 male mice within 5 to 7 s. The plasmid was used in the dosage of 20 μg of pT3-myr-AKT-HA and 20 μg of pT/Caggs-NRASV12 and pWPXL or pWPXL-PSAT1 together with 5 μg of pCMV (CAT)T7-SB100 transposase per mouse. All mice were euthanized by carbon dioxide overdose at the end of the study.

For the in vivo isograft experiment, 2 × 10^6^ Hepa1-6 cells or 1 × 10^6^ Hep53.4 cells were mixed in 40 μl of a 1:1 solution of serum-free Dulbecco’s modified Eagle’s medium and Matrigel (BD Biosciences, MA) and injected into the left hepatic lobes of the 6-week-old male C57BL/6J through an 8-mm transverse incision in the upper abdomen using a microsyringe. For anti–PD-1 treatment experiment, animals were randomly assigned to the four groups that were administered 100 μg of anti–PD-1 (BE0146, Bio X Cell, US) or immunoglobulin (IgG) isotype control via intraperitoneal injection every other day. For anti-CD25 treatment experiment, animals were randomly assigned to the four groups that were administered 100 μg of anti-CD25 (BE0012, Bio X Cell, US) or IgG isotype control via intraperitoneal injection every other day.

For the in vivo xenograft experiment, 6-week-old male C57BL/6J mice were subcutaneously injected with 2 × 10^6^ cells. When the tumor volume was ∼100 mm^3^, the mice were randomized into four groups. The mice were treated with sorafenib (10 mg/kg, once every other day) via intraperitoneal injection. The tumor dimensions were measured with Vernier calipers every 3 days, and the tumor volume was calculated as follows: tumor volume = (length × width^2^)/2.

Tumor fragments from patients with HCC (25 to 40 mm^3^) were initially implanted subcutaneously into NCG mice (6 to 8 weeks old) to establish the patient-derived xenograft model. After multiple passages (F_3_ generation) to ensure stable engraftment, the tumors were harvested and inoculated into nude mice. When the tumor volume approximated 100 mm^3^, patient-derived xenograft model mice were randomized into experimental and control groups.

The mice were closely monitored over a period of 6 weeks. At the end of the experiment, the mice were euthanized, and the tissues were harvested. The harvested tissues were fixed in neutral formalin buffered with phosphate for at least 72 hours. All animal experiments were approved by the Laboratory Animal Ethics Committee of the Renji Hospital, Shanghai Jiao Tong University School of Medicine (approval number: RT2022-122u). All methods are reported in accordance with ARRIVE (Animal Research: Reporting of In Vivo Experiments) guidelines for the reporting of animal experiments.

### Immunohistochemistry (IHC)

IHC assays were conducted as reported previously (*44*). A total of 142 HCC tissues were obtained from the Zhongshan Hospital of Fudan University (Shanghai, China). All samples were obtained with informed consent. The study was approved by the Ethics Committee of Renji Hospital, Shanghai Jiao Tong University School of Medicine (KY2023-084-B). Briefly, the sections were deparaffinized with xylene and rehydrated before being heated to just below boiling temperature at a subboiling temperature in sodium citrate buffer (pH 6.0) for 20 min in a microwave oven for antigen retrieval. After being washed with phosphate-buffered saline (PBS) for three times, the samples were incubated with 3% hydrogen peroxide for 10 min to block endogenous peroxidase activity. The sections were then incubated overnight with primary antibodies at 4°C. After being rinsed with PBS, the sections were incubated with a peroxidase-conjugated secondary antibody at 37°C for 30 min and then incubated with diaminobenzidine solution. Last, the nuclei were counterstained with Mayer’s hematoxylin. The intensity of the staining was evaluated by two independent investigators blinded to the clinical pathological data of patients using the following criteria: 0, negative; 1, low; 2, medium; 3, high. The extent of staining was scored as follows: 0, 0% stained; 1, 1 to 25% stained; 2, 26 to 50% stained; 3, 51 to 100% stained. Samples with a final staining score ≤4 were considered to exhibit low expression, and those with a score >4 were considered to exhibit high expression. Information of the antibodies is listed in table S3.

### RIP assay

RIP assay was conducted using the RNA Binding Protein Immunoprecipitation (RIP) Kit (Absin, China) according to the manufacturer’s protocol. Cells were lysed in complete RIP lysis buffer. Briefly, 100 μl of cell lysates was set aside for input control. To obtain the antibody-bead complex, pretreated magnetic protein A + G beads were co-incubated with specific antibodies or control anti-IgG and rotated overnight at 4°C. RNA complexes bound to beads were washed, and RNA was extracted using TRIzol reagent (Invitrogen, CA) and analyzed by quantitative reverse transcription polymerase chain reaction.

### In vitro translation assay

The in vitro translation assay was performed using a rabbit reticulocyte lysate system according to the manufacturer’s instructions. Briefly, total RNA was extracted from Huh7 or MHCC-97H cells, and mRNA was subsequently purified using an mRNA isolation kit. Purified mRNA (1 μg) was added to 35 μl of rabbit reticulocyte lysate supplemented with a methionine-free amino acid mixture, 40 U RNasin, Transcend biotin-lysyl-tRNA, and 4 μg of purified recombinant human BCLAF1-GST protein, as indicated. The reaction mixtures were incubated at 30°C for 1.5 hours. Translation products were then separated by SDS–polyacrylamide gel electrophoresis and transferred onto polyvinylidene difluoride membranes. Newly synthesized biotin-labeled proteins were detected using horseradish peroxidase–conjugated streptavidin followed by chemiluminescent detection. The signal corresponding to HSPA5 was quantified relative to the indicated control group.

### RNA pull-down assay and liquid chromatography–tandem mass spectrometry analysis

RNA pull-down assay was performed using the Pierce Magnetic RNA–Protein Kit (Thermo Fisher Scientific, US) according to the manufacturer’s instructions. Briefly, a biotinylated HSPA5 probe or a negative control probe (GenePharma, China) was incubated with streptavidin-coated magnetic beads for 4 hours. Subsequently, cell lysates were incubated with the probe-bead complexes for 12 hours at 4°C. After washing, the magnetic beads were analyzed for potential binding proteins by silver staining and mass spectrometry.

### Co-IP assay

The cells were harvested in radioimmunoprecipitation assay (Upstate, Biotechnology) lysis buffer containing protease inhibitors for 40 min on ice and centrifuged at 12,000*g* for 10 min. Total cell lysates were incubated with 2 μg of primary antibody or control IgG at 4°C overnight. Then, 20 μl of protein A/G agarose beads was added, followed by further incubation for 2 hours at 4°C. The immunoprecipitates were collected by centrifugation and washed with PBS. The samples were denaturized for Western blotting.

### ChIP assay

ChIP assays were performed using a ChIP assay kit (Upstate Biotechnology), as described previously (*45*). Briefly, cells were cross-linked by the addition of 1% formaldehyde for 10 min. The cell lysates were sonicated to shear the DNA to fragments of 500 to 1000 bp. A rabbit anti-NRF2 antibody or rabbit IgG was used to immunoprecipitate DNA-containing complexes. Then, the chromatin was incubated with protein A/G beads for 2 hours. After reverse cross-linking of the protein/DNA complexes to free DNA, quantitative reverse transcription polymerase chain reaction was performed to detect the NRF2 binding site in the CCL5 promoter region. The primer sequences are listed in table S2.

### Statistical analysis

All data were presented as the means ± standard deviation (SD). A one-way analysis of variance (ANOVA) was used for comparisons among multiple groups, while Student’s *t* test was used for two-group comparisons. Kaplan-Meier survival curves were generated, and multivariate survival analysis was conducted using the Cox proportional hazards model. Pearson correlation analysis was used for the analysis of the correlations. *P* < 0.05 was considered to indicate statistical significance.

## References

[1] A. Vogel, T. Meyer, G. Sapisochin, R. Salem, A. Saborowski, Hepatocellular carcinoma. Lancet 400, 1345–1362 (2022). 10.1016/S0140-6736(22)01200-4 36084663

[2] G. Morad, B. A. Helmink, P. Sharma, J. A. Wargo, Hallmarks of response, resistance, and toxicity to immune checkpoint blockade. Cell 184, 5309–5337 (2021). 10.1016/j.cell.2021.09.020 PMC8767569 34624224

[3] J. M. Llovet, F. Castet, M. Heikenwalder, M. K. Maini, V. Mazzaferro, D. J. Pinato, E. Pikarsky, A. X. Zhu, R. S. Finn, Immunotherapies for hepatocellular carcinoma. Nat. Rev. Clin. Oncol. 19, 151–172 (2022). 10.1038/s41571-021-00573-2 34764464

[4] X. Jiang, B. R. Stockwell, M. Conrad, Ferroptosis: Mechanisms, biology and role in disease. Nat. Rev. Mol. Cell Biol. 22, 266–282 (2021). 10.1038/s41580-020-00324-8 PMC8142022 33495651

[5] G. Lei, L. Zhuang, B. Gan, The roles of ferroptosis in cancer: Tumor suppression, tumor microenvironment, and therapeutic interventions. Cancer Cell 42, 513–534 (2024). 10.1016/j.ccell.2024.03.011 38593779

[6] L. Galluzzi, T. Yamazaki, G. Kroemer, Linking cellular stress responses to systemic homeostasis. Nat. Rev. Mol. Cell Biol. 19, 731–745 (2018). 10.1038/s41580-018-0068-0 30305710

[7] X. Chen, J. R. Cubillos-Ruiz, Endoplasmic reticulum stress signals in the tumour and its microenvironment. Nat. Rev. Cancer 21, 71–88 (2021). 10.1038/s41568-020-00312-2 PMC7927882 33214692

[8] C. L. Kriss, J. A. Pinilla-Ibarz, A. W. Mailloux, J. J. Powers, C. H. Tang, C. W. Kang, N. Zanesi, P. K. Epling-Burnette, E. M. Sotomayor, C. M. Croce, J. R. Del Valle, C. C. Hu, Overexpression of TCL1 activates the endoplasmic reticulum stress response: A novel mechanism of leukemic progression in mice. Blood 120, 1027–1038 (2012). 10.1182/blood-2011-11-394346 PMC3680046 22692508

[9] J. R. Cubillos-Ruiz, S. E. Bettigole, L. H. Glimcher, Tumorigenic and immunosuppressive effects of endoplasmic reticulum stress in cancer. Cell 168, 692–706 (2017). 10.1016/j.cell.2016.12.004 PMC5333759 28187289

[10] J. Jiang, H. N. Chen, P. Jin, L. Zhou, L. Peng, Z. Huang, S. Qin, B. Li, H. Ming, M. Luo, N. Xie, W. Gao, E. C. Nice, Q. Yu, C. Huang, Targeting PSAT1 to mitigate metastasis in tumors with p53-72Pro variant. Signal Transduct. Target. Ther. 8, 65 (2023). 10.1038/s41392-022-01266-7 PMC9929071 36788227

[11] Y. C. Chan, Y. C. Chang, H. H. Chuang, Y. C. Yang, Y. F. Lin, M. S. Huang, M. Hsiao, C. J. Yang, K. T. Hua, Overexpression of PSAT1 promotes metastasis of lung adenocarcinoma by suppressing the IRF1-IFNgamma axis. Oncogene 39, 2509–2522 (2020). 10.1038/s41388-020-1160-4 31988456

[12] M. Y. Luo, Y. Zhou, W. M. Gu, C. Wang, N. X. Shen, J. K. Dong, H. M. Lei, Y. B. Tang, Q. Liang, J. H. Zou, L. Xu, P. Ma, G. Zhuang, L. Bi, L. Xu, L. Zhu, H. Z. Chen, Y. Shen, Metabolic and nonmetabolic functions of PSAT1 coordinate signaling cascades to confer EGFR inhibitor resistance and drive progression in lung adenocarcinoma. Cancer Res. 82, 3516–3531 (2022). 10.1158/0008-5472.CAN-21-4074 36193649

[13] P. Zheng, Z. Hu, Y. Shen, L. Gu, Y. Ouyang, Y. Duan, G. Ji, B. Dong, Y. Lin, T. Wen, Q. Tian, Y. Hou, Q. Zhou, X. Sun, X. Chen, K. L. Wang, S. Luo, S. Wu, Y. Sun, M. Li, L. Xiao, Q. Wu, Y. Meng, G. Liu, Z. Wang, X. Bai, S. Duan, Y. Ding, Y. Bi, Y. Wang, G. Li, X. Liu, Z. Lu, X. Wu, Z. Tang, D. Xu, PSAT1 impairs ferroptosis and reduces immunotherapy efficacy via GPX4 hydroxylation. Nat. Chem. Biol. 21, 1420–1432 (2025). 10.1038/s41589-025-01887-3 40281343

[14] R. Acuna-Hidalgo, D. Schanze, A. Kariminejad, A. Nordgren, M. H. Kariminejad, P. Conner, G. Grigelioniene, D. Nilsson, M. Nordenskjold, A. Wedell, C. Freyer, A. Wredenberg, D. Wieczorek, G. Gillessen-Kaesbach, H. Kayserili, N. Elcioglu, S. Ghaderi-Sohi, P. Goodarzi, H. Setayesh, M. van de Vorst, M. Steehouwer, R. Pfundt, B. Krabichler, C. Curry, M. G. MacKenzie, K. M. Boycott, C. Gilissen, A. R. Janecke, A. Hoischen, M. Zenker, Neu-Laxova syndrome is a heterogeneous metabolic disorder caused by defects in enzymes of the L-serine biosynthesis pathway. Am. J. Hum. Genet. 95, 285–293 (2014). 10.1016/j.ajhg.2014.07.012 PMC4157144 25152457

[15] I. M. Ibrahim, D. H. Abdelmalek, A. A. Elfiky, GRP78: A cell’s response to stress. Life Sci. 226, 156–163 (2019). 10.1016/j.lfs.2019.04.022 PMC7094232 30978349

[16] S. Zhu, Q. Zhang, X. Sun, H. J. Zeh III, M. T. Lotze, R. Kang, D. Tang, HSPA5 regulates ferroptotic cell death in cancer cells. Cancer Res. 77, 2064–2077 (2017). 10.1158/0008-5472.CAN-16-1979 PMC5392369 28130223

[17] X. Pan, Y. Fang, X. Li, Y. Yang, H. B. Shen, RBPsuite: RNA-protein binding sites prediction suite based on deep learning. BMC Genomics 21, 884 (2020). 10.1186/s12864-020-07291-6 PMC7724624 33297946

[18] W. Li, X. Deng, J. Chen, RNA-binding proteins in regulating mRNA stability and translation: Roles and mechanisms in cancer. Semin. Cancer Biol. 86, 664–677 (2022). 10.1016/j.semcancer.2022.03.025 PMC9526761 35381329

[19] D. J. Zabransky, L. Danilova, J. M. Leatherman, T. Y. Lopez-Vidal, J. Sanchez, S. Charmsaz, N. E. Gross, S. Shin, X. Yuan, A. Hernandez, H. Yang, S. Xavier, D. Shu, A. Saeed, K. Munjal, Z. Kamdar, L. T. Kagohara, E. M. Jaffee, M. Yarchoan, W. J. Ho, Profiling of syngeneic mouse HCC tumor models as a framework to understand anti-PD-1 sensitive tumor microenvironments. Hepatology 77, 1566–1579 (2023). 10.1002/hep.32707 PMC9905363 35941803

[20] A. Walens, A. V. DiMarco, R. Lupo, B. R. Kroger, J. S. Damrauer, J. V. Alvarez, CCL5 promotes breast cancer recurrence through macrophage recruitment in residual tumors. eLife 8, e43653 (2019). 10.7554/eLife.43653 PMC6478432 30990165

[21] A. Mohs, N. Kuttkat, J. Reissing, H. W. Zimmermann, R. Sonntag, A. Proudfoot, S. A. Youssef, A. de Bruin, F. J. Cubero, C. Trautwein, Functional role of CCL5/RANTES for HCC progression during chronic liver disease. J. Hepatol. 66, 743–753 (2017). 10.1016/j.jhep.2016.12.011 28011329

[22] B. L. Schlechter, J. Stebbing, CCR5 and CCL5 in metastatic colorectal cancer. J. Immunother. Cancer 12, e008722 (2024). 10.1136/jitc-2023-008722 PMC11086517 38719543

[23] R. S. Finn, S. Qin, M. Ikeda, P. R. Galle, M. Ducreux, T. Y. Kim, M. Kudo, V. Breder, P. Merle, A. O. Kaseb, D. Li, W. Verret, D. Z. Xu, S. Hernandez, J. Liu, C. Huang, S. Mulla, Y. Wang, H. Y. Lim, A. X. Zhu, A. L. Cheng, IMbrave150 Investogators, Atezolizumab plus bevacizumab in unresectable hepatocellular carcinoma. N. Engl. J. Med. 382, 1894–1905 (2020). 10.1056/NEJMoa1915745 32402160

[24] S. Cappuyns, V. Corbett, M. Yarchoan, R. S. Finn, J. M. Llovet, Critical appraisal of guideline recommendations on systemic therapies for advanced hepatocellular carcinoma: A review. JAMA Oncol. 10, 395–404 (2024). 10.1001/jamaoncol.2023.2677 PMC10837331 37535375

[25] W. Wang, M. Green, J. E. Choi, M. Gijon, P. D. Kennedy, J. K. Johnson, P. Liao, X. Lang, I. Kryczek, A. Sell, H. Xia, J. Zhou, G. Li, J. Li, W. Li, S. Wei, L. Vatan, H. Zhang, W. Szeliga, W. Gu, R. Liu, T. S. Lawrence, C. Lamb, Y. Tanno, M. Cieslik, E. Stone, G. Georgiou, T. A. Chan, A. Chinnaiyan, W. Zou, CD8^+^ T cells regulate tumour ferroptosis during cancer immunotherapy. Nature 569, 270–274 (2019). 10.1038/s41586-019-1170-y PMC6533917 31043744

[26] H. N. Bell, B. R. Stockwell, W. Zou, Ironing out the role of ferroptosis in immunity. Immunity 57, 941–956 (2024). 10.1016/j.immuni.2024.03.019 PMC11101142 38749397

[27] V. Ciuffoli, X. Feng, K. Jiang, N. Acevedo-Luna, K. D. Ko, A. H. J. Wang, G. Riparini, M. Khateb, B. Glancy, S. Dell’Orso, V. Sartorelli, Psat1-generated alpha-ketoglutarate and glutamine promote muscle stem cell activation and regeneration. Genes Dev. 38, 151–167 (2024). 10.1101/gad.351428.123 PMC10982694 38453480

[28] Y. Qiu, O. T. Stamatatos, Q. Hu, J. Ruiter Swain, S. Russo, A. Sann, A. S. H. Costa, S. Violante, D. L. Spector, J. R. Cross, M. J. Lukey, The unique catalytic properties of PSAT1 mediate metabolic adaptation to glutamine blockade. Nat. Metab. 6, 1529–1548 (2024). 10.1038/s42255-024-01104-w PMC11490312 39192144

[29] B. M. Lunde, C. Moore, G. Varani, RNA-binding proteins: Modular design for efficient function. Nat. Rev. Mol. Cell Biol. 8, 479–490 (2007). 10.1038/nrm2178 PMC5507177 17473849

[30] M. W. Hentze, A. Castello, T. Schwarzl, T. Preiss, A brave new world of RNA-binding proteins. Nat. Rev. Mol. Cell Biol. 19, 327–341 (2018). 10.1038/nrm.2017.130 29339797

[31] X. Zhou, Y. Wen, Y. Tian, M. He, X. Ke, Z. Huang, Y. He, L. Liu, A. Scharf, M. Lu, G. Zhang, Y. Deng, Y. Yan, M. P. Mayer, X. Chen, F. Zou, Heat shock protein 90α-dependent B-cell-2-associated transcription factor 1 promotes hepatocellular carcinoma proliferation by regulating MYC proto-oncogene c-MYC mRNA stability. Hepatology 69, 1564–1581 (2019). 10.1002/hep.30172 PMC6586158 30015413

[32] Z. Yu, X. Wu, J. Zhu, H. Yan, Y. Li, H. Zhang, Y. Zhong, M. Lin, G. Ye, X. Li, J. Jin, K. Li, J. Wang, H. Zhuang, T. Lin, J. He, C. Lu, Z. Xu, X. Zhang, H. Li, X. Jin, BCLAF1 binds SPOP to stabilize PD-L1 and promotes the development and immune escape of hepatocellular carcinoma. Cell. Mol. Life Sci. 81, 82 (2024). 10.1007/s00018-024-05144-z PMC10858942 38340178

[33] C. Zheng, L. Zheng, J. K. Yoo, H. Guo, Y. Zhang, X. Guo, B. Kang, R. Hu, J. Y. Huang, Q. Zhang, Z. Liu, M. Dong, X. Hu, W. Ouyang, J. Peng, Z. Zhang, Landscape of infiltrating T cells in liver cancer revealed by single-cell sequencing. Cell 169, 1342–1356.e16 (2017). 10.1016/j.cell.2017.05.035 28622514

[34] B. Liu, W. Zhang, Y. Ji, J. Wu, R. Su, X. Liu, A. Li, K. Shen, X. Chai, H. Wu, Z. Ma, C. Hu, Z. Jiang, L. Dong, Y. Zhu, B. Dong, W. Xue, J. Pan, Q. Wang, Targeting the CCL5/CCR5 axis in tumor-stromal crosstalk to overcome cisplatin resistance in neuroendocrine prostate cancer. J. Exp. Clin. Cancer Res. 44, 296 (2025). 10.1186/s13046-025-03552-y PMC12570517 41152972

[35] N. Halama, I. Zoernig, A. Berthel, C. Kahlert, F. Klupp, M. Suarez-Carmona, T. Suetterlin, K. Brand, J. Krauss, F. Lasitschka, T. Lerchl, C. Luckner-Minden, A. Ulrich, M. Koch, J. Weitz, M. Schneider, M. W. Buechler, L. Zitvogel, T. Herrmann, A. Benner, C. Kunz, S. Luecke, C. Springfeld, N. Grabe, C. S. Falk, D. Jaeger, Tumoral immune cell exploitation in colorectal cancer metastases can be targeted effectively by anti-CCR5 therapy in cancer patients. Cancer Cell 29, 587–601 (2016). 10.1016/j.ccell.2016.03.005 27070705

[36] S. K. Singh, M. K. Mishra, B. M. Rivers, J. B. Gordetsky, S. Bae, R. Singh, Biological and clinical significance of the CCR5/CCL5 axis in hepatocellular carcinoma. Cancers 12, 883 (2020). 10.3390/cancers12040883 PMC7226629 32260550

[37] F. Fan, P. Liu, R. Bao, J. Chen, M. Zhou, Z. Mo, Y. Ma, H. Liu, Y. Zhou, X. Cai, C. Qian, X. Liu, A dual PI3K/HDAC inhibitor induces immunogenic ferroptosis to potentiate cancer immune checkpoint therapy. Cancer Res. 81, 6233–6245 (2021). 10.1158/0008-5472.CAN-21-1547 34711611

[38] X. Hao, Z. Zheng, H. Liu, Y. Zhang, J. Kang, X. Kong, D. Rong, G. Sun, G. Sun, L. Liu, H. Yu, W. Tang, X. Wang, Inhibition of APOC1 promotes the transformation of M2 into M1 macrophages via the ferroptosis pathway and enhances anti-PD1 immunotherapy in hepatocellular carcinoma based on single-cell RNA sequencing. Redox Biol. 56, 102463 (2022). 10.1016/j.redox.2022.102463 PMC9482117 36108528

[39] P. Liu, C. Lin, Z. Liu, C. Zhu, Z. Lin, D. Xu, J. Chen, Q. Huang, C. Y. Li, L. Hou, J. A. Pan, X. Liu, Inhibition of ALG3 stimulates cancer cell immunogenic ferroptosis to potentiate immunotherapy. Cell. Mol. Life Sci. 79, 352 (2022). 10.1007/s00018-022-04365-4 PMC11072400 35676564

[40] P. Korangath, W. W. Teo, H. Sadik, L. Han, N. Mori, C. M. Huijts, F. Wildes, S. Bharti, Z. Zhang, C. A. Santa-Maria, H. Tsai, C. V. Dang, V. Stearns, Z. M. Bhujwalla, S. Sukumar, Targeting glutamine metabolism in breast cancer with aminooxyacetate. Clin. Cancer Res. 21, 3263–3273 (2015). 10.1158/1078-0432.CCR-14-1200 PMC4696069 25813021

[41] C. S. Yang, E. Stampouloglou, N. M. Kingston, L. Zhang, S. Monti, X. Varelas, Glutamine-utilizing transaminases are a metabolic vulnerability of TAZ/YAP-activated cancer cells. EMBO Rep. 19, e43577 (2018). 10.15252/embr.201643577 PMC5989844 29661856

[42] H. Tian, C. Ge, H. Li, F. Zhao, H. Hou, T. Chen, G. Jiang, H. Xie, Y. Cui, M. Yao, J. Li, Ribonucleotide reductase M2B inhibits cell migration and spreading by early growth response protein 1-mediated phosphatase and tensin homolog/Akt1 pathway in hepatocellular carcinoma. Hepatology 59, 1459–1470 (2014). 10.1002/hep.26929 24214128

[43] J. Li, C. Zhang, Q. Zhou, Q. Long, J. Chen, L. Meng, W. Tian, Y. Yang, C. Ge, Y. Su, X. D. Long, J. Wu, H. Tian, ALDH1L2 drives HCC progression through TAM polarization. JHEP Rep. 7, 101217 (2025). 10.1016/j.jhepr.2024.101217 PMC11648791 39687603

[44] W. Tian, Y. Yang, L. Meng, C. Ge, Y. Liu, C. Zhang, Z. Huang, C. Zhang, H. Tian, GCDH acetylation orchestrates DNA damage response and autophagy via mitochondrial ROS to suppress hepatocellular carcinoma progression. Research 8, 0862 (2025). 10.34133/research.0862 PMC12395558 40896397

[45] Z. Jiang, Q. Zhou, C. Ge, J. Yang, H. Li, T. Chen, H. Xie, Y. Cui, M. Shao, J. Li, H. Tian, Rpn10 promotes tumor progression by regulating hypoxia-inducible factor 1 alpha through the PTEN/Akt signaling pathway in hepatocellular carcinoma. Cancer Lett. 447, 1–11 (2019). 10.1016/j.canlet.2019.01.020 30673593

