# Supplementary material for: PSAT1 integrates ferroptosis resistance and immune evasion to drive hepatocellular carcinoma progression
Source: Sci Adv. 2026 Oct 28;12(44):eaei7877. doi: 10.1126/sciadv.aei7877 (PMC13715705; doi:10.1126/sciadv.aei7877)
Supplement: Supplementary file 1 [file sciadv.aei7877_sm.pdf]

Science Advances
AAAS

Supplementary Materials for

PSAT1 integrates ferroptosis resistance and immune evasion to drive hepatocellular carcinoma progression

Chi Zhang *et al.*

Corresponding author: Hua Tian, htian@shsci.org

Sci. Adv. **12**, eaei7877 (2026)
DOI: 10.1126/sciadv.aei7877

This PDF file includes:

Supplementary Materials and Methods
Figs. S1 to S15
Tables S1 to S3

Supplementary Materials and Methods

Quantitative real-time RT–PCR (qRT–PCR)

Total RNA was isolated from cells using TRIzol reagent (Invitrogen), and cDNA was generated using a reverse transcription kit (TaKaRa). Real-time fluorescence quantitative PCR was performed with SYBR® *Premix Ex Taq* (Takara, Dalian, China) using an ABI Prism 7500 System (Applied Biosystems, Carlsbad, CA, USA). Normalize the threshold period (Ct) of the target gene to the threshold of GAPDH and use $2^{-\Delta\Delta Ct}$ to calculate the relative expression level of target genes. Sequences of primers are listed in Supplementary Table S2.

Western blotting

The protein concentration was determined using a BCA assay (Beyotime, Shanghai). Proteins in whole cell lysates were separated by SDS–polyacrylamide gel electrophoresis and transferred onto PVDF membranes (Millipore, USA). After blocking with 5% non-fat milk, the membrane was incubated overnight at 4 ºC with the primary antibody. Then HRP-conjugated secondary antibodies were incubated for 1 hour at room temperature and the signal was detected with enhanced chemiluminescence reagent (Pierce, Rockford, IL, USA). Information of the antibodies is listed in Supplementary Table S3.

Cell proliferation and colony formation assays

Cell proliferation was measured by the Cell Counting Kit-8 (CCK-8) (Bimake, USA) according to the manufacturer's instructions. Briefly, cells were treated with CCK-8 solution and the absorbance was measured at 450 nm using a microplate reader.

For colony formation assays, HCC cells were seeded into 6-well plates at a density of 10^4 cells/well and incubated at 37 °C for 2 weeks. Colonies were fixed with 4% phosphate-buffered formalin (pH 7.4) and subjected to Giemsa staining for 15 minutes. Each assay was performed in three separate experiments.

Transmission electron microscopy and mitochondrial quantification

Cells were harvested and processed for transmission electron microscopy (TEM) using standard procedures. Ultrathin sections were examined by TEM, and representative images were acquired at the same magnification under identical imaging conditions. For quantitative analysis, individual mitochondria were manually outlined using ImageJ software. Mitochondrial area was measured and expressed as μm^2. Mitochondrial electron density was evaluated by measuring the mean gray value within each mitochondrial region of interest (ROI), with lower gray values indicating higher electron density. Multiple mitochondria from randomly selected fields were analyzed for each group. All images were analyzed using identical threshold and measurement settings.

Targeted metabolomic analysis

For targeted metabolomic analysis, 3×10^6 cells were seeded in 10-cm dishes and cultured to ~90% confluence. Cells were washed twice with ice-cold PBS and quenched with 1 mL pre-chilled methanol containing 2-chloro-L-phenylalanine (2 μg/mL) as an internal standard. Metabolites were extracted by sonication, followed by centrifugation at 13,000 × g for 15 min at 4 °C. The supernatants were transferred to fresh glass vials for analysis. Targeted metabolomic profiling was performed using an Agilent 1290 UHPLC system coupled to an AB QTRAP 6500 mass

spectrometer (AB Sciex). Metabolite separation was achieved on a Waters ACQUITY UPLC BEH Amide column (1.7 µm, 2.1 × 100 mm). The mass spectrometer was operated in multiple reaction monitoring (MRM) mode. MRM transitions, declustering potentials (DPs), and collision energies (CEs) for cancer metabolism–related metabolites were adopted from previous studies. Data acquisition was performed using Analyst software (version 1.6.1, AB Sciex), and chromatographic peak integration was conducted using OS software (version 1.5.0.23389, AB Sciex). Metabolite abundances were quantified as peak area ratios relative to the internal standard. Metabolites with $P < 0.05$ (two-tailed Student's t-test) were considered significantly altered.

RNA-sequencing (RNA-seq)

Total RNA was extracted from shNC- or shPSAT1-transduced Huh7 cells treated with DMSO, erastin, or sorafenib (5×10^6 cells per sample, n = 3 biological replicates per group) using TRIzol reagent. RNA integrity and quality were assessed prior to library preparation. Poly(A)+ mRNA was enriched using oligo(dT) magnetic beads, followed by library construction according to standard protocols. Paired-end sequencing was performed on an Illumina sequencing platform. Raw sequencing reads were subjected to quality control using FastQC and aligned to the human reference genome (GRCh38) using HISAT2. Gene expression levels were quantified as fragments per kilobase of transcript per million mapped reads (FPKM). Differentially expressed genes were identified using a cutoff of $|\log2 \text{ fold change}| > 1$ and adjusted P value < 0.05.

Single-Cell RNA data analysis

Single-cell RNA-seq data were obtained from the GEO database (GSE292298) to analyze PSAT1 mRNA expression in human hepatocellular carcinoma (HCC) tissues. Raw DIPSEQ-T1

sequencing reads were processed using PISA (v0.2) and aligned to the human genome with STAR (v2.7.4a). Gene–cell count matrices were generated and subjected to stringent quality control to remove low-quality cells, doublets, and potential contaminants. Cells with fewer than 200 or more than 6,000 detected genes, or with >20% mitochondrial UMI content, were excluded. Doublets were further identified and filtered using DoubletFinder in combination with clustering patterns and marker gene expression. After quality control, a total of 59,998 single-cell transcriptomes from tumor tissues of six HCC patients were retained for downstream analyses. UMI counts were normalized to total counts per cell, scaled to a common library size, and log-transformed prior to subsequent analyses.

According to the definition of cell clusters based on the specific genetic markers of different cells, GSE292298 results showed that HCC had a total of eleven cell clusters (myeloid/monocytic cells, $CD4^+$ T cells, $CD8^+$ T cells, B/plasma cells, endothelial/fibroblast cells, cholangiocytes, pericytes, neutrophils, mast cells, malignant hepatocytes (major) and malignant hepatocytes (minor)). The cell clusters were re-analyzed with CopyKAT to distinguish malignant cells from other cells according to chromosome copy number variation (CNV). To infer cell-cell communication patterns among different cell types in the scRNA-seq data, we employed the CellChat package (https://github.com/jinworks/CellChat). Malignant hepatocytes were stratified into PSAT1-high and PSAT1-low groups based on normalized expression levels. Differential gene expression analysis was performed using Seurat, and pathway activity was evaluated by module scoring of curated gene signatures, including the unfolded protein response (UPR) gene set. All visualizations were generated in R using Seurat and ggplot2.

Flow cytometry analysis

Tumor tissues were harvested and digested in RPMI medium containing 0.1% collagenase type IV and 0.01% DNase I at 37 °C for 30 min with gentle agitation. Digested tissues were passed through a 70-μm cell strainer to obtain single-cell suspensions and washed twice with PBS. Cells were stained with DAPI (C1002, Beyotime, China) or LIVE/DEAD™ Fixable Violet Dead Cell Stain Kit (L34963, Thermo Fisher Scientific) to exclude dead cells. Prior to surface staining, Fc receptors were blocked using anti-CD16/CD32 monoclonal antibody (1:50; MFCR00-4, Thermo Fisher Scientific) for 15 min at 4 °C. Cells were then incubated with fluorophore-conjugated antibodies against surface markers at appropriate dilutions for 30 min on ice in the dark. For intracellular staining, cells were fixed using Fixation Buffer (420801, BioLegend), permeabilized with Intracellular Staining Permeabilization Wash Buffer (421002, BioLegend), and incubated with intracellular antibodies for 30 min at 4 °C. Immune cell populations were analyzed within the live $CD45^+$ cell gate. Flow cytometric acquisition was performed on a BD LSRFortessa™ X-20 flow cytometer (BD Biosciences). Compensation was set using single-stained controls. Data were analyzed using FlowJo software (Tree Star). A complete list of fluorophore-conjugated antibodies is provided in Supplementary Table S3.

Malondialdehyde (MDA), glutathione (GSH) and iron assay

The levels of MDA (Beyotime, China), GSH and glutathione disulfide (GSSG) (Beyotime, China) and ferrous ion content (Abcam; ab83366) were quantified using respective assay kits according to the instructions. Sample absorbance was quantified using a microplate reader. The values were presented as the mean absorbance normalized to the percentage of the normal control.

Quantification of α-KG abundance

Intracellular α-ketoglutarate (α-KG) levels were quantified using a commercial assay kit (S0323S, Beyotime, China) according to the manufacturer's instructions. Briefly, cell lysates prepared from 1×10^6 cells per sample were subjected to α-KG measurement, and absorbance values were normalized to cell number.

Lipid peroxidation assay

Lipid peroxidation was determined by C11-BODIPY staining according to the manufacturer's protocol (D3861, Thermo Fisher Scientific). Briefly, cells were treated with drugs for an appropriate time and then treated with 2 μM C11-BODIPY for 30 min. After being harvested, washed, and resuspended in PBS, the levels of lipid peroxidation were determined by flow cytometry. The data analysis was performed by using FlowJo Version 7.6 software.

Multiplex immunofluorescence staining

Multiplex immunofluorescence staining was performed using a tyramide signal amplification (TSA) kit (RK05905P, ABclonal, China) according to the manufacturer's instructions. Briefly, tissue sections were deparaffinized, rehydrated, and subjected to antigen retrieval. After blocking, sections were incubated with the indicated primary antibodies, followed by the corresponding HRP-conjugated secondary antibodies and TSA fluorophore working solution. After each staining cycle, antibody complexes were removed by heat-mediated antigen retrieval before incubation with the next primary antibody. This procedure was repeated sequentially for each target. Nuclei were counterstained with DAPI. Fluorescence images were acquired using a Leica TCS SP8 confocal microscope (Leica Microsystems). Information on the antibodies used is provided in Supplementary Table S3.

Luciferase assay

The CCL5 promoter (bp -982/+3 relative to the ATG start codon) was cloned and inserted into the luciferase reporter gene vector pGL3-enhance (Promega, Madison WI). Three 5'-truncated sequences of the CCL5 promoter were generated by PCR and cloned and inserted into the luciferase reporter gene vector pGL3-enhance. The primer sequences are listed in Supplementary Table S2. Cells were cotransfected with the corresponding reporter plasmid and the indicated plasmids in each experiment according to the standard protocol as described previously. Luciferase activity was detected using a dual-luciferase report assay (Promega) system in accordance with the manufacturer's instructions. Renilla luciferase activity was normalized against firefly luciferase activity.

mRNA decay analysis

For mRNA stability experiments, cells were treated with 10 μM actinomycin D (Actino D) to block the de novo mRNA synthesis for various times. Total RNA was isolated and HSPA5 mRNA expression was determined by qPCR. The relative amount of HSPA5 mRNA without Actino D treatment was set to 100%, and the percentage of HSPA5 mRNA was calculated accordingly. The relative change of HSPA5 mRNA amounts was normalized to GAPDH mRNA.

Bone marrow-derived macrophages (BMDMs)

The femurs and tibias of 6–8-week-old mice were dissected to extract bone marrow cells. These cells were cultured in DMEM supplemented with 10% FBS, 100 U/mL penicillin, 100 U/mL

streptomycin, and 40 ng/mL macrophage colony-stimulating factor (M-CSF) (abs04383, Absin, Shanghai, China) for 7 days to induce macrophage differentiation.

Transwell assay

To assess cell migratory capability, Transwell assays were conducted. Initially, 100 ng/mL phorbol 12-myristate 13-acetate (PMA, P1585, Sigma)-treated THP1 cells (5×10^4) or BMDMs (2×10^5) were seeded in the upper chamber (3422, Corning), while Huh7 or Hep3B or HCC-LY10 cells or Hepa1-6 or Hep53.4 cells were seeded in the lower chamber. After 48 h of incubation, non-migrating cells were removed using cotton swabs. Subsequently, the cells at the bottom of the chamber were fixed with paraformaldehyde, stained with crystal violet, and observed under an inverted microscope (Olympus, Tokyo, Japan).

For Treg migration assays, purified mouse Treg cells (4×10^4 cells) were seeded into the upper chamber, while Hep53.4 cells with PSAT1 knockdown or overexpression, together with their corresponding control cells, were cultured in the lower chamber. Where indicated, maraviroc (MVC) or recombinant mouse CCL5 was added to evaluate the involvement of the CCL5–CCR5 axis. After 4 h of incubation, migrated Treg cells in the lower chamber were collected and quantified by flow cytometry using counting beads. The number of migrated Treg cells was calculated based on the ratio of Treg events to counting-bead events and expressed relative to the corresponding control group.

Coculture assay

To conduct the coculture experiment, we utilized 6-well Transwell chambers with 0.4 μm pores (PTHT06H48, Corning). THP1 cells (1×10^6) were treated with PMA at a concentration of 100

ng/mL for 24 h. Similarly, BMDMs were introduced into the lower Transwell chambers. In the upper chamber of the coculture system, we seeded 5×10^5 Huh7 or MHCC-97H or Hep3B or HCC-LY10 cells or Hepa1-6 or Hep53.4 cells for 48 h. Subsequently, we separately collected macrophages for qRT-PCR and flow cytometry analyses.

ELISA

The ELISA was conducted following the manufacturer's guidelines. Cell culture supernatants were collected at 48 h and clarified by filtration through a 0.45-μm filter. The concentrations of secreted CCL5 proteins in the culture media supernatant were quantified using the CCL5 ELISA Kit (abs5510726, absin, China). For murine blood, allow clot formation at room temperature (approximately 25°C), followed by centrifugation at 4°C and 10,000 × g for 10 min to collect the supernatant. CCL5 was measured using a mouse CCL5 assay kit (KTE3010, abbkine, China)

Isolation, culture, and functional assays of primary CD8$^+$ T cells and Treg cells

Spleens were harvested from C57BL/6 mice and mechanically dissociated through a 70-μm cell strainer to obtain single-cell suspensions. Red blood cells were removed using red blood cell lysis buffer, and the remaining splenocytes were washed and resuspended in staining buffer. CD8$^+$ T cells were isolated by flow cytometric sorting as CD45$^+$CD3$^+$CD8$^+$ cells, whereas Treg cells were sorted as CD45$^+$CD4$^+$CD25$^+$CD127$^-$ cells. Sorted cells were collected into complete RPMI-1640 medium supplemented with 10% fetal bovine serum, 1% penicillin–streptomycin, and 2 mM L-glutamine and maintained at 37 °C in a humidified incubator with 5% CO_2.

For $CD8^+$ T-cell activation, purified $CD8^+$ T cells were stimulated with anti-mouse CD3/CD28 activation beads at a bead-to-cell ratio of 1:1 in complete medium supplemented with recombinant mouse IL-2.

To evaluate the effect of CCL5 on Treg suppressive activity, purified Treg cells were pretreated with recombinant mouse CCL5 at 10 or 50 ng/mL for 24 h and subsequently co-cultured with activated $CD8^+$ T cells at a CD8:Treg ratio of 4:1. $CD8^+$ T cells cultured alone served as controls. After co-culture, $CD8^+$ T-cell apoptosis was assessed by flow cytometry using the indicated apoptosis staining reagents.

For intracellular cytokine analysis, cells were stimulated with eBioscience™ Cell Stimulation Cocktail (plus protein transport inhibitors) (500×; Cat. No. 00-4975-93, Thermo Fisher Scientific) at 1× for 6 h. Cells were then stained for surface CD8, followed by fixation and permeabilization and intracellular staining for granzyme B (GZMB) and IFN-γ. The proportions of apoptotic, $GZMB^+$, and IFN-γ^+ cells within the $CD8^+$ T-cell population were quantified by flow cytometry.

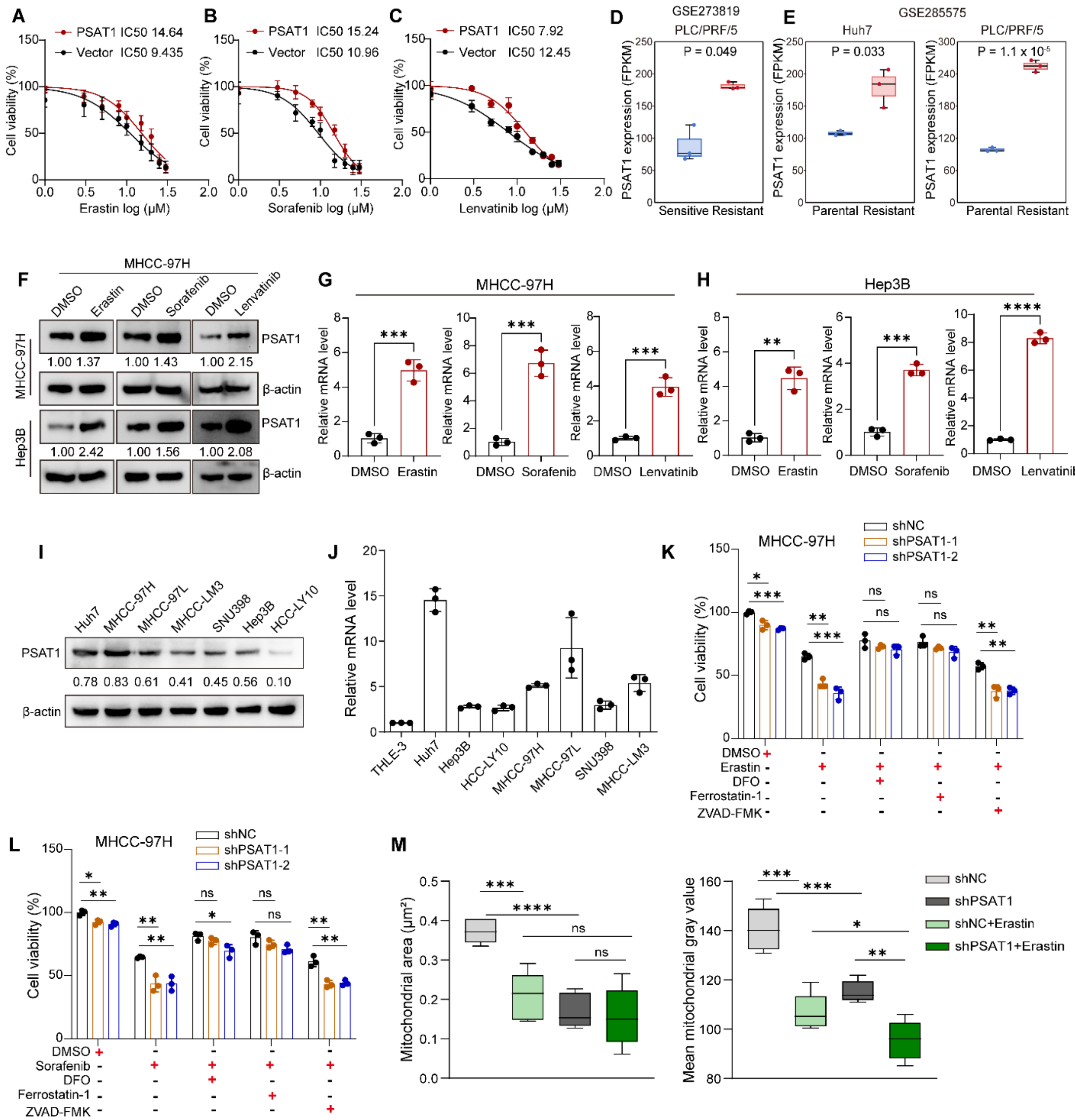
A
PSAT1 IC50 14.64
Vector IC50 9.435
Cell viability (%)
Erastin log (μM)
B
PSAT1 IC50 15.24
Vector IC50 10.96
Cell viability (%)
Sorafenib log (μM)
C
PSAT1 IC50 7.92
Vector IC50 12.45
Cell viability (%)
Lenvatinib log (μM)
D
GSE273819
PLC/PRF/5
P = 0.049
PSAT1 expression (FPKM)
Sensitive Resistant
E
GSE285575
Huh7
P = 0.033
PSAT1 expression (FPKM)
Parental Resistant
PLC/PRF/5
P = 1.1 x 10-5
PSAT1 expression (FPKM)
Parental Resistant
F
MHCC-97H
DMSO Erastin DMSO Sorafenib DMSO Lenvatinib
MHCC-97H
PSAT1
1.00 1.37 1.00 1.43 1.00 2.15
β-actin
Hep3B
PSAT1
1.00 2.42 1.00 1.56 1.00 2.08
β-actin
G
MHCC-97H
Relative mRNA level
DMSO Erastin
DMSO Sorafenib
DMSO Lenvatinib
H
Hep3B
Relative mRNA level
DMSO Erastin
DMSO Sorafenib
DMSO Lenvatinib
I
Huh7 MHCC-97H MHCC-97L MHCC-LM3 SNU398 Hep3B HCC-LY10
PSAT1
0.78 0.83 0.61 0.41 0.45 0.56 0.10
β-actin
J
Relative mRNA level
THLE-3 Huh7 Hep3B HCC-LY10 MHCC-97H MHCC-97L SNU398 MHCC-LM3
K
MHCC-97H
shNC
shPSAT1-1
shPSAT1-2
Cell viability (%)
DMSO
Erastin
DFO
Ferrostatin-1
ZVAD-FMK
L
MHCC-97H
shNC
shPSAT1-1
shPSAT1-2
Cell viability (%)
DMSO
Sorafenib
DFO
Ferrostatin-1
ZVAD-FMK
M
Mitochondrial area (μm²)
Mean mitochondrial gray value
shNC
shPSAT1
shNC+Erastin
shPSAT1+Erastin

Figure S1. Targeting PSAT1 sensitizes tumor cells to ferroptosis. (A–C) Dose–response curves of PSAT1-overexpressing and control HCC cells treated with erastin, sorafenib, or lenvatinib. (D–E) PSAT1 expression in lenvatinib-sensitive/resistant or parental/resistant HCC cells from GSE273819 and GSE285575. (F–H) PSAT1 protein and mRNA expression following erastin, sorafenib, or lenvatinib treatment. (I–J) PSAT1 protein and mRNA expression across HCC cell lines. (K–L) Cell viability of shNC and PSAT1-knockdown MHCC-97H cells treated with erastin (K) or sorafenib (L), with or without DFO, ferrostatin-1, or ZVAD-FMK. (M) Quantification of mitochondrial area and mean gray value by TEM. $*p < 0.05$; $**p < 0.01$.

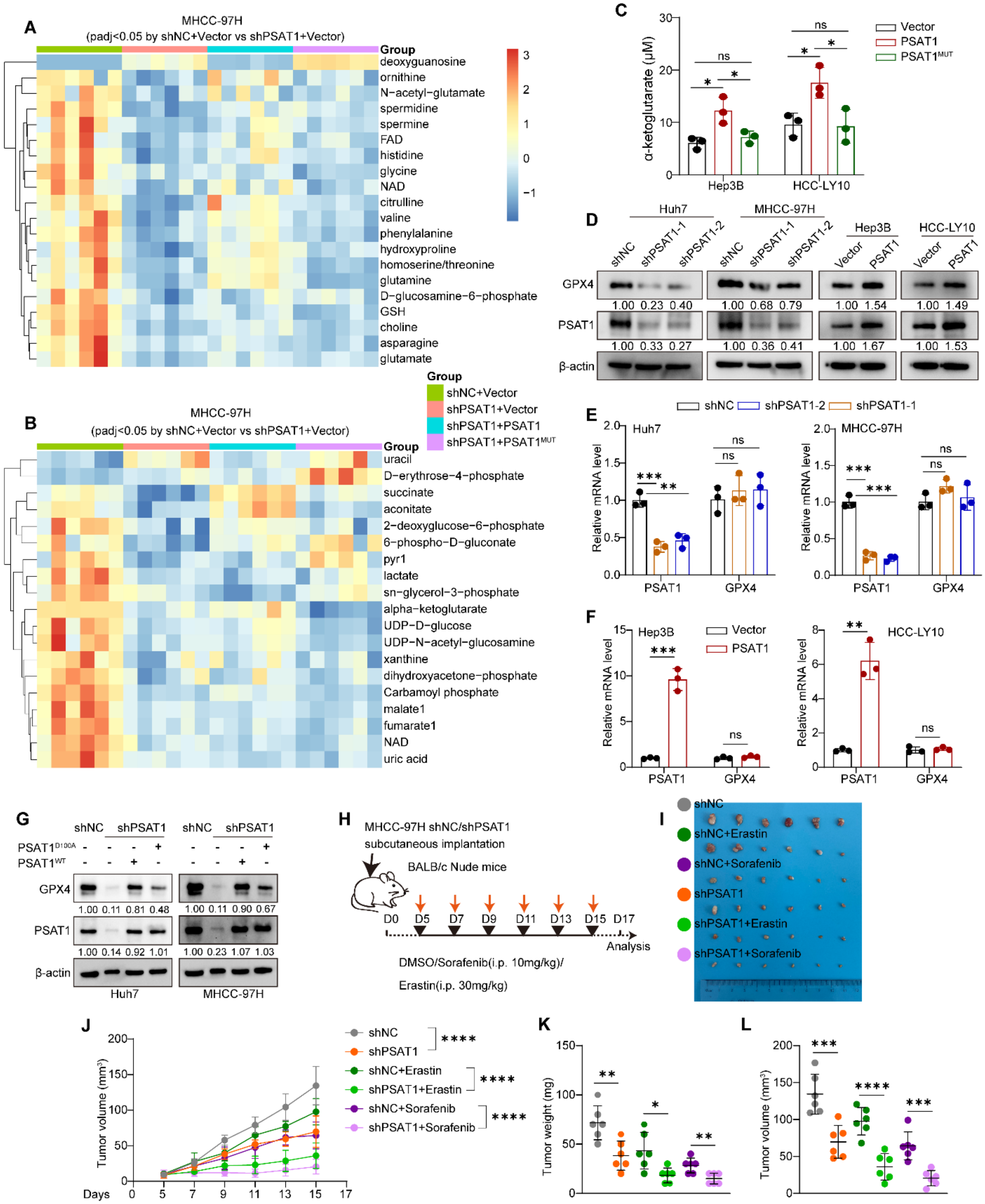
A
MHCC-97H
(padj<0.05 by shNC+Vector vs shPSAT1+Vector)
Group
deoxyguanosine
ornithine
N−acetyl−glutamate
spermidine
spermine
FAD
histidine
glycine
NAD
citrulline
valine
phenylalanine
hydroxyproline
homoserine/threonine
glutamine
D−glucosamine−6−phosphate
GSH
choline
asparagine
glutamate
3
2
1
0
−1
Group
shNC+Vector
shPSAT1+Vector
shPSAT1+PSAT1
shPSAT1+PSAT1MUT
B
MHCC-97H
(padj<0.05 by shNC+Vector vs shPSAT1+Vector)
Group
uracil
D−erythrose−4−phosphate
succinate
aconitate
2−deoxyglucose−6−phosphate
6−phospho−D−gluconate
pyr1
lactate
sn−glycerol−3−phosphate
alpha−ketoglutarate
UDP−D−glucose
UDP−N−acetyl−glucosamine
xanthine
dihydroxyacetone−phosphate
Carbamoyl phosphate
malate1
fumarate1
NAD
uric acid
C
α-ketoglutarate (μM)
Vector
PSAT1
PSAT1MUT
ns
Hep3B
HCC-LY10
D
Huh7
MHCC-97H
Hep3B
HCC-LY10
shNC shPSAT1-1 shPSAT1-2 shNC shPSAT1-1 shPSAT1-2 Vector PSAT1 Vector PSAT1
GPX4
1.00 0.23 0.40 1.00 0.68 0.79 1.00 1.54 1.00 1.49
PSAT1
1.00 0.33 0.27 1.00 0.36 0.41 1.00 1.67 1.00 1.53
β-actin
E
shNC shPSAT1-2 shPSAT1-1
Huh7
MHCC-97H
Relative mRNA level
PSAT1
GPX4
F
Hep3B
HCC-LY10
Vector
PSAT1
Relative mRNA level
PSAT1
GPX4
G
shNC shPSAT1 shNC shPSAT1
PSAT1D100A
PSAT1WT
GPX4
1.00 0.11 0.81 0.48 1.00 0.11 0.90 0.67
PSAT1
1.00 0.14 0.92 1.01 1.00 0.23 1.07 1.03
β-actin
Huh7
MHCC-97H
H
MHCC-97H shNC/shPSAT1
subcutaneous implantation
BALB/c Nude mice
D0 D5 D7 D9 D11 D13 D15 D17
Analysis
DMSO/Sorafenib(i.p. 10mg/kg)/
Erastin(i.p. 30mg/kg)
I
shNC
shNC+Erastin
shNC+Sorafenib
shPSAT1
shPSAT1+Erastin
shPSAT1+Sorafenib
J
Tumor volume (mm3)
Days
shNC
shPSAT1
shNC+Erastin
shPSAT1+Erastin
shNC+Sorafenib
shPSAT1+Sorafenib
****
K
Tumor weight (mg)
L
Tumor volume (mm3)

Figure S2. PSAT1 supports ferroptosis resistance in HCC cells and tumors. (A-B) Heatmaps of significantly altered metabolites following PSAT1 knockdown and rescue with wild-type or mutant PSAT1. (C) Intracellular α-ketoglutarate levels in Hep3B and HCC-LY10 cells expressing vector control, wild-type PSAT1, or the enzymatically inactive PSAT1 mutant ($PSAT1^{MUT}$). (D-F) GPX4 protein and mRNA expression following PSAT1 knockdown or overexpression. (G) GPX4 protein expression following reconstitution with wild-type PSAT1 or PSAT1-D100A. (H-L) Subcutaneous MHCC-97H xenograft model, representative tumors, tumor growth curves, tumor weights, and endpoint tumor volumes following the indicated treatments.*$p < 0.05$; **$p < 0.01$.

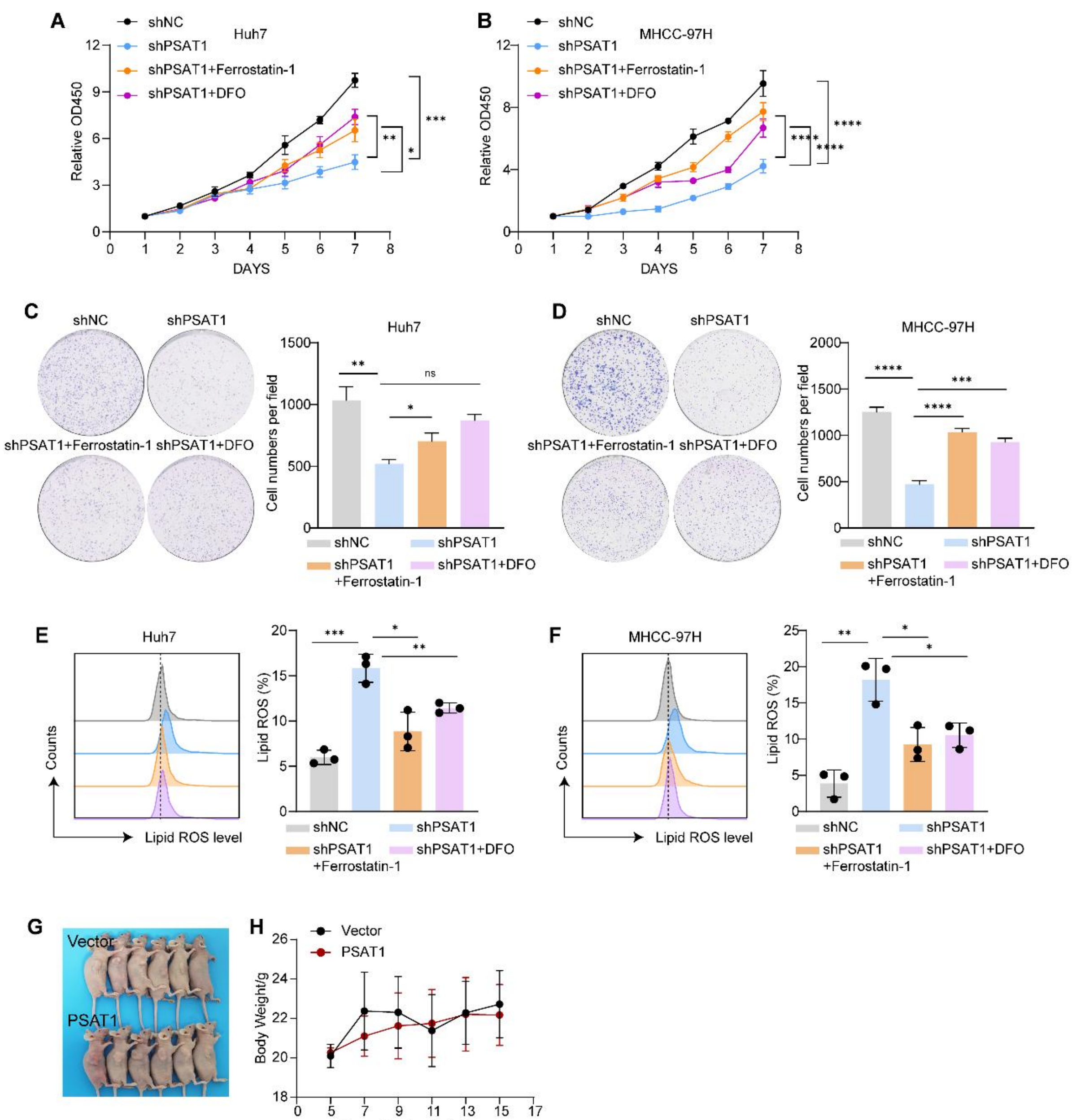
A
Huh7
shNC
shPSAT1
shPSAT1+Ferrostatin-1
shPSAT1+DFO
Relative OD450
DAYS
B
MHCC-97H
shNC
shPSAT1
shPSAT1+Ferrostatin-1
shPSAT1+DFO
Relative OD450
DAYS
C
shNC
shPSAT1
shPSAT1+Ferrostatin-1
shPSAT1+DFO
Huh7
Cell numbers per field
ns
shPSAT1
+Ferrostatin-1
D
MHCC-97H
Cell numbers per field
E
Huh7
Counts
Lipid ROS level
Lipid ROS (%)
F
MHCC-97H
G
Vector
PSAT1
H
Body Weight/g
Days after implantation

Figure S3. Additional evidence that PSAT1 promotes HCC growth and protects against ferroptotic stress. (A–B) Cell proliferation of PSAT1-knockdown Huh7 and MHCC-97H cells treated with ferrostatin-1 or DFO. (C–D) Representative colony formation assays and quantification showing rescue of the reduced clonogenic capacity caused by PSAT1 depletion. (E–F) Flow cytometric analysis and quantification of lipid ROS levels in PSAT1-knockdown cells with or without ferrostatin-1 or DFO. (G) Representative images of mice bearing vector- or PSAT1-expressing tumors. (H) Body weight curves of mice during tumor growth. *p < 0.05; *p < 0.01; *p < 0.001; *p < 0.0001.

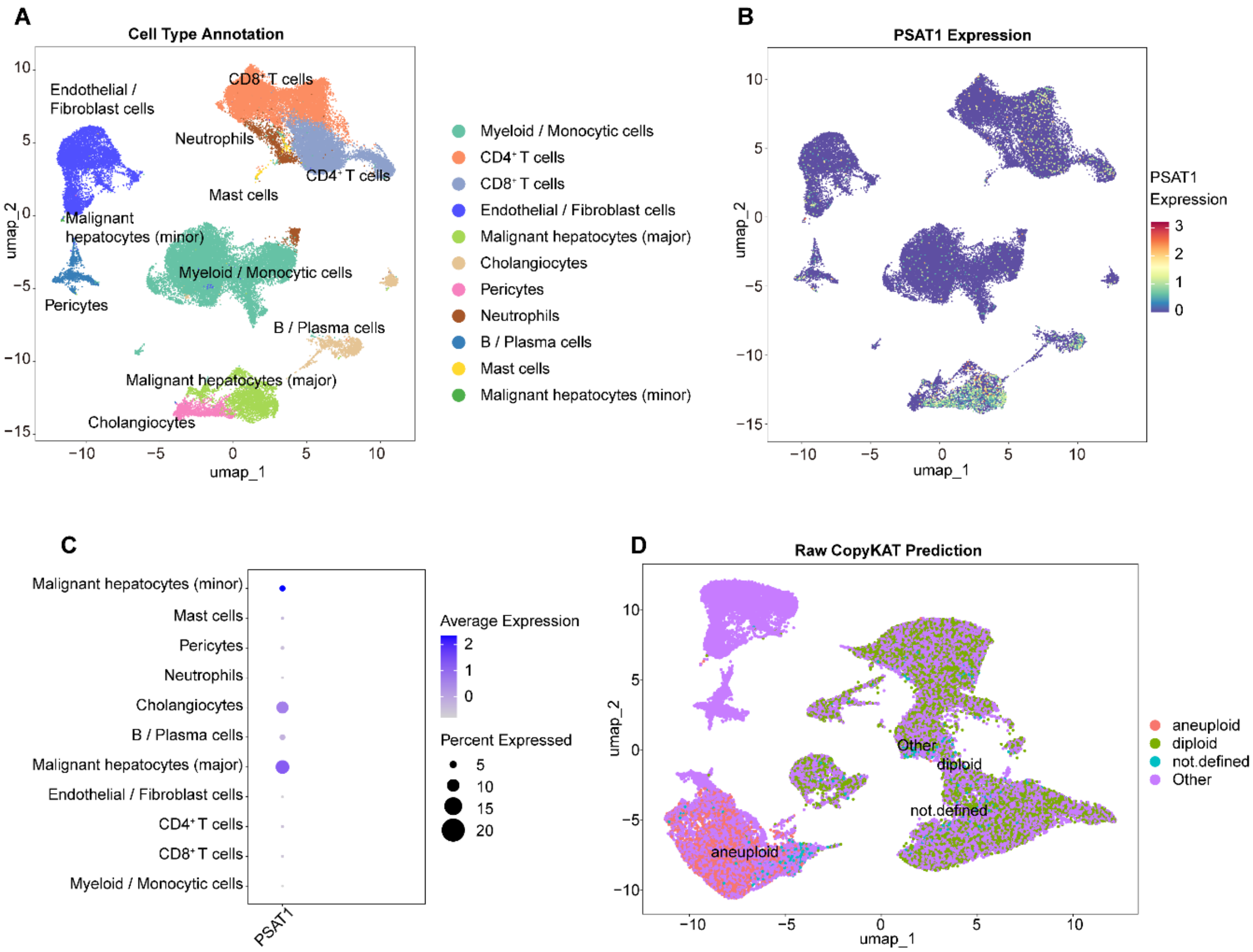
A
Cell Type Annotation
Endothelial / Fibroblast cells
CD8⁺ T cells
Neutrophils
CD4⁺ T cells
Mast cells
Malignant hepatocytes (minor)
Myeloid / Monocytic cells
Pericytes
B / Plasma cells
Malignant hepatocytes (major)
Cholangiocytes
umap_1
umap_2
Myeloid / Monocytic cells
CD4⁺ T cells
CD8⁺ T cells
Endothelial / Fibroblast cells
Malignant hepatocytes (major)
Cholangiocytes
Pericytes
Neutrophils
B / Plasma cells
Mast cells
Malignant hepatocytes (minor)
B
PSAT1 Expression
PSAT1 Expression
umap_1
umap_2
C
Malignant hepatocytes (minor)
Mast cells
Pericytes
Neutrophils
Cholangiocytes
B / Plasma cells
Malignant hepatocytes (major)
Endothelial / Fibroblast cells
CD4⁺ T cells
CD8⁺ T cells
Myeloid / Monocytic cells
PSAT1
Average Expression
Percent Expressed
D
Raw CopyKAT Prediction
Other
diploid
not.defined
aneuploid
umap_1
umap_2
aneuploid
diploid
not.defined
Other

Figure S4. Single-cell transcriptomic characterization of PSAT1 expression and malignant hepatocytes in HCC. (A) UMAP visualization of major cell populations identified by single-cell RNA sequencing (scRNA-seq) of human HCC samples. Cell populations were annotated according to canonical lineage markers. (B) UMAP feature plot showing the distribution of PSAT1 expression across the identified cell populations. (C) Dot plot showing PSAT1 expression across major cell populations. Dot size represents the percentage of PSAT1-expressing cells, and color intensity indicates the average expression level. (D) CopyKAT-based UMAP showing inferred aneuploid, diploid, and undefined cell populations.

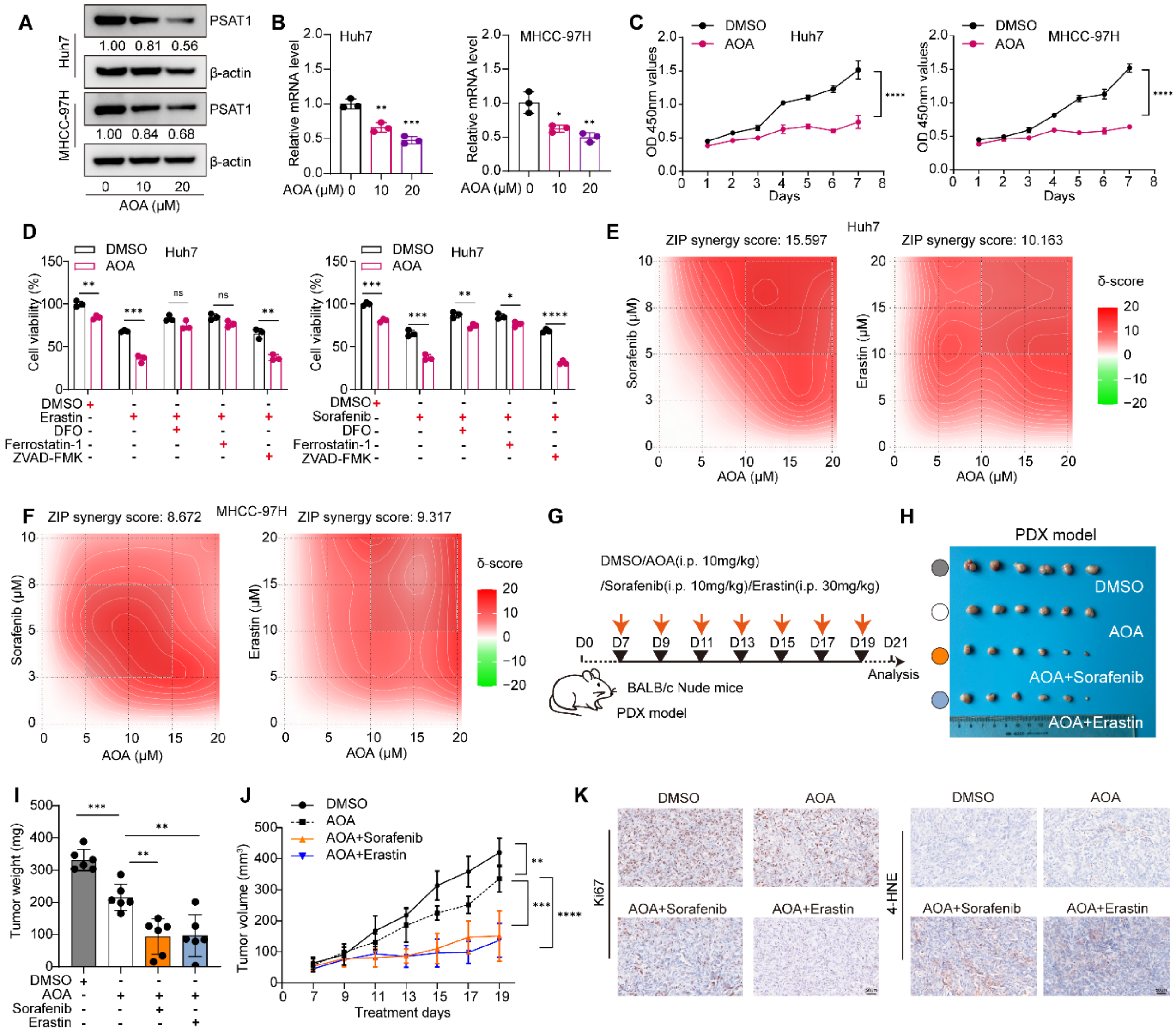

A
PSAT1
1.00 0.81 0.56
β-actin
Huh7
PSAT1
1.00 0.84 0.68
β-actin
MHCC-97H
0 10 20
AOA (μM)
B
Huh7
MHCC-97H
Relative mRNA level
AOA (μM) 0 10 20
C
DMSO
AOA
Huh7
MHCC-97H
OD 450nm values
Days
D
DMSO
AOA
Huh7
Cell viability (%)
DMSO
Erastin
DFO
Ferrostatin-1
ZVAD-FMK
Sorafenib
E
Huh7
ZIP synergy score: 15.597
ZIP synergy score: 10.163
Sorafenib (μM)
Erastin (μM)
AOA (μM)
δ-score
F
ZIP synergy score: 8.672
MHCC-97H
ZIP synergy score: 9.317
δ-score
G
DMSO/AOA(i.p. 10mg/kg)
/Sorafenib(i.p. 10mg/kg)/Erastin(i.p. 30mg/kg)
D0 D7 D9 D11 D13 D15 D17 D19 D21
Analysis
BALB/c Nude mice
PDX model
H
PDX model
DMSO
AOA
AOA+Sorafenib
AOA+Erastin
I
Tumor weight (mg)
DMSO
AOA
Sorafenib
Erastin
J
DMSO
AOA
AOA+Sorafenib
AOA+Erastin
Tumor volume (mm³)
Treatment days
K
DMSO
AOA
AOA+Sorafenib
AOA+Erastin
Ki67
4-HNE

Figure S5. AOA sensitizes HCC cells to ferroptosis-based therapy. (A–B) Effects of aminooxyacetic acid (AOA) treatment for 48 h on PSAT1 protein (A) and mRNA (B) expression in Huh7 and MHCC-97H cells, as assessed by immunoblotting and qRT–PCR, respectively. (C) Cell proliferation following AOA treatment, assessed by CCK-8 assay. (D) Effects of AOA on erastin- or sorafenib-induced loss of cell viability in Huh7 cells in the presence or absence of ferrostatin-1, deferoxamine (DFO), or Z-VAD-FMK, assessed after 48 h. (E-F) Drug interaction analysis performed using SynergyFinder, showing the combinatorial effects of AOA with sorafenib (E) or erastin (F). Red indicates synergistic interactions, white additive effects, and green antagonistic interactions across the tested dose matrices. (G) Schematic illustration of the in vivo experimental design. (H) Representative images of excised tumors at the experimental endpoint. (I) Quantification of tumor weights at sacrifice. (J) Tumor growth curves showing tumor volume over time in patient-derived xenograft (PDX) models under the indicated treatment conditions. (K) Representative immunohistochemistry (IHC) staining of Ki67 and 4-hydroxynonenal (4-HNE) in subcutaneous tumor tissues from the indicated treatment groups. Data are presented as mean ± SEM. $*p < 0.05$; $**p < 0.01$.

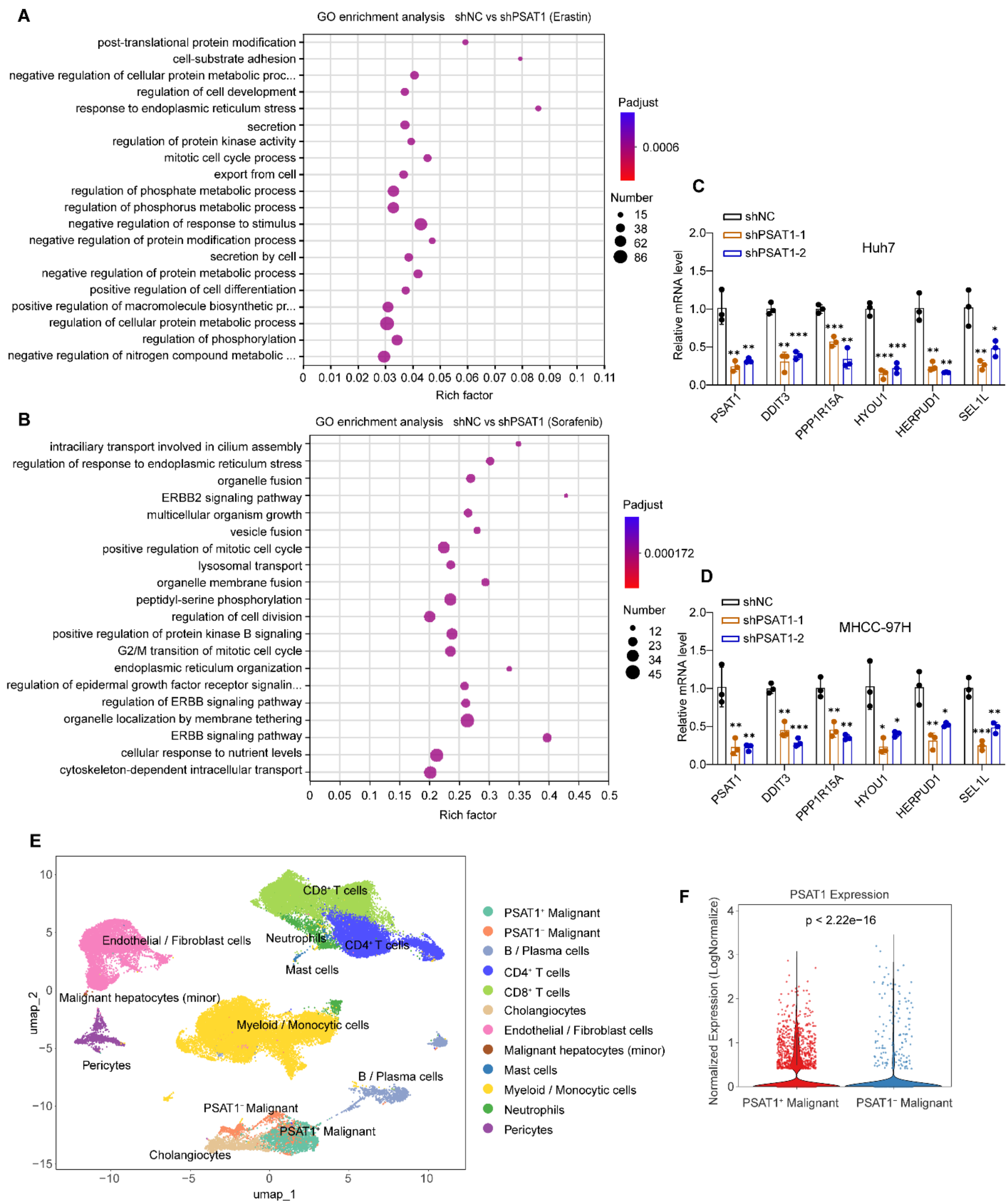
A
GO enrichment analysis shNC vs shPSAT1 (Erastin)
post-translational protein modification
cell-substrate adhesion
negative regulation of cellular protein metabolic proc...
regulation of cell development
response to endoplasmic reticulum stress
secretion
regulation of protein kinase activity
mitotic cell cycle process
export from cell
regulation of phosphate metabolic process
regulation of phosphorus metabolic process
negative regulation of response to stimulus
negative regulation of protein modification process
secretion by cell
negative regulation of protein metabolic process
positive regulation of cell differentiation
positive regulation of macromolecule biosynthetic pr...
regulation of cellular protein metabolic process
regulation of phosphorylation
negative regulation of nitrogen compound metabolic ...
Rich factor
Padjust
0.0006
Number
15
38
62
86
B
GO enrichment analysis shNC vs shPSAT1 (Sorafenib)
intraciliary transport involved in cilium assembly
regulation of response to endoplasmic reticulum stress
organelle fusion
ERBB2 signaling pathway
multicellular organism growth
vesicle fusion
positive regulation of mitotic cell cycle
lysosomal transport
organelle membrane fusion
peptidyl-serine phosphorylation
regulation of cell division
positive regulation of protein kinase B signaling
G2/M transition of mitotic cell cycle
endoplasmic reticulum organization
regulation of epidermal growth factor receptor signalin...
regulation of ERBB signaling pathway
organelle localization by membrane tethering
ERBB signaling pathway
cellular response to nutrient levels
cytoskeleton-dependent intracellular transport
Rich factor
Padjust
0.000172
Number
12
23
34
45
C
shNC
shPSAT1-1
shPSAT1-2
Huh7
Relative mRNA level
PSAT1
DDIT3
PPP1R15A
HYOU1
HERPUD1
SEL1L
D
shNC
shPSAT1-1
shPSAT1-2
MHCC-97H
Relative mRNA level
PSAT1
DDIT3
PPP1R15A
HYOU1
HERPUD1
SEL1L
E
CD8⁺ T cells
Endothelial / Fibroblast cells
Neutrophils
CD4⁺ T cells
Mast cells
Malignant hepatocytes (minor)
Myeloid / Monocytic cells
Pericytes
B / Plasma cells
PSAT1⁻ Malignant
PSAT1⁺ Malignant
Cholangiocytes
umap_1
umap_2
PSAT1⁺ Malignant
PSAT1⁻ Malignant
B / Plasma cells
CD4⁺ T cells
CD8⁺ T cells
Cholangiocytes
Endothelial / Fibroblast cells
Malignant hepatocytes (minor)
Mast cells
Myeloid / Monocytic cells
Neutrophils
Pericytes
F
PSAT1 Expression
p < 2.22e−16
Normalized Expression (LogNormalize)
PSAT1⁺ Malignant
PSAT1⁻ Malignant

Figure S6. PSAT1 confers ferroptosis resistance via the ER stress response. (A–B) Gene Ontology (GO) enrichment analysis of differentially expressed genes between shNC and shPSAT1 cells following erastin (A) or sorafenib (B) treatment. Enriched biological process terms are ranked by rich factor; dot size represents gene count, and color indicates adjusted P value. (C–D) qRT–PCR analysis of PSAT1 and ER stress–related genes (DDIT3, PPP1R15A, HYOU1, HERPUD1, and SEL1L) in Huh7 (C) and MHCC-97H (D) cells transduced with control shRNA (shNC) or two independent PSAT1-targeting shRNAs (shPSAT1-1/2). (E) UMAP visualization of single-cell RNA-seq data showing major cell populations in HCC, with malignant hepatocytes further stratified into $PSAT1^{+}$ and $PSAT1^{-}$ subsets based on PSAT1 expression. (F) Violin plot comparing normalized PSAT1 expression levels between $PSAT1^{+}$ and $PSAT1^{-}$ malignant hepatocytes. Statistical significance was assessed using the Wilcoxon rank-sum test. * $p < 0.05$; ** $p < 0.01$.

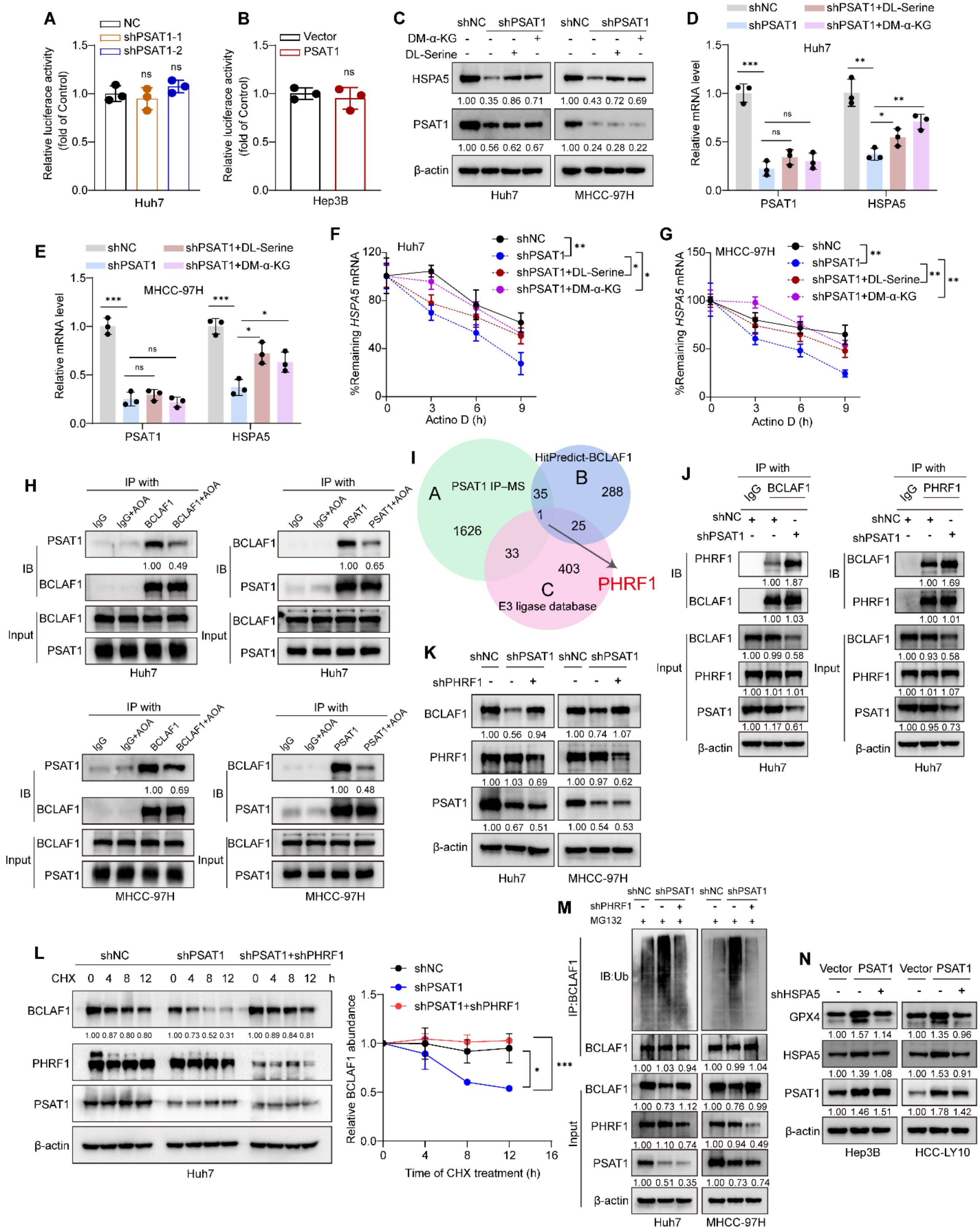

A
B
C
D
E
F
G
H
I
J
K
L
M
N
Huh7
Hep3B
MHCC-97H
HCC-LY10
shNC
shPSAT1
shPSAT1+DL-Serine
shPSAT1+DM-α-KG
shPSAT1+shPHRF1
Relative luciferase activity (fold of Control)
Relative mRNA level
%Remaining HSPA5 mRNA
Actino D (h)
Relative BCLAF1 abundance
Time of CHX treatment (h)
PSAT1 IP–MS
HitPredict-BCLAF1
E3 ligase database
PHRF1
IP with
IB
Input
IP:BCLAF1
IB:Ub

Figure S7. PSAT1 regulates HSPA5 expression and BCLAF1 stability through metabolic and PHRF1-dependent mechanisms. (A–B) HSPA5 promoter luciferase activity in Huh7 cells with PSAT1 knockdown and Hep3B cells with PSAT1 overexpression. (C–E) Immunoblot and qRT–PCR analyses of HSPA5 and PSAT1 expression in PSAT1-knockdown Huh7 and MHCC-97H cells supplemented with DL-serine or DM-α-KG. (F–G) Actinomycin D chase assays showing HSPA5 mRNA stability under the same treatment conditions. (H) PSAT1–BCLAF1 binding with/without AOA. (I) Venn diagram identifying PHRF1 by intersecting PSAT1 IP–MS proteins, predicted BCLAF1-interacting proteins, and an E3 ligase database. (J) Co-immunoprecipitation analysis of the interaction between BCLAF1 and PHRF1 following PSAT1 knockdown. (K) Immunoblot analysis of BCLAF1, PHRF1, and PSAT1 following PHRF1 knockdown in PSAT1-deficient cells. (L) CHX chase assay and quantification of BCLAF1 protein stability after PSAT1 and PHRF1 depletion. (M) BCLAF1 ubiquitination analysis following PSAT1 and PHRF1 knockdown in the presence of MG132. (N) Immunoblot analysis of GPX4, HSPA5, and PSAT1 in PSAT1-overexpressing cells with or without HSPA5 knockdown. Luciferase activity was normalized to the corresponding control and is presented as mean ± SD. ns, not significant.

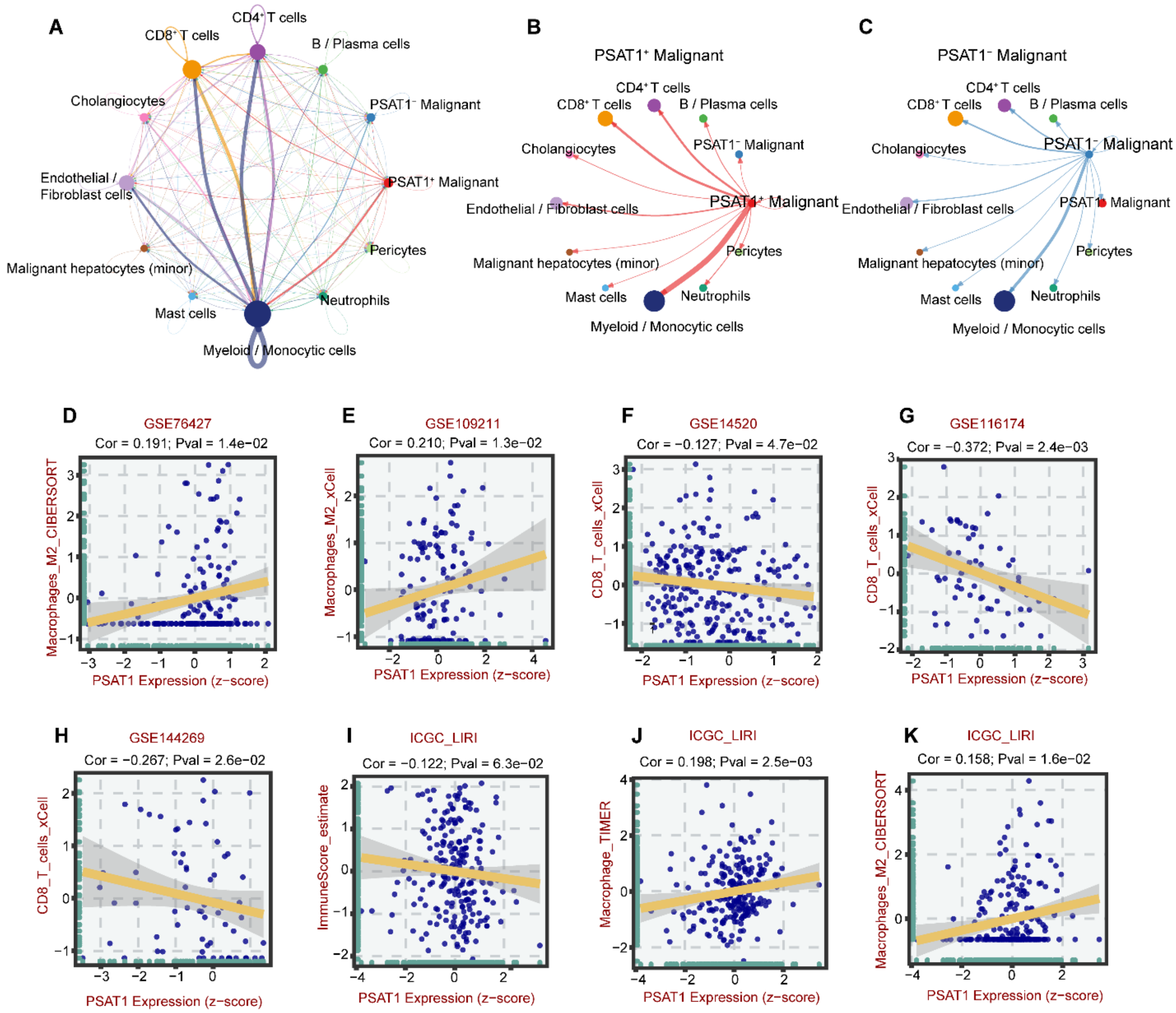

A
CD4⁺ T cells
CD8⁺ T cells
B / Plasma cells
Cholangiocytes
PSAT1⁻ Malignant
Endothelial / Fibroblast cells
PSAT1⁺ Malignant
Pericytes
Malignant hepatocytes (minor)
Neutrophils
Mast cells
Myeloid / Monocytic cells
B
PSAT1⁺ Malignant
C
PSAT1⁻ Malignant
D
GSE76427
Cor = 0.191; Pval = 1.4e−02
Macrophages_M2_CIBERSORT
PSAT1 Expression (z−score)
E
GSE109211
Cor = 0.210; Pval = 1.3e−02
Macrophages_M2_xCell
F
GSE14520
Cor = −0.127; Pval = 4.7e−02
CD8_T_cells_xCell
G
GSE116174
Cor = −0.372; Pval = 2.4e−03
CD8_T_cells_xCell
H
GSE144269
Cor = −0.267; Pval = 2.6e−02
CD8_T_cells_xCell
I
ICGC_LIRI
Cor = −0.122; Pval = 6.3e−02
ImmuneScore_estimate
J
ICGC_LIRI
Cor = 0.198; Pval = 2.5e−03
Macrophage_TIMER
K
ICGC_LIRI
Cor = 0.158; Pval = 1.6e−02
Macrophages_M2_CIBERSORT

Figure S8. PSAT1 is associated with an immunosuppressive tumor microenvironment in HCC. (A) Cell–cell communication network inferred from single-cell RNA-seq data, depicting global intercellular interactions among major cell populations in HCC. Node size represents the overall interaction strength of each cell type, and edge thickness indicates the strength of ligand–receptor interactions. (B) Outgoing signaling network of $PSAT1^{+}$ malignant hepatocytes, illustrating ligand-mediated communication from $PSAT1^{+}$ tumor cells to other cell populations. (C) Outgoing signaling network of $PSAT1^{-}$ malignant hepatocytes, showing ligand–receptor interactions from $PSAT1^{-}$ tumor cells to surrounding cell types. Edge thickness in (B) and (C) reflects interaction strength. (D–H) Correlation analysis between PSAT1 expression and immune cell infiltration across multiple independent HCC transcriptomic datasets (GSE76427, GSE109211, GSE14520, GSE116174, and GSE144269). PSAT1 expression (z-score) was correlated with estimated macrophage or $CD8^{+}$ T-cell infiltration. Each dot represents an individual tumor sample. Solid lines indicate linear regression fits with shaded areas denoting 95% confidence intervals. Pearson correlation coefficients (Cor) and P values are shown. (I–K) Correlation of PSAT1 expression with immune-related signatures in the ICGC-LIRI-JP cohort. PSAT1 expression (z-score) was correlated with overall immune score, macrophage infiltration estimated by TIMER, and M2 macrophage abundance inferred by CIBERSORT. Each dot represents an individual tumor sample, with linear regression lines and 95% confidence intervals indicated. Pearson correlation coefficients and corresponding P values are shown.

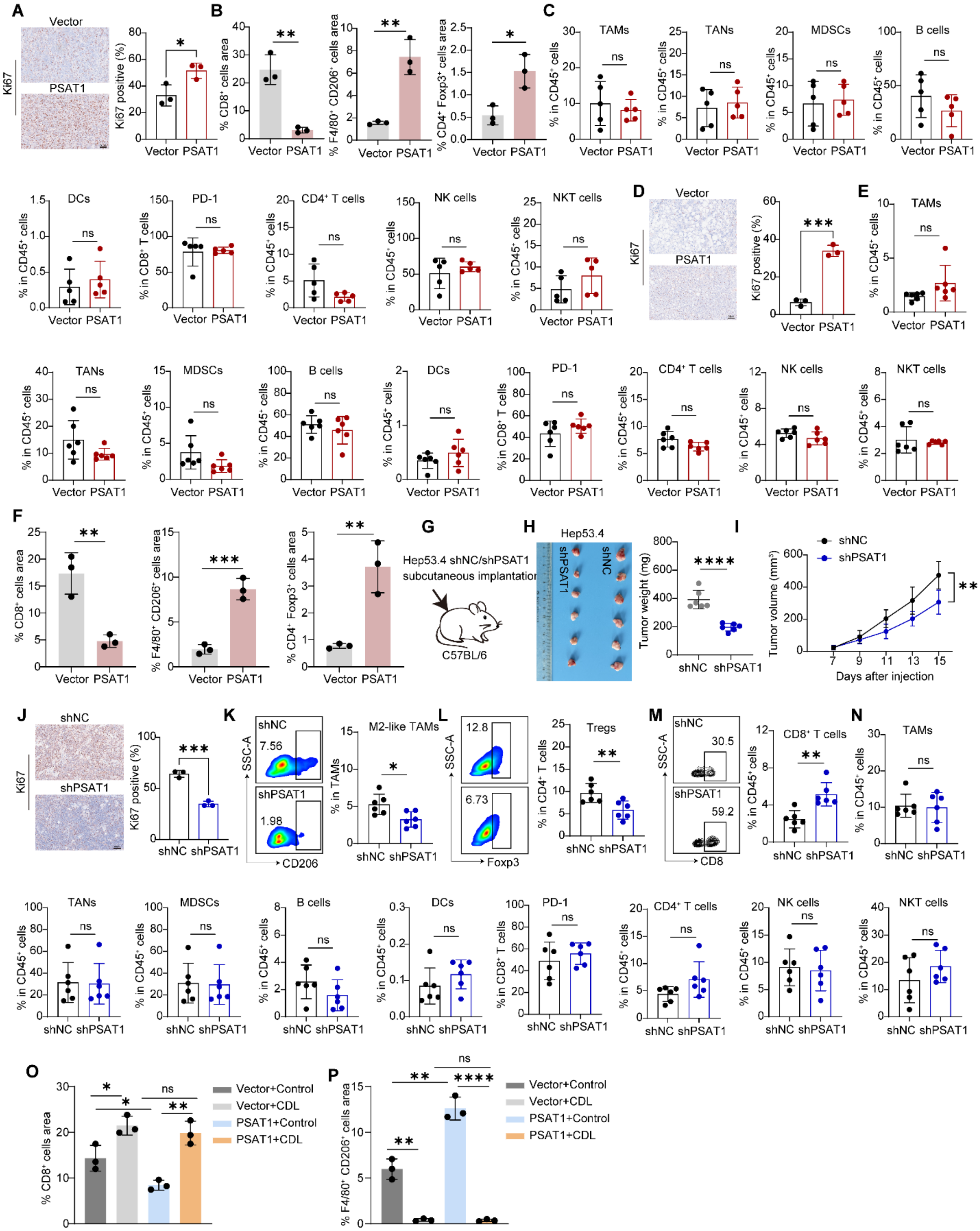
A
Vector
PSAT1
Ki67
Ki67 positive (%)
B
% CD8+ cells area
% F4/80+ CD206+ cells area
% CD4+ Foxp3+ cells area
C
TAMs
TANs
MDSCs
B cells
DCs
PD-1
CD4+ T cells
NK cells
NKT cells
% in CD45+ cells
% in CD8+ T cells
ns
D
E
F
G
Hep53.4 shNC/shPSAT1
subcutaneous implantation
C57BL/6
H
Hep53.4
shPSAT1
shNC
Tumor weight (mg)
I
shNC
shPSAT1
Tumor volume (mm3)
Days after injection
J
K
SSC-A
CD206
7.56
1.98
M2-like TAMs
% in TAMs
L
12.8
6.73
Foxp3
Tregs
% in CD4+ T cells
M
30.5
59.2
CD8
CD8+ T cells
N
O
Vector+Control
Vector+CDL
PSAT1+Control
PSAT1+CDL
P

Figure S9. PSAT1 promotes an immunosuppressive tumor microenvironment in HCC. (A) Representative immunohistochemical staining for Ki67 in orthotopic Hepa1-6 tumors derived from mice implanted with vector control or PSAT1-overexpressing cells, with quantification of Ki67-positive tumor cells shown on the right. (B) Quantification of $CD8^+$ T-cell, $F4/80^+CD206^+$ M2 macrophage, and $CD4^+Foxp3^+$ Treg areas in tumor sections from the indicated groups. (C) Flow cytometric analysis of tumor-infiltrating immune cell populations in orthotopic Hepa1-6 tumors from the indicated groups. The frequencies of TAMs, TANs, MDSCs, B cells, DCs, $PD\text{-}1^+$ $CD8^+$ T cells, $CD4^+$ T cells, NK cells, and NKT cells are shown. (D) Representative Ki67 immunohistochemical staining in liver tumors from hydrodynamic tail-vein injection (HDI)–induced HCC models expressing vector control or PSAT1, with quantification shown on the right. (E) Flow cytometric analysis of tumor-infiltrating immune cell populations in HDI-induced HCC tumors expressing vector control or PSAT1, including TAMs, TANs, MDSCs, B cells, DCs, $PD\text{-}1^+$ $CD8^+$ T cells, $CD4^+$ T cells, NK cells, and NKT cells. (F) Quantification of $CD8^+$ T-cell, $F4/80^+CD206^+$ M2 macrophage, and $CD4^+Foxp3^+$ Treg areas in tumor sections from the HDI-induced HCC models. (G) Schematic illustration of the subcutaneous tumor model. Hep53.4 cells stably expressing control shRNA (shNC) or PSAT1-targeting shRNA (shPSAT1) were subcutaneously implanted into C57BL/6 mice. (H) Representative images of excised subcutaneous tumors derived from shNC or shPSAT1 Hep53.4 cells, with tumor weights quantified on the right. (I) Tumor growth curves of subcutaneous Hep53.4 tumors expressing shNC or shPSAT1 at the indicated time points after implantation. (J) Representative Ki67 immunohistochemical staining of subcutaneous Hep53.4 tumors expressing shNC or shPSAT1, with quantification shown on the right. (K–M) Representative flow cytometry plots and quantification of M2-like TAMs (K), Tregs (L), and $CD8^+$ T cells (M) in subcutaneous Hep53.4 tumors expressing shNC or shPSAT1. (N) Flow cytometric analysis of additional tumor-infiltrating immune cell populations in shNC and shPSAT1 subcutaneous Hep53.4 tumors, including TAMs, TANs, MDSCs, B cells, DCs, $PD\text{-}1^+$ $CD8^+$ T cells, $CD4^+$ T cells, NK cells, and NKT cells. (O–P) Quantification of $CD8^+$ T-cell area (O) and $F4/80^+CD206^+$ M2 macrophage area (P) in tumors derived from vector-control or PSAT1-overexpressing cells following treatment with control or clodronate liposomes (CDL). Data are presented as mean ± SD. ns, not significant; $*P < 0.05$; $**P < 0.01$; $***P < 0.001$; $****P < 0.0001$.

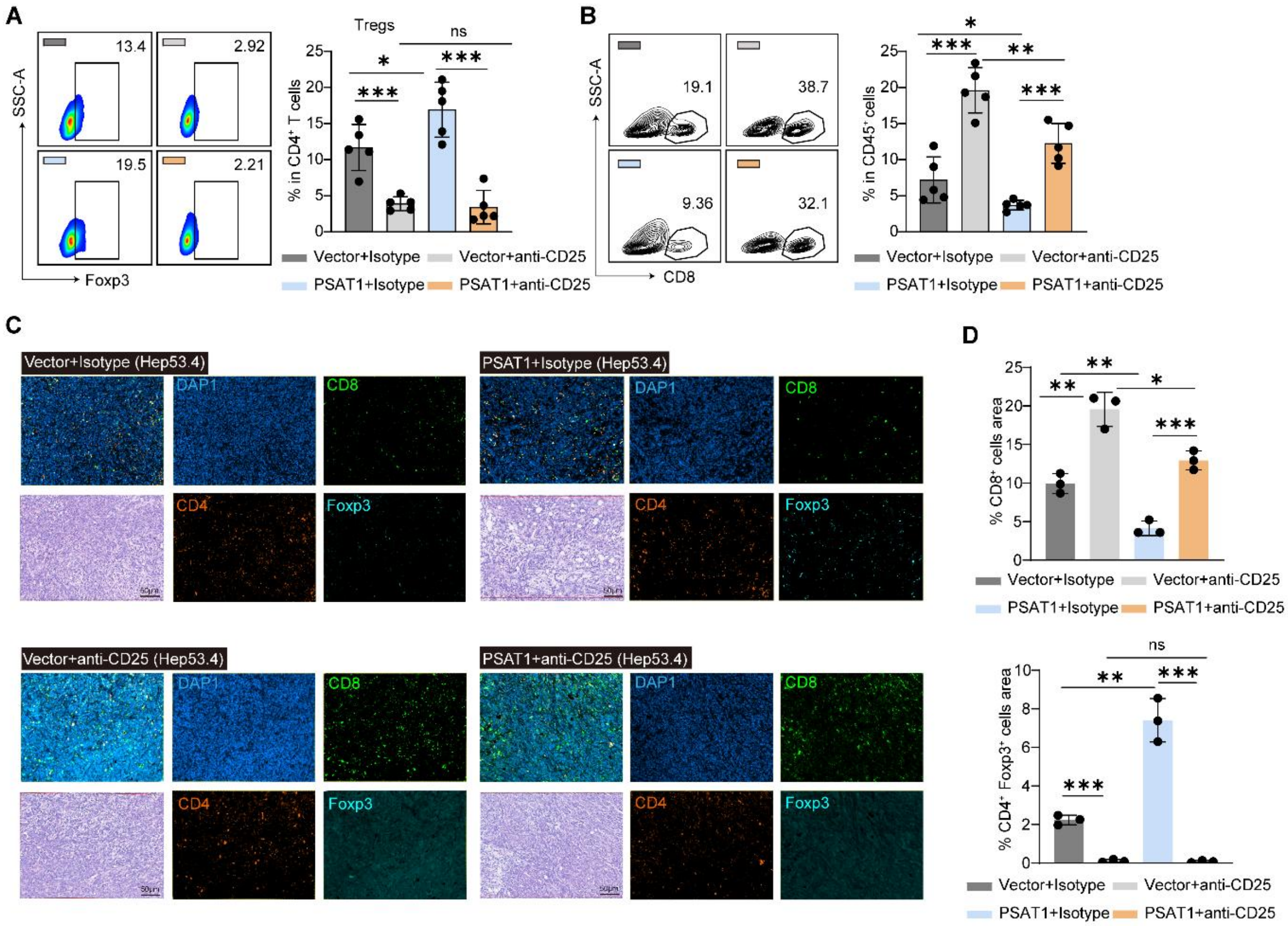
A
SSC-A
Foxp3
13.4
2.92
19.5
2.21
Tregs
% in CD4+ T cells
Vector+Isotype
Vector+anti-CD25
PSAT1+Isotype
PSAT1+anti-CD25
B
SSC-A
CD8
19.1
38.7
9.36
32.1
% in CD45+ cells
C
Vector+Isotype (Hep53.4)
PSAT1+Isotype (Hep53.4)
Vector+anti-CD25 (Hep53.4)
PSAT1+anti-CD25 (Hep53.4)
DAPI
CD8
CD4
Foxp3
D
% CD8+ cells area
% CD4+ Foxp3+ cells area

Figure S10. Treg depletion reverses PSAT1-induced immunosuppression in HCC. (A) Representative flow cytometry plots and quantification of intratumoral Treg cells in tumors derived from vector- or PSAT1-overexpressing Hep53.4 cells following treatment with isotype control or anti-CD25 antibody. Treg cells were defined as Foxp3$^+$ cells within the CD4$^+$ T-cell population. (B) Representative flow cytometry plots and quantification of intratumoral CD8$^+$ T cells in tumors from the indicated groups. CD8$^+$ T cells were quantified as a percentage of CD45$^+$ cells. (C) Representative images of tumor sections from the indicated groups, including H&E staining and immunofluorescence staining for DAPI, CD8, CD4, and Foxp3. (D) Quantification of CD8$^+$ cell area and CD4$^+$Foxp3$^+$ cell area in tumor sections from the indicated groups. ns, not significant; *$P < 0.05$; **$P < 0.01$; ***$P < 0.001$.

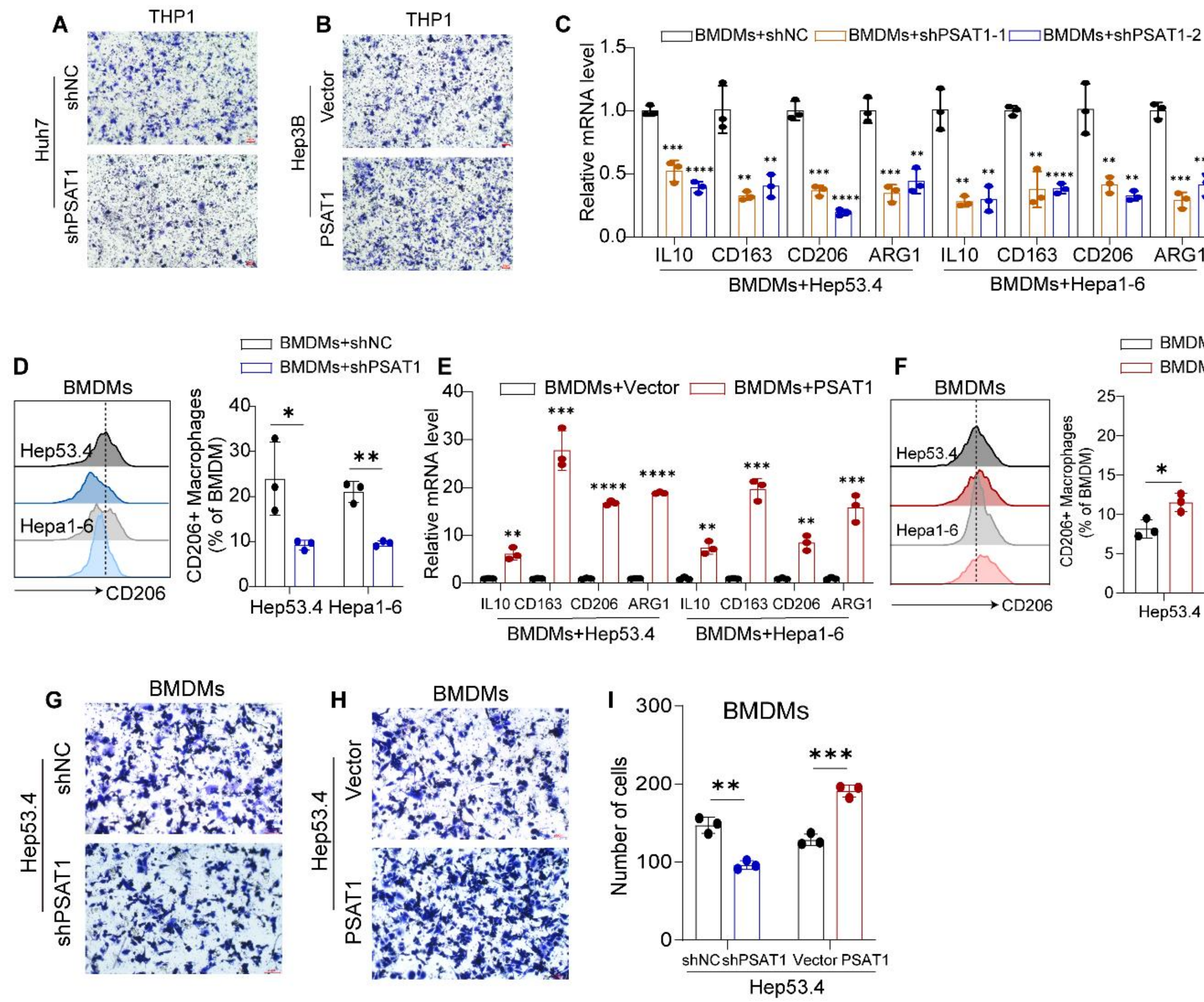

A
THP1
Huh7
shNC
shPSAT1
B
THP1
Hep3B
Vector
PSAT1
C
BMDMs+shNC
BMDMs+shPSAT1-1
BMDMs+shPSAT1-2
Relative mRNA level
IL10
CD163
CD206
ARG1
BMDMs+Hep53.4
BMDMs+Hepa1-6
D
BMDMs
Hep53.4
Hepa1-6
CD206
BMDMs+shNC
BMDMs+shPSAT1
CD206+ Macrophages (% of BMDM)
E
BMDMs+Vector
BMDMs+PSAT1
Relative mRNA level
F
BMDMs
G
BMDMs
Hep53.4
H
BMDMs
I
BMDMs
Number of cells
shNC
shPSAT1
Vector
PSAT1

Figure S11. PSAT1 promotes macrophage migration and M2 polarization.

(A–B) Representative transwell migration assays with THP1-derived macrophages seeded in the upper chamber and Huh7 cells with PSAT1 knockdown (A) or Hep3B cells with PSAT1 overexpression. (B) cultured in the lower chamber. (C) qRT–PCR analysis of M2 macrophage markers IL10, CD163, CD206, and ARG1 in BMDMs co-cultured with Hep53.4 or Hepa1-6 cells expressing shNC or two independent PSAT1-targeting shRNAs. (D) Flow cytometric analysis and quantification of $CD206^+$ macrophages in BMDMs co-cultured with PSAT1-knockdown Hep53.4 or Hepa1-6 cells. (E) qRT–PCR analysis of IL10, CD163, CD206, and ARG1 expression in BMDMs co-cultured with Hep53.4 or Hepa1-6 cells overexpressing PSAT1. (F) Flow cytometric analysis and quantification of $CD206^+$ macrophages in BMDMs co-cultured with PSAT1-overexpressing Hep53.4 or Hepa1-6 cells. (G–H) Representative transwell migration assays with BMDMs seeded in the upper chamber and Hep53.4 cells with PSAT1 knockdown (G) or overexpression (H) cultured in the lower chamber. (I) Quantification of migrated BMDMs in G–H. *$p < 0.05$; **$p < 0.01$; ***$p < 0.001$; ****$p < 0.0001$.

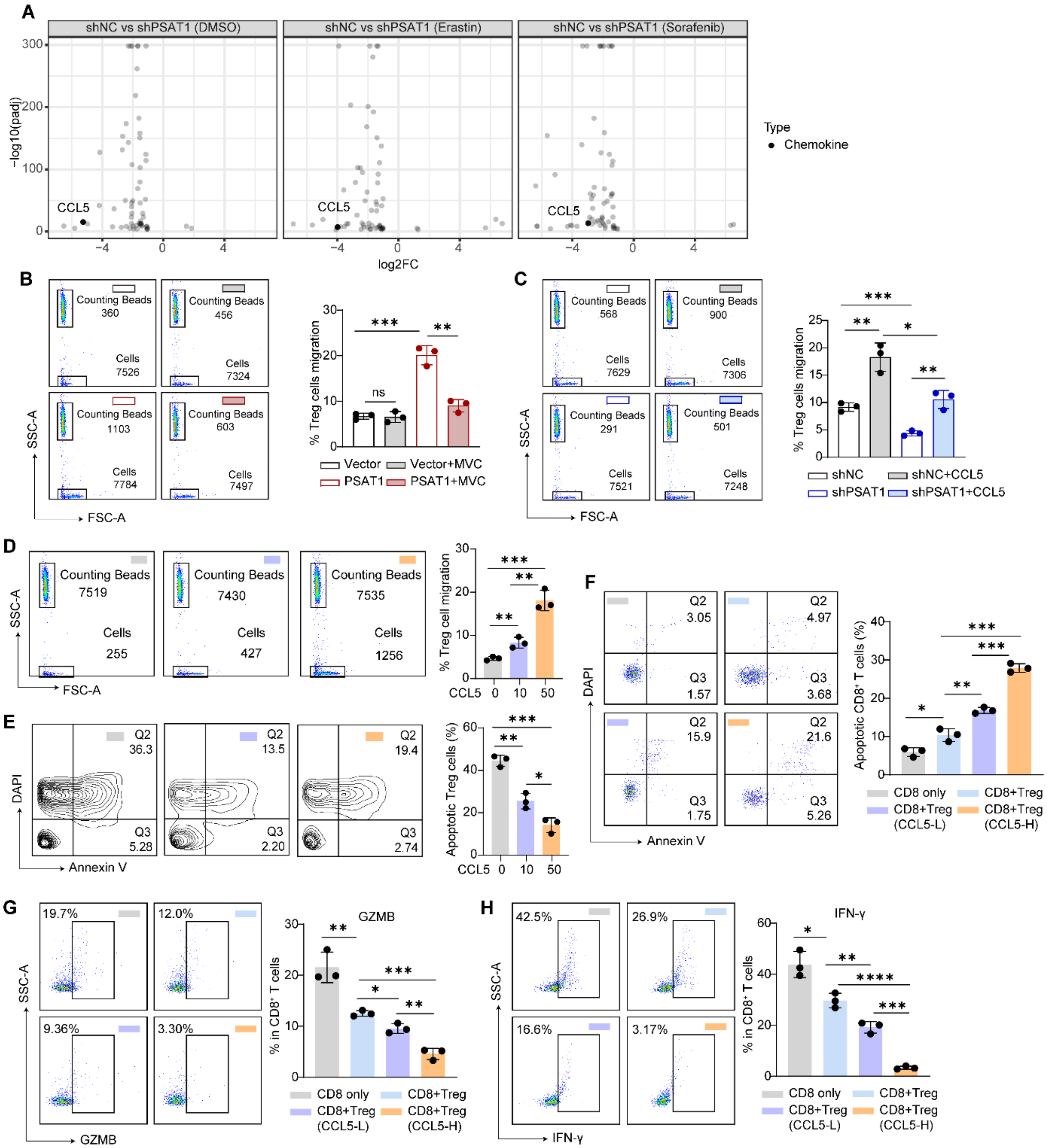

A
shNC vs shPSAT1 (DMSO)
shNC vs shPSAT1 (Erastin)
shNC vs shPSAT1 (Sorafenib)
-log10(padj)
300
200
100
0
CCL5
-4
0
4
log2FC
Type
Chemokine
B
Counting Beads
360
Cells
7526
Counting Beads
456
Cells
7324
Counting Beads
1103
Cells
7784
Counting Beads
603
Cells
7497
SSC-A
FSC-A
% Treg cells migration
***
**
ns
Vector
Vector+MVC
PSAT1
PSAT1+MVC
C
Counting Beads
568
Cells
7629
Counting Beads
900
Cells
7306
Counting Beads
291
Cells
7521
Counting Beads
501
Cells
7248
SSC-A
FSC-A
% Treg cells migration
***
**
*
**
shNC
shNC+CCL5
shPSAT1
shPSAT1+CCL5
D
Counting Beads
7519
Cells
255
Counting Beads
7430
Cells
427
Counting Beads
7535
Cells
1256
SSC-A
FSC-A
% Treg cell migration
CCL5 0 10 50
E
Q2
36.3
Q3
5.28
Q2
13.5
Q3
2.20
Q2
19.4
Q3
2.74
DAPI
Annexin V
Apoptotic Treg cells (%)
CCL5 0 10 50
F
Q2
3.05
Q3
1.57
Q2
4.97
Q3
3.68
Q2
15.9
Q3
1.75
Q2
21.6
Q3
5.26
DAPI
Annexin V
Apoptotic CD8+ T cells (%)
CD8 only
CD8+Treg
CD8+Treg (CCL5-L)
CD8+Treg (CCL5-H)
G
19.7%
12.0%
9.36%
3.30%
SSC-A
GZMB
GZMB
% in CD8+ T cells
CD8 only
CD8+Treg
CD8+Treg (CCL5-L)
CD8+Treg (CCL5-H)
H
42.5%
26.9%
16.6%
3.17%
SSC-A
IFN-γ
IFN-γ
% in CD8+ T cells
CD8 only
CD8+Treg
CD8+Treg (CCL5-L)
CD8+Treg (CCL5-H)

Figure S12. PSAT1–CCL5 signaling promotes Treg recruitment and immunosuppressive function. (A) Volcano plots showing differentially expressed genes between shNC and shPSAT1 cells under DMSO, erastin, or sorafenib treatment, with chemokine-related genes highlighted. (B) Treg migration toward conditioned media from PSAT1-overexpressing HCC cells with or without maraviroc treatment. (C) Treg migration toward conditioned media from PSAT1-knockdown HCC cells with or without recombinant CCL5 supplementation. (D) Dose-dependent effect of recombinant CCL5 on Treg migration. (E) Flow cytometric analysis of Treg apoptosis following treatment with increasing concentrations of CCL5. (F) Apoptosis of $CD8^+$ T cells co-cultured with untreated or CCL5-pretreated Tregs. (G-H) Flow cytometric analysis of GZMB and IFN-γ expression in $CD8^+$ T cells co-cultured with untreated or CCL5-pretreated Tregs. *$p < 0.05$; **$p < 0.01$; ***$p < 0.001$.

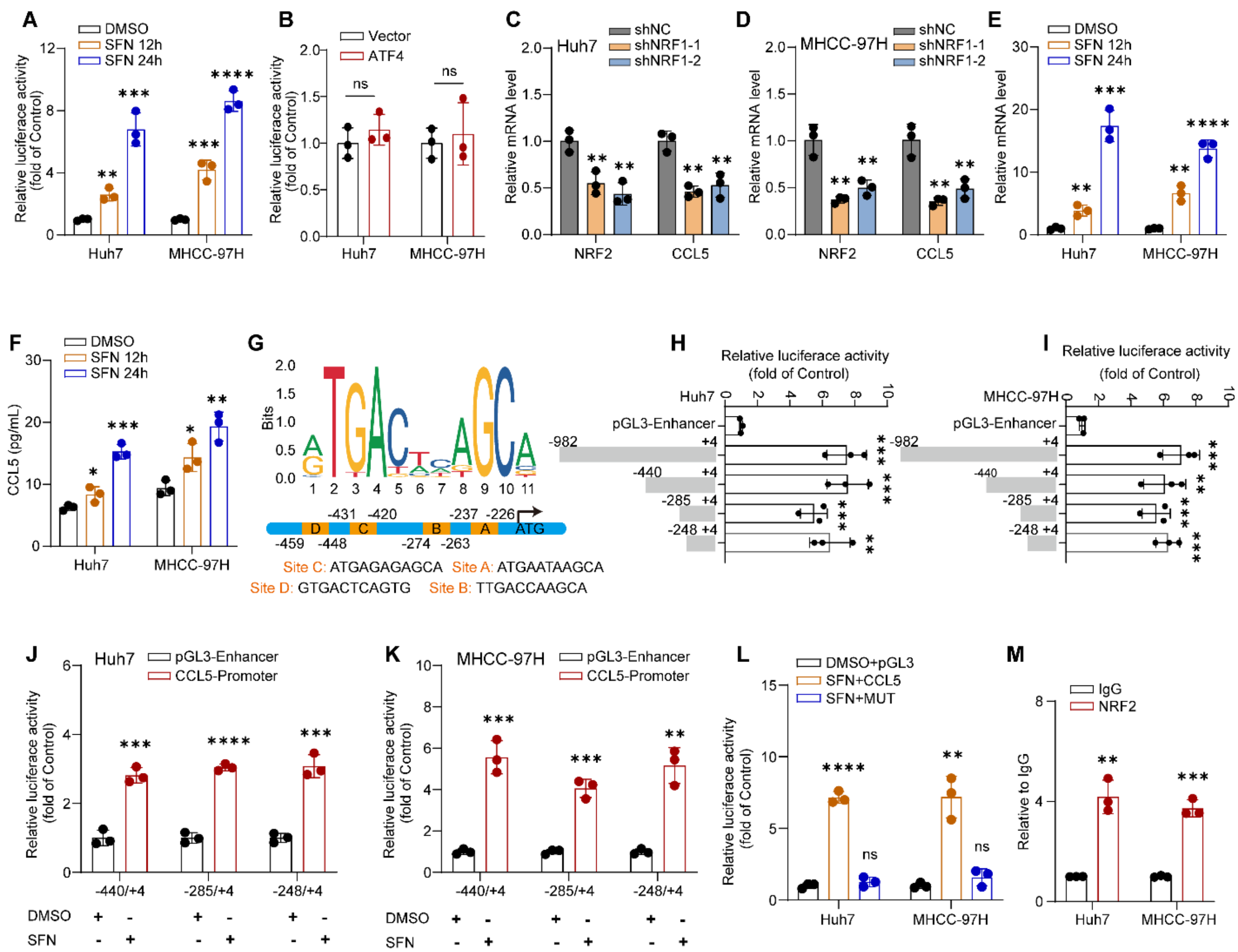
A
DMSO
SFN 12h
SFN 24h
Relative luciferase activity (fold of Control)
Huh7
MHCC-97H
B
Vector
ATF4
ns
C
Huh7
shNC
shNRF1-1
shNRF1-2
Relative mRNA level
NRF2
CCL5
D
MHCC-97H
E
F
CCL5 (pg/mL)
G
Bits
Site C: ATGAGAGAGCA
Site A: ATGAATAAGCA
Site D: GTGACTCAGTG
Site B: TTGACCAAGCA
H
I
pGL3-Enhancer
J
K
pGL3-Enhancer
CCL5-Promoter
-440/+4
-285/+4
-248/+4
DMSO
SFN
L
DMSO+pGL3
SFN+CCL5
SFN+MUT
M
IgG
NRF2
Relative to IgG

Figure S13. PSAT1 promotes CCL5 expression through NRF2. (A–B) Luciferase reporter assays assessing CCL5 promoter activity following SFN treatment or ATF4 overexpression. (C–D) qRT–PCR analysis of NRF2 and CCL5 mRNA levels after NRF2 knockdown. (E–F) CCL5 mRNA expression and secreted protein levels following SFN treatment. (G) Predicted NRF2-binding antioxidant response element (ARE) within the human CCL5 promoter. (H–I) Luciferase assays using serial 5′-truncated CCL5 promoter constructs. (J–K) SFN responsiveness of truncated CCL5 promoter fragments. (L) Luciferase assays comparing wild-type and NRF2-binding site–mutant CCL5 promoter constructs with or without SFN treatment. (M) ChIP–qPCR analysis of NRF2 occupancy at the CCL5 promoter. $^{*}p < 0.05$; $^{**}p < 0.01$.

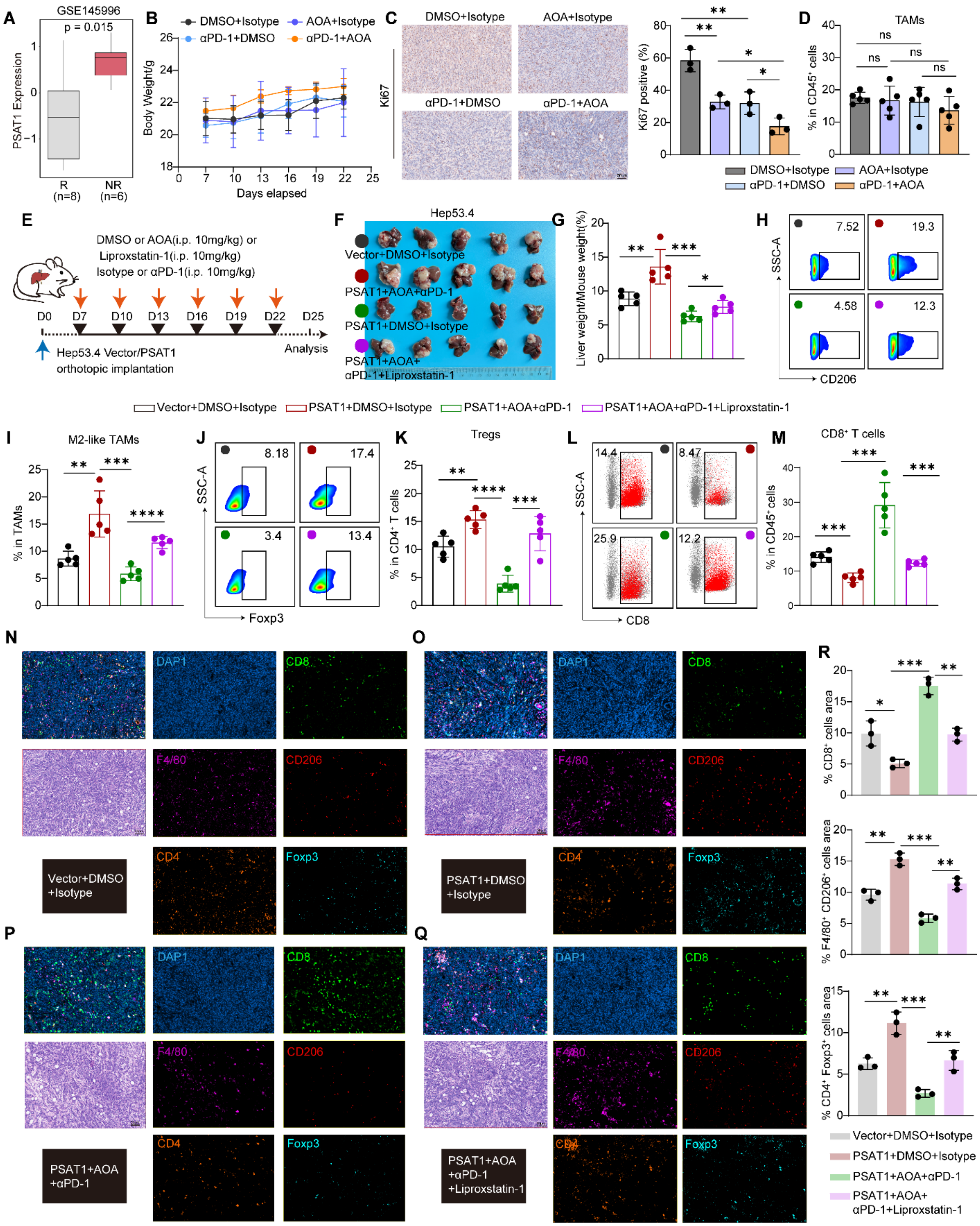

A
GSE145996
p = 0.015
PSAT1 Expression
R (n=8)
NR (n=6)
B
DMSO+Isotype
AOA+Isotype
αPD-1+DMSO
αPD-1+AOA
Body Weight/g
Days elapsed
C
DMSO+Isotype
AOA+Isotype
αPD-1+DMSO
αPD-1+AOA
Ki67
Ki67 positive (%)
D
TAMs
% in CD45+ cells
E
DMSO or AOA(i.p. 10mg/kg) or
Liproxstatin-1(i.p. 10mg/kg)
Isotype or αPD-1(i.p. 10mg/kg)
D0 D7 D10 D13 D16 D19 D22 D25
Hep53.4 Vector/PSAT1
orthotopic implantation
Analysis
F
Hep53.4
Vector+DMSO+Isotype
PSAT1+AOA+αPD-1
PSAT1+DMSO+Isotype
PSAT1+AOA+
αPD-1+Liproxstatin-1
G
Liver weight/Mouse weight(%)
H
SSC-A
CD206
7.52
19.3
4.58
12.3
Vector+DMSO+Isotype
PSAT1+DMSO+Isotype
PSAT1+AOA+αPD-1
PSAT1+AOA+αPD-1+Liproxstatin-1
I
M2-like TAMs
% in TAMs
J
SSC-A
Foxp3
8.18
17.4
3.4
13.4
K
Tregs
% in CD4+ T cells
L
SSC-A
CD8
14.4
8.47
25.9
12.2
M
CD8+ T cells
% in CD45+ cells
N
DAPI
CD8
F4/80
CD206
CD4
Foxp3
Vector+DMSO
+Isotype
O
PSAT1+DMSO
+Isotype
P
PSAT1+AOA
+αPD-1
Q
PSAT1+AOA
+αPD-1
+Liproxstatin-1
R
% CD8+ cells area
% F4/80+ CD206+ cells area
% CD4+ Foxp3+ cells area
Vector+DMSO+Isotype
PSAT1+DMSO+Isotype
PSAT1+AOA+αPD-1
PSAT1+AOA+
αPD-1+Liproxstatin-1

Figure S14. AOA sensitizes tumors to anti-PD-1 blockade via ferroptosis induction. (A) PSAT1 expression in responders and non-responders from the indicated anti–PD-1–treated cohort. (B) Body weight changes of mice receiving DMSO, AOA, anti–PD-1, or the combination treatment. (C) Representative Ki67 immunohistochemical staining and quantification in orthotopic tumors. (D) Flow cytometric quantification of total tumor-associated macrophages under the indicated treatment conditions. (E) Schematic of the orthotopic Hep53.4 HCC model and treatment schedule with AOA, anti–PD-1, and liproxstatin-1. (F–G) Representative liver tumors and liver weight-to-body weight ratios from the indicated groups. (H–I) Representative flow cytometric plots and quantification of M2-like TAMs. (J–K) Representative flow cytometric plots and quantification of tumor-infiltrating Tregs. (L–M) Representative flow cytometric plots and quantification of intratumoral $CD8^+$ T cells. (N–Q) Representative multiplex immunofluorescence images showing $CD8^+$ T cells, $F4/80^+CD206^+$ M2-like macrophages, and $CD4^+Foxp3^+$ Tregs in orthotopic tumors from the indicated treatment groups. (R) Quantification of $CD8^+$ T-cell, $F4/80^+CD206^+$ macrophage, and $CD4^+Foxp3^+$ Treg areas in tumor sections from mice receiving the indicated treatments. Data are shown as mean ± SD. *$p < 0.05$; **$p < 0.01$.

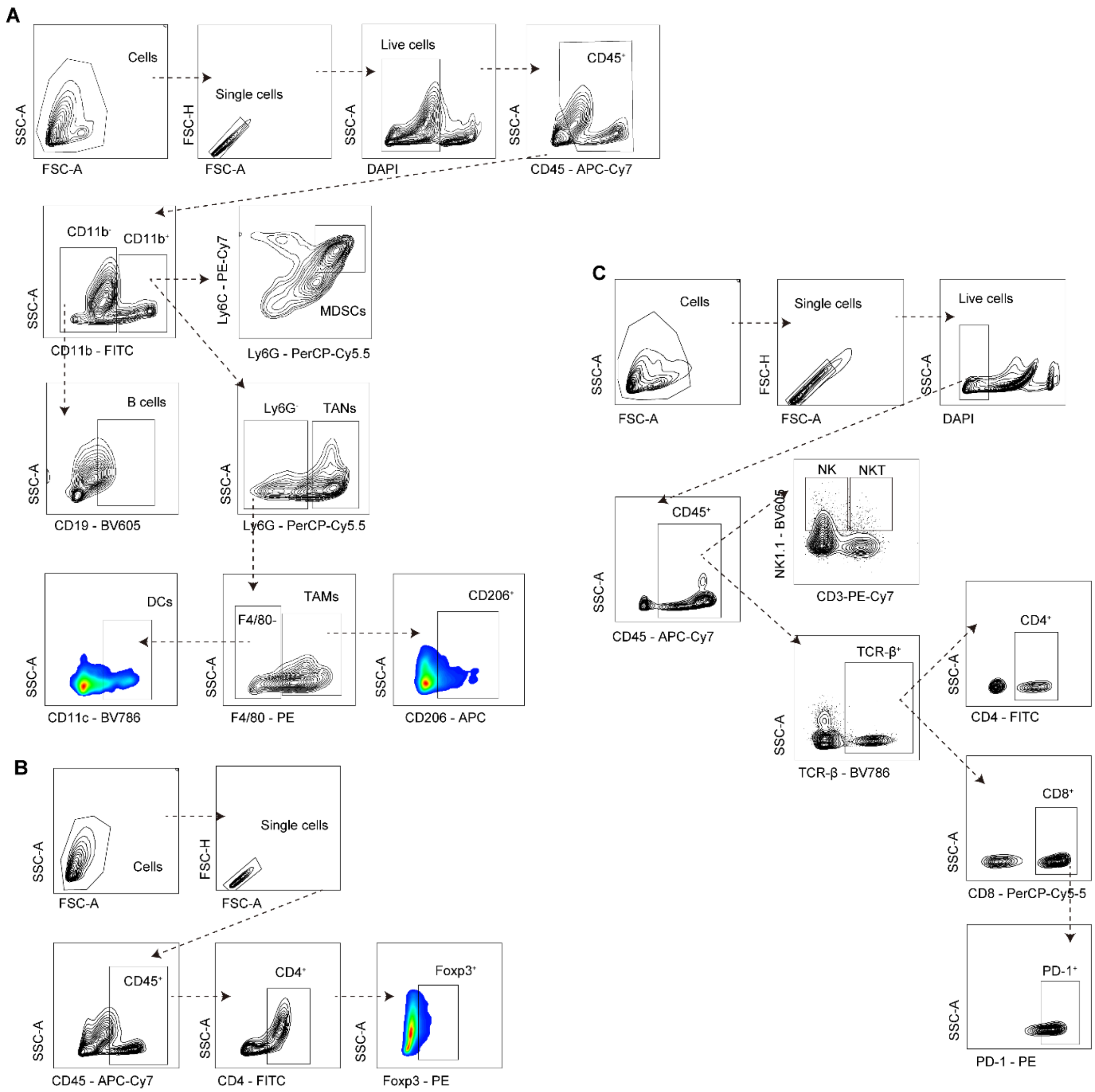
A
Cells
SSC-A
FSC-A
Single cells
FSC-H
FSC-A
Live cells
SSC-A
DAPI
CD45⁺
SSC-A
CD45 - APC-Cy7
CD11b⁻
CD11b⁺
SSC-A
CD11b - FITC
Ly6C - PE-Cy7
MDSCs
Ly6G - PerCP-Cy5.5
B cells
SSC-A
CD19 - BV605
Ly6G⁻
TANs
SSC-A
Ly6G - PerCP-Cy5.5
DCs
SSC-A
CD11c - BV786
TAMs
F4/80-
SSC-A
F4/80 - PE
CD206⁺
SSC-A
CD206 - APC
B
SSC-A
Cells
FSC-A
Single cells
FSC-H
FSC-A
CD45⁺
SSC-A
CD45 - APC-Cy7
CD4⁺
SSC-A
CD4 - FITC
Foxp3⁺
SSC-A
Foxp3 - PE
C
Cells
SSC-A
FSC-A
Single cells
FSC-H
FSC-A
Live cells
SSC-A
DAPI
CD45⁺
SSC-A
CD45 - APC-Cy7
NK
NKT
NK1.1 - BV605
CD3-PE-Cy7
TCR-β⁺
SSC-A
TCR-β - BV786
CD4⁺
SSC-A
CD4 - FITC
CD8⁺
SSC-A
CD8 - PerCP-Cy5-5
PD-1⁺
SSC-A
PD-1 - PE

Figure S15. Flow cytometry gating strategy for tumor-infiltrating immune cells. (A) Gating strategy for myeloid cell subsets isolated from tumor tissues. After exclusion of debris (FSC-A/SSC-A), doublets, and dead cells ($DAPI^-$), CD45+ leukocytes were selected. $CD11b^+$ myeloid cells were further subdivided into $Ly6C^+Ly6G^-$ myeloid-derived suppressor cells (MDSCs), $Ly6G^+$ tumor-associated neutrophils (TANs), and $F4/80^+$ tumor-associated macrophages (TAMs). M2-like TAMs were identified based on CD206 expression. $CD11b^-$ cells were gated to identify $CD19^+$ B cells and $CD11c^+$ dendritic cells (DCs). (B) Gating strategy for regulatory T cells (Tregs). Following exclusion of debris, doublets, and dead cells, $CD45^+CD4^+$ T cells were gated and Foxp3 expression was used to define Tregs. (C) Gating strategy for T-cell and NK-cell subsets. After live singlet gating, $CD45^+$ cells were selected. NK and NKT cells were identified based on NK1.1 and CD3 expression. $TCR\beta^+$ T cells were further subdivided into $CD4^+$ and $CD8^+$ populations, and PD-1 expression was analyzed within $CD8^+$ T cells.

Table S1. The sequences of shRNA target

Identifier	Forward (5’-3’)
PSAT1-1	GCCAAAGGAGATGATGCTTTA
PSAT1-2	GCCAAGAAGTTTGGGACTATA
BCLAF1-1	GCGGTTCACTTCGTATCAGAA
BCLAF1-2	CCTTCTCAGAATAGTCCAATTC
NRF2-1	GGAGTAAGTCGAGAAGTATTT
NRF2-2	GGATATGGTACAACCCTTGT
PHRF1	CGAGCTCAATTTGGTGGTAAA

Table S2. The sequences of gene-specific primers used for qRT-PCR and vector constructs.

Gene name	Forward(5'-3')	Reverse (5'-3')
**Primers for qRT-PCR**		
PSAT1	TCCTTGTACAACACGCCTCC	GCTCCACTGGACAAACGTAGA
HSPA5	TGCCGTTCAAGGTGGTTGA	GCCAGCAATAGTTCCAGCGT
BCLAF1	CACAGGAGGAACCGAAAGA	GTACTGAGCATGGGAGCATC
IL-10	CTACGGCGCTGTCATCGAT	AGTCGCCACCCTGATGTCTC
CD163	CCGGGAGATGAATTCTTGCCT	AGACACAGAAATTAGTTCAGCAGCA
GAPDH	AGAAGGCTGGGGCTCATTTG	AGGGGCCATCCACAGTCTTC
CD206	CTGAATTGTACTGGTCTGTCCT	GCTTAGATGTGGTGCTGTGG
ARG1	CGGAGACCACAGTTTGGCAA	ACCCAGGAGAATCCTGGCAC
DDIT3	GATCCAACTGCAGAGATGGC	GTGACCTCTGCTGGTTCTGG
PPP1R15A	TGAAGAGGAGGCTGAAGACA	GTCCTTATCAGAAGGCTGGG
HYOU1	CCACTGCCCAGAATATCATG	GCACATCCTTTGCTCTCTGA
HERPUD1	ACATCTCAAGGCCTGAAGCT	ACTGGTTGTGAATAGGGGCT
SEL1L	CAAGGGACAGACTGCTCTTG	GGCAGATTCACAACTCTGGA
CCL5	TGCCTCCCCATATTCCTCG	AGCTTGCCCTTGTTCAGCC
mARG1	CTCCAAGCCAAAGTCCTTAG	AGGAGCTGTCATTAGGGACA
mCD206	AGCTATTGGACGCGAGGCA	GGTGACGGAAGCCCAGTCAGT
mIL10	CTGCCTTCAGCCAGGTGAAGA	TGCCTGGGGCATCACTTCTAC
mCD163	GCTGTCACTAACGCTCCTGGA	CTGGCAAACCACGGACACTT
mβ-actin	TGACGTTGACATCCGTAAAGA	GCCGGACTCATCGTACTCC
HSPA5-F0	GACGCCGGCCAAGACAG	CTTGCCAGCCAGTTGGGCA
HSPA5-F1	ACACTGATCTGCTAGTGCTG	ACACACCCTAACCCAGGT
HSPA5-F2	GGGAGAAGAAGAATTGGCC	TCACCCCACACACGAGTATC
HSPA5-F3	GACTAGATACTCGTGTGTGGG	CCCTTGCCTGAGTAAAGAT

HSPA5-F4	CATCTTTACTCAGGCAAGGG	GTGATCACTCACTCCCCATC
HSPA5-F5	GATGGGGAGTGAGTGATCAC	TTTGACACAAACCATTGCC
HSPA5-F6	CCCCTCACTATGAATGGG	TAGAGATGGAGTTTCACCATG
HSPA5-F7	GCCAACATGGTGAAACTCCA	CCTTTCAGCCAGTTGCCC
HSPA5-F8	GGGCAACTGGCTGAAAGG	TGAGAGCCTTTGAGGTAAGGG
HSPA5-F9	AACTAGGGCTAGTCTTCC	TTGAAGTCTATCTTTTTAATATT
**Primers for vector constructs**		
PSAT1 (mouse)	ATGGAAGCCACCAAGCAAG	TCACAGCTGATGCATCTCCA
CCL5 promoter (-982/+3)	GTGCCCAAGAAGGCCTTAC	CATGGTACCTGTGGGAGAGG
CCL5 promoter (-440/+3)	GAAGACTCAGTGACTCAGTG	CATGGTACCTGTGGGAGAGG
CCL5 promoter (-248/-)	GCTCACTCTAGATGAGAGAG	CATGGTACCTGTGGGAGAGG
CC5 promoter (-285/+3)	CCACCATTGGTGCTTGGT	CATGGTACCTGTGGGAGAGG
HSPA5 promoter (-521/-220)	GCGGAAATGCCTCGGGGT	CGGCGTCGACCTCACCGT
**Primers for ChIP**		
CCL5 promoter for NRF2 – ChIP-1	CTCTCTGAGGAGGACCCCTT	AATAGCAACCAAGCATTGGC
CCL5 promoter for NRF2– ChIP-2	TCCACCATTGGTGCTTGGTC	CAAGCATTGGCCGGTATCAT

Table S3. Antibodies used in this study

Antibody	catalog	Dilution	Company
**For Western blotting**			
PSAT1	10501-1-ap	1:5000	Proteintech
HSPA5	MA5-19151	1:1000	Invitrogen
BCLAF1	26809-1-AP	1:1000	Proteintech
PERK	20582-1-AP	1:1000	Proteintech
p-PERK	29546-1-AP	1:500	Proteintech
NRF2	16396-1-AP	1:1000	Proteintech
SLC7A11	12691S	1:1000	CST
ATF4	10835-1-AP	1:1000	Proteintech
PD-L1	13684S	1:500	CST
STAT1	9172	1:1000	CST
p-STAT1	7649	1:500	CST
STAT6	5397	1:1000	CST
p-STAT6	9361	1:500	CST
PHRF1	ab85974	1:500	Abcam
Ub	10201-2-AP	1:500	Proteintech
β-actin	A3854	1:10000	Sigma
Secondary antibody	HRP conjugated goat anti-rabbit IgG	1:5000	Proteintech
Secondary antibody	HRP conjugated goat anti-mouse IgG	1:5000	Proteintech
**Forflow cytometry**			
TruStainFcX™ PLUS	156604	1:100	BioLegend
Brilliant Violet 785™ anti-mouse CD11c	115539	1:100	BioLegend
Brilliant Violet 605™ anti-mouse CD19	115539	1:100	BioLegend
PE/Cyanine7 anti-mouse Ly-6C	128018	1:100	BioLegend
PE/Cyanine7 anti-mouse CD3ε	100320	1:100	BioLegend
Brilliant Violet 785™ anti-mouse TCR β chain	109249	1:100	BioLegend

PerCP/Cyanine5.5 anti-mouse CD8a	100734	1:100	BioLegend
APC anti-mouse CD69	104514	1:100	BioLegend
PE anti-mouse CD279	135206	1:100	BioLegend
Brilliant Violet 605™ anti-mouse NK-1.1	108739	1:100	BioLegend
PE anti-mouse F4/80	123110	1:100	BioLegend
APC/Cyanine7 anti-mouse CD45	103116	1:100	BioLegend
APC anti-mouse CD206	141708	1:100	BioLegend
PE anti-mouse/rat/human FOXP3	320008	1:100	BioLegend
FITC anti-mouse CD4	100406	1:100	BioLegend
PerCP/Cyanine5.5 anti-mouse Ly-6G	127615	1:100	BioLegend
APC anti-mouse CD25 Antibody	102011	1:100	BioLegend
PE anti-mouse CD127 (IL-7Rα) Antibody	135009	1:100	BioLegend
PE/Cyanine5 anti-human/mouse Granzyme B Recombinant	372226	1:100	BioLegend
Brilliant Violet 510™ anti-mouse IFN-γ Antibody	505841	1:100	BioLegend
**For Immunohistochemistry**			
4-HNE	MAB3249	1:100	R&D
Ki67	ab16667	1:500	Abcam
MDA	ab243066	1:500	Abcam
Secondary antibody	Envision kit (HRP, rabbit/mouse, DAB+)	Ready-to-use	DAKO
**For Immunofluorescence staining**			
CD8	14-0081-82	1:100	Thermo

CD206	ab64693	1:100	Abcam
Foxp3	85128-6-RR	1:100	Proteintech
CD4	A26036PM	1:100	Abclone
F4/80	14-4801-82	1:100	Invitrogen
TSA Fluorescence Triple Staining Plus Kit	RK50026P		Abclone
